# Attenuation of PM_2.5_-induced alveolar epithelial cells and lung injury through regulation of mitochondrial fission and fusion

**DOI:** 10.1186/s12989-023-00534-w

**Published:** 2023-07-18

**Authors:** Qi Liu, Jiali Weng, Chenfei Li, Yi Feng, Meiqin Xie, Xiaohui Wang, Qing Chang, Mengnan Li, Kian Fan Chung, Ian M Adcock, Yan Huang, Hai Zhang, Feng Li

**Affiliations:** 1 Department of Pulmonary and Critical Care Medicine, Shanghai Chest Hospital, Shanghai Jiao Tong University School of medicine, NO.241, West Huaihai Road, 200030 Shanghai, P.R. China; 2 Airway Disease Section, National Heart and Lung Institute, Imperial College, Dovehouse Street, SW3 6LY London, UK; 3 School of Pharmacy, Anhui Medical University, 230022 Hefei, Anhui China

**Keywords:** Mitochondrial fission, Mitochondrial fusion, PM_2.5_, Mitophagy, Mitochondrial oxygen consumption rate (OCR), Mitochondrial morphology, Type II alveolar epithelial cell (AECII)

## Abstract

**Background:**

Exposure to particulate matter (PM) with an aerodynamic diameter less than 2.5 μm (PM_2.5_) is a risk factor for developing pulmonary diseases and the worsening of ongoing disease. Mitochondrial fission and fusion are essential processes underlying mitochondrial homeostasis in health and disease. We examined the role of mitochondrial fission and fusion in PM_2.5_-induced alveolar epithelial cell damage and lung injury. Key genes in these processes include dystrophin-related protein 1 (DRP1) and optic atrophy 1 (OPA1) respectively.

**Methods:**

Alveolar epithelial (A549) cells were treated with PM_2.5_ (32 µg/ml) in the presence and absence of Mdivi-1 (10µM, a DRP1 inhibitor) or BGP-15 (10µM, an OPA1 activator). Results were validated using DRP1-knockdown (KD) and OPA1-overexpression (OE). Mice were injected intraperitoneally with Mdivi-1 (20 mg/kg), BGP-15 (20 mg/kg) or distilled water (control) one hour before intranasal instillation of PM_2.5_ (7.8 mg/kg) or distilled water for two consecutive days.

**Results:**

PM_2.5_ exposure of A549 cells caused oxidative stress, enhanced inflammation, necroptosis, mitophagy and mitochondrial dysfunction indicated by abnormal mitochondrial morphology, decreased mitochondrial membrane potential (ΔΨm), reduced mitochondrial respiration and disrupted mitochondrial fission and fusion. Regulating mitochondrial fission and fusion pharmacologically using Mdivi-1 and BGP-15 and genetically using DRP1-KD and OPA1-OE prevented PM_2.5_-induced celluar damage in A549 cells. Mdivi-1 and BGP-15 attenuated PM_2.5_-induced acute lung injury in mice.

**Conclusion:**

Increased mitochondrial fission and decreased mitochondrial fusion may underlie PM_2.5_-induced alveolar epithelial cell damage in vitro and lung injury in vivo.

**Supplementary Information:**

The online version contains supplementary material available at 10.1186/s12989-023-00534-w.

## Background

Particulate matter with an aerodynamic diameter of ≤2.5 μm (PM_2.5_) is characterized by its small particle size, large surface area and toxin absorption ability [1]. Previous studies have identified associations between PM_2.5_ exposure and increased incidence of pulmonary diseases [2]. The size characteristics of PM_2.5_ make it more likely to enter into the lower respiratory tract, penetrate into the alveolar space and even ultimately into the systemic circulation [3, 4]. Lung inflammation, oxidative stress and mitochondrial damage may be induced by low dose of PM_2.5_ in mice [5–7]. PM_2.5_ has been demonstrated to increase the expression of pro-inflammatory cytokine and their receptors, which results in activation of various pro-inflammatory signaling pathways within cells of the airways and lung [8]. PM_2.5_ increases the levels of intracellular reactive oxygen species (ROS), activates the nuclear translocation of NF-E2-related factor-2 (Nrf2), and inhibits the activities of superoxide dismutase, catalase and glutathione peroxidase [9].

Biomass-related PM_2.5_ has detrimental effects on human airway epithelial cells, leading to mitochondrial dysfunction, abnormal mitochondrial metabolism and altered mitochondrial dynamics [10]. PM_2.5_ also induces morphological damage to mitochondria, particularly causing abnormalities in mitochondrial structure such as mitochondrial swelling, cristae disorder and even fragmentation [11].

Mitochondrial dynamics include mitochondrial fission and fusion processes that assist in reshaping, rebuilding and recycling of mitochondria to allow compensation of cellular stress [12]. Mitochondrial fission in mammals is mediated by dynamin-related protein 1 (DRP1), which interacts with four mitochondrial receptor proteins: fission 1 (Fis1), mitochondria fission factor (MFF), mitochondrial dynamics protein of 49 kDa (MID49) and MID51. The fusion of outer mitochondrial membranes (OMM) is mediated by mitofusin 1 (MFN1) and MFN2, while the fusion of inner mitochondrial membranes (IMM) is mediated by optic atrophy 1 (OPA1)[13]. We previously demonstrated that PM_2.5_ could increase the expression of DRP1 and MFF and decrease that of MFN2 and OPA1 in mice [7]. Due to the essential role of mitochondrial fission and fusion in mitochondrial quality control, mitochondrial fission inhibitors such as Mdivi-1 and mitochondrial fusion activators such as BGP- 15 have been developed and used in research [14, 15].

The removal of damaged mitochondria through autophagy, a process called mitophagy, is critical for maintaining proper cellular functions and is instigated by the mitochondrial serine/threonine kinase PTEN induced putative kinase 1 (PINK1) [16]. Recombinant Parkinson Disease protein 2 (PARK2) is a cytosolic E3 ubiquitin ligase that translocates to depolarized mitochondria, associates with PINK1 and initiates mitophagy. Following PARK2-mediated ubiquitination of outer mitochondrial membrane proteins, the selective mitophagy adapter protein sequestosome-1 (SQSTM1/p62) is recruited to mitochondria and then directly interacts with microtubule-associated protein 1 A/1B-light chain 3 (LC3) via its LC3 interacting region to enhance mitophagy [13].

Apoptosis denotes a genetically programmed pathway involving caspase activation, while necrosis is induced by extreme physical or chemical stress [17]. Recent studies suggest the existence of a genetically programmed and regulated form of necrosis termed necroptosis [18]. Mitophagy can activate necroptosis via the necrosome which is composed of the receptor-interacting protein kinase (RIPK) 1 and 3 together with the mixed-lineage kinase domain-like protein (MLKL)[19]. Necroptosis is a strong inducer of inflammation that releases extremely high levels of damage-associated molecular patterns (DAMPs)[20].

We hypothesized that PM_2.5_ exposure disrupts the mitochondrial fission and fusion balance in alveolar epithelial cells and lungs, leading to ROS responses and pro-inflammatory responses, which may be attenuated by regulating mitochondrial fission and fusion. We therefore examined these effects by PM_2.5_ on alveolar epithelial (A549) cells in vitro and in murine lung in vivo and whether PM_2.5_ actions were prevented by pharmacological and genetic modulation of mitochondrial fission and fusion.

## Materials and methods

### PM_2.5_ sampling, extraction, and chemical analysis

The collection, extraction and component analysis of PM_2.5_ have been described in a previous study [7]. PM_2.5_ samples were collected on quartz filters (Tissuquartz,Pall, USA) using a high flow volume PM_2.5_ Sampler (Ecotech,Australia) at a flow rate of 1.13 m^3^/min, located on the top of a building in Xuhui District in Shanghai, China, from September 2017 to April 2018. The filters were cut into small fragments (1 cm × 1 cm), then immersed into ultrapure deionized water and eluted with an ultrasonic cleaner (KUDOS, Shanghai, China), followed by freeze-drying with a vacuum freeze dryer (Four-Ring Science Instrument Plant, Beijing, China). PM_2.5_ solid particulates were preserved at -80 °C until required.

### Cell culture, pharmacologic and genetic intervention

A549 cells (Shanghai Institutes for Biological Sciences, China Academy of Science, Shanghai) were cultured in F12K medium (Procell Life Science & Technology, Wuhan, China) with 10% fetal bovine serum, 100U/ml penicillin and 100 µg/ml streptomycin at 37 °C in 5% CO_2_.

In this study, 0, 2.5, 5, 10, 20 and 40 µg/cm^2^ PM2.5 correspond to 0, 8, 16, 32, 64 and 128 µg/mL, respectively. We mainly use six-well plates, confocal dishes and 96-well plates for our experiments. 3 ml of medium is added per well for six-well plates (9.6 cm²) and confocal dishes (9.6 cm²) and 100 µl of medium per well for 96-well plates (0.32 cm²).

A549 cells were treated with 32 µg/ml PM_2.5_ to induce cell injury. Cells were pretreated with 10µM of Mdivi-1 (a DRP1 inhibitor) (Selleck, Shanghai, China) or 10µM BGP-15 (an OPA1 activator) (Selleck, Shanghai, China) for 2 h and then were cultured with vehicle or PM_2.5_ for 48 h (preliminary study results are shown in Figure S1). Human DRP1-knockdown(KD) plasmid and OPA1-overexpression(OE) plasmid sequences were commercially designed (Lncbio-technology, Xuhui, Shanghai, China). Lentivirus packaging was performed in 293T cells using Zorin virus packaging kit (Shanghai Zorin Biological Technology, Shanghai, China). After the lentivirus was prepared, the cells were stably transfected using Polybrene, screened using antibiotic puromycin dihydrochloride (Shanghai Zorin Biological Technology, Fengxian, Shanghai, China) resistance to obtain DRP1- KD and OPA1-OE cells. These cells were also treated with 32 µg/ml PM_2.5_ for 48 h to induce injury.

### Animal experiments

10-week-old male C57/BL6 mice, weight 22-25 g, were purchased from SPF Biotechnology Co., Ltd (Shanghai, China). All the mice were housed in a specific pathogen-free facility where the circulating temperature is 22 °C with 50–60% humidity, equal light-dark cycle, and with access to standard food and water ad libitum. All experimental studies involving animals were approved by the laboratory animal ethics committee of the institute. According to a preliminary study, mice were administered intraperitoneally with Mdivi-1 (20 mg/kg) or BGP-15 (20 mg/kg) (both were dissolved in saline including 5% dimethyl sulfoxide (DMSO), 40% polyethylene glycol 300 (PEG 300) and 5% Tween 80), one hour before intranasal instillation of 50µL of PM_2.5_ suspension (7.8 mg/kg) (the PM_2.5_ concentration was selected according to the results of a previous study[7], and Mdivi-1 and BGP-15 concentrations were selected according to the results of the preliminary study in Figure S2) or distilled water once a day for two consecutive days.

### Cell proliferation assay

The effect of vehicle or PM_2.5_ on cell proliferation was measured by BeyoClick™ EdU Cell Proliferation Kit with Diaminobenzidine (DAB) staining (Beyotime Technology, Haimen, Jiangsu, China). Cells were cultured in a 6-well plate (3ml per well of culture medium) with a density of 5 × 10^4^cells/ml overnight at 37 °C before exposure to vehicle or PM_2.5_. After 48 h of co-culture, cells were cultured in the presence of EdU for 2 h, after which they were fixed with paraformaldehyde and permeabilized with a detergent, which allows the detection reagents to access the EdU-labeled DNA. The incorporated EdU was detected with a horseradish peroxidase (HRP) -conjugated antibody that binds to an azide group on the EdU molecule. This was followed by addition of the DAB substrate, which produces a brown precipitate at the site of EdU incorporation. The samples were measured under microscopy to visualize the brown precipitate at the site of EdU incorporation, as well as the counterstained nuclei.

### Quantitative reverse transcription polymerase chain reaction (RT-PCR)

Cells were cultured using 6-well plates (3ml per well of culture medium) with a density of 5 × 10^4^cells/ml. Total RNA was extracted from A549 cells using TRIzol and then its concentration and purity were assessed. One µg total RNA was reverse transcribed into cDNA and quantitative real-time PCR was performed with ChamQ Universal SYBR qPCR Master Mix (Vazyme, Nanjing, Jiangsu, China) in an ABI ViiATM 7 System (Applied Biosystems, Foster City, CA, USA). The reaction conditions included 95 °C for 30s, followed by 40 cycles of 95 °C for 10s and 60 °C for 30s, with a final cycle of 95 °C for 15s, 60 °C for 60s and 95 °C for 15s. The primer sequences of the cytokines (IL-1β, IL-6, IL-18 and CXCL-8) and β-actin are shown in Table 1.

**Table 1:** Primer sequences of cytokines and β-actin

IL- 1β	Forward Reverse	5’- TCGCAGCAGCACATCAACAAGAG − 3’ 5’- AGGTCCACGGGAAAGACACAGG − 3’
IL-6	Forward Reverse	5’- CACTGGTCTTTTGGAGTTTGAG − 3’ 5’- GGACTTTTGTACTCATCTGCAC − 3’
IL-18	Forward Reverse	5’- GCTGAAGATGATGAAAACCTGG − 3 5’- CAAATAGAGGCCGATTTCCTTG − 3’
CXCL8	Forward Reverse	5’- AACTGAGAGTGATTGAGAGTGG − 3’ 5’- ATGAATTCTCAGCCCTCTTCAA − 3’
β-actin	Forward Reverse	5’- GGCCAACCGCGAGAAGATGAC − 3’ 5’- GGATAGCACAGCCTGGATAGCAAC − 3’

### Measurement of intracellular and mitochondrial ROS in cells

Intracellular ROS generation was detected using 2’,7’-Dichlorodihydrofluorescein diacetate (DCFH-DA) (Sigma-Aldrich, St. Louis, MO, USA). Briefly, the cells were seeded in 96-well black plates(100 µl per well of culture medium) with a density of 5 × 10^4^ /ml with 6 parallel wells in each group. Cells were stained with 10µM DCFH-DA at 37 °C in the dark for 15 min. Then cells were washed with serum-free F12K for three times, the level of ROS was determined using a fluorescence plate reader (Molecular Devices, San Jose, CA, USA) at 488/525nm. Mitochondrial ROS(mtROS) level was measured using MitoSOX Red (Invitrogen, Life Technologies, Carlsbad, CA, USA). Briefly, the cells were seeded in 96-well black plates with a density of 5 × 10^4^ /ml with 6 parallel wells in each group. Cells were incubated with 5mmol/L MitoSOX Red probe for 10 min at 37 °C. The cells were washed twice with PBS, and red fluorescence was determined at 510/580nm using a fluorescence plate reader (Molecular Devices).

### Mitochondrial morphology in cells

Mitochondrial morphology was measured with Mito-Tracker Green FM assay (Invitrogen) according to the manufacturer’s instructions. Cells were seeded in confocal dishes (3ml of medium per well) with a density of 5 × 10^4^cells/ml, and incubated with 500 µl of 200nM Mito-Tracker Green FM (Invitrogen) 30 min at 37 °C in the dark and washed twice with serum-free medium. 100 µl of Hoechst 33,342 (Beyotime) was added and incubated for 30 min at 37 °C. The mitochondrial morphology was assessed using a confocal microscopy (Leica, Wetzlar, Germany). Cell area and mitochondrial area were measured by ImageJ software (https://imagej.nih.gov/ij/). Mitochondrial fragmentation was judged to occur if > 90% of mitochondria in the cytoplasm outside of the perinuclear compaction were punctate or circular, and mitochondrial perinuclear compaction was judged to be present if > 90% of mitochondria accumulated in the perinuclear area[19].

### Mitochondrial membrane potential (ΔΨm) in cells

Cells were seeded in 6-well plates (2 × 10^5^ cells/well, 3ml of medium per well) and cultured with 1mL JC-1 staining working solution (Beijing Solarbio Science & Technology Co., Ltd.) for 20 min. The cells were later washed twice using PBS. Cells were photographed under an inverted fluorescence microscope(Leica). Red fluorescence was determined at 579/599nm and green fluorescence was determined at 490/516nm. The relative ΔΨm level was evaluated by comparing the red fluorescence cells to green fluorescence cells in the groups.

### Measurement of mitochondrial oxygen consumption rate (OCR) in cells

Cells were seeded on Seahorse XFe96 plates (100 µl of medium per well) at a density of 10,000 cells/well in complete F12K. The OCR was measured using an XFe96 Extracellular Flux Analyzer (Seahorse Bioscience, North Billerica, MA, USA) according to the manufacturer’s instructions. The following inhibitors were added sequentially: 1.5µM oligomycin (an adenosine-triphosphate (ATP) uncoupler), 1µM carbonyl cyanide p-trifluoromethoxyphenylhydrazone (an electron transfer chain (ETC) accelerator), 0.5µM antimycin A (a complex III inhibitor), and 0.5µM rotenone (a complex I inhibitor). OCR was calculated using the standard XFe96 Extracellular Flux Analyzer protocol.

### Transmission electron microscopy (TEM) analysis in cells

The cell culture medium was discarded without washing and then quickly 2.5% glutaraldehyde as fixative was added to the cells. The cells were gently scraped along one direction and then collected (to avoid damaging the cells). The cells were centrifued and added with the fresh fixative and then fixed at room temperature for 2 h. Dehydration was accomplished with increasing concentrations of ethanol (30–100%) and then replaced with propylene oxide for 10 min. The dehydrated cells were immersed in a mixture of propylene oxide and SPI-pon812 embedding agent (SPI supplies, West Chester, PA, USA) overnight at room temperature for soaking and embedding. Ultrathin Sect. (70-80 nm) were obtained by using a diamond knife (Nidau, Switzerland) and Leica EM UC7 ultramicrotome (Wetzlar, Germany). Finally, the ultrathin sections were stained with uranyl acetate and lead citrate. The samples were treated and analyzed for transmission electron microscopy imaging(JEOL-1400 flash, Akishima, Tokyo, Japan). The morphology was qualified by Image J analysis (National Institute of Health, Bethesda, USA).

### Airway hyperresponsiveness (AHR) in mice

After anesthesia with an intraperitoneal injection of 0.2mL 1% pentobarbital, mice were tracheostomized and placed in a plethysmograph before inhaling of aerosolised acetylcholine (ACh) for the measurement of airway resistance and compliance (EMMS, Hants, UK). Lung resistance (RL) was recorded and presented as the percentage change from baseline RL of nebulizing PBS. The concentration of ACh required to increase lung resistance by 100% from baseline was calculated as PC100, and -logPC100 was taken as a measure of airway responsiveness.

### Histological analysis and immunofluorescence in mice

The whole lung was removed, and the right lung was dissected and snap-frozen in liquid nitrogen for later analysis. The left lung was inflated with 4% paraformaldehyde under 25 cm of water pressure and then embedded in paraffin. Paraffin blocks were sectioned to expose the maximum surface area of the lung tissue in the plane of the bronchial tree. Four µm sections were cut and stained with hematoxylin and eosin (H&E). The extent of lung inflammation was evaluated in the H&E-stained lung sections as described previously [21] using the following scale: 0 = no inflammatory response, 1 = mild inflammation with foci of inflammatory cells in the bronchial or vascular wall and in alveolar septa, 2 = moderate inflammation with patchy inflammation or localized inflammation in walls of the bronchi or blood vessels and alveolar septa and less than 1/3 of the lung cross-sectional area is involved, and 3 = severe inflammation with diffuse inflammatory cells in walls of the bronchi or blood vessels and alveoli septa; between one-third and two-thirds of the lung area are involved.

The localization and expression of pulmonary surfactant-associated protein C (SFTPC) was examined by immunofluorescence staining. Lung sections were incubated with anti-SFTPC primary antibody (1:200, Proteintech, Wuhan, Hubei, China) at 4 °C overnight, and the secondary antibody (Goat anti-rabbit IgG (Alexa Fluor 488) ,1:500, Abcam, Cambridge, MA, USA) at room temperature in the dark for 60 min. Nuclear staining was performed with 4’,6-diamidino-2-phenylindole (DAPI) (Beyotime) staining solution, and imaging was obtained.

### Western blot analysis in cells and mice

Total protein from A549 cells cultured in 6-well plates and mice lung tissues were respectively homogenized with a RIPA lysis buffer (Beyotime), and protein concentrations were quantified by a Bicinchoninic Acid (BCA) assay kit (Thermo Fisher Scientific, Waltham, MA, USA). 30 µg of protein per lane was separated through 10–15% sodium dodecyl sulfate polyacrylamide gel electrophoresis (SDS-PAGE) gel and transferred to polyvinylidene fluoride (PVDF) membrane. The membranes were blocked with 5% nonfat milk and incubated with the following primary antibodies: DRP1 (1:1000, Cell Signaling Technology, Danvers, MA,USA), MFF (1:1000, Cell Signaling Technology), MFN2 (1:1000, Cell Signaling Technology), OPA1 (1:1000, Cell Signaling Technology), phosphorylated (phospho) DRP1 (p-DRP1)(1:1000, Cell Signaling Technology), PINK1 (1:1000, Abcam), PARK2 (1:1000, Abcam), SQSTM1/p62 (1:1000, Abcam), LC3 (1:500, Abcam), MLKL (1:1000, Affinity Biosciences, Liyang, Jiangsu, China), RIPK1 (1:1000, Cell Signaling Technology), RIPK3 (1:1000, Cell Signaling Technology), overnight at 4 °C. Membranes were then incubated with a HRP-conjugated anti-rabbit secondary antibody (Cell Signaling Technology,USA) and then visualized by chemiluminescent detection.

### Statistical analysis

Data were presented as mean ± SD. GraphPad Prism 8 was used to compare multiple groups by One-way ANOVA with Bonferroni’s post hoc test (for equal variance) or Dunnett’s T3 post hoc test (for unequal variance).Two-way ANOVA was performed for comparisons of %change in lung resistance between individual groups. P < 0.05 was considered significant.

## Results

### Effect of PM_2.5_, Mdivi-1, BGP-15, DRP1- KD and OPA1- OE on cell proliferation, intracellular and mitochondrial ROS

EdU was used to evaluate cellular proliferation, while DCFH-DA and MitoSOX Red were employed to measure levels of intracellular and mitochondrial ROS, respectively.

**Fig. 1:**
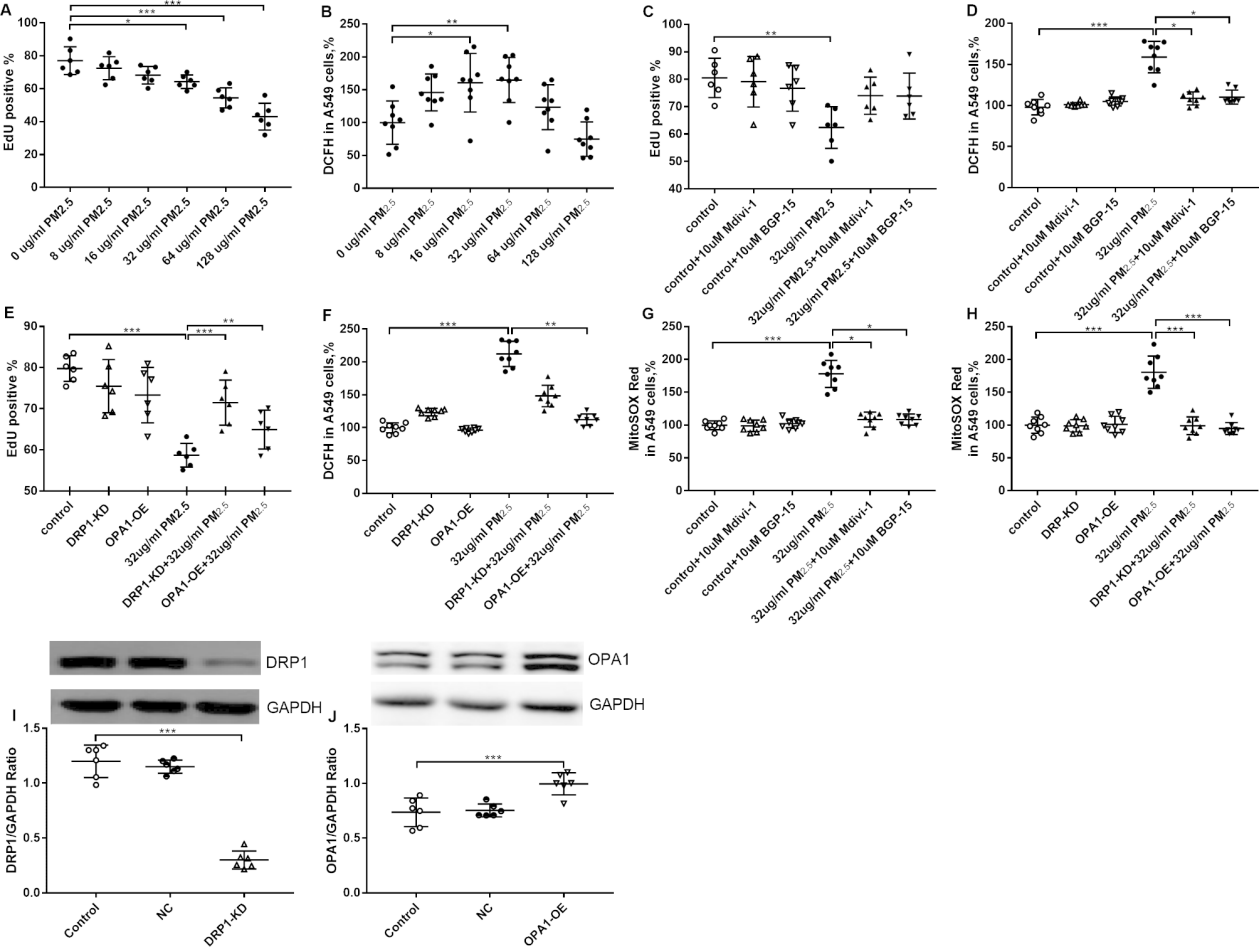
Effect of PM_2.5_, the DRP-1 inhibitor Mdivi-1, the OPA1 activator BGP-15 and of DRP1 knockdown (KD) and OPA1 overexpression (OE) on cell proliferation, intracellular and mitochondrial ROS in A549 cells. Cell proliferation was measured using EdU assays (**A**) and intracellular oxidative stress measured by DCFH (**B**) in A549 cells. Effect the DRP-1 inhibitor Mdivi-1 and the OPA-1 activator BGP-15 alone or in the presence of PM_2.5_ on cell proliferation (**C**) and on intracellular redox-responses (**D**). Effect of DRP1-KD and OPA1-OE on baseline and PM_2.5_–modulated cell proliferation (**E**) and on intracellular redox-responses (**F**) in A549 cells. Effect of PM2.5 in the presence and absence of Mdivi-1 and BGP-15 on mitochondrial superoxide (**G**) and of DRP1-KD and OPA1-OE on PM_2.5_-evoked mitochondrial superoxide (**H**) in A549 cells. The knockdown of DRP1 (**I**) and overexpression of OPA1 (**J**) in A549 cells was confirmed by Western blot. Mean values and data from individual experiments are shown. One-way ANOVA with Bonferroni’s post hoc test (for equal variance) or Dunnett’s T3 post hoc test (for unequal variance) was performed for comparisons among multiple groups. *P < 0.05, **P < 0.01, ***P < 0.001. NC = negative control shRNA, DRP1-KD = DRP1 knockdown, OPA1-OE = OPA1 overexpression

The proliferation of A549 cells was inhibited as measured by EdU assay at PM_2.5_ concentrations of 32, 64 and 128 µg/ml (Fig. 1A, Figure S3). Concentrations of PM_2.5_ at 16 µg/ml and 32 µg/ml increased intracellular ROS levels (Fig. 1B). The proliferation of A549 cells was inhibited at 32 µg/ml PM_2.5_ exposure, and pretreatment with Mdivi-1 or BGP-15 showed trends towards increasing the percentage of EdU positive cells (Fig. 1C). PM_2.5_ significantly enhanced intracellular ROS which was prevented by Mdivi-1 or BGP-15 (Fig. 1D). DRP1-KD significantly prevented the decrease in cell proliferation induced by PM_2.5_ exposure (Fig. 1E), but showed a trend towards reducing intracellular ROS (Fig. 1F). OPA1-OE restored PM_2.5_-inhibited cell proliferation (Fig. 1E) while also preventing the PM_2.5_-induced increase in intracellular ROS (Fig. 1F).

Mitochondrial ROS was increased by PM_2.5_ exposure, and was inhibited by pretreatment with Mdivi-1 or BGP-15 in A549 cells (Fig. 1G). In addition, DRP1-KD and OPA1-OE significantly suppressed PM_2.5_–enhanced mitochondrial ROS in A549 cells (Fig. 1H). DRP1-KD and OPA1-OE in cells were confirmed by Western blot (Fig. 1I, J).

As an indicator of cell damage, the EdU assay results suggested that the proliferation of A549 cells was inhibited after PM_2.5_ exposure, and was restored by promotion of mitochondrial fusion and inhibition of mitochondrial fission. Intracellular ROS levels reflect the degree of redox-responses and mitochondrial ROS levels reflect the degree of hyperoxidation, and both were elevated after PM_2.5_ exposure and attenuated by regulation of mitochondrial fission and fusion.

### Effect of PM_2.5_, Mdivi-1, BGP-15, DRP1-KD and OPA1-OE on pro-inflammatory gene mRNA expression

**Fig. 2:**
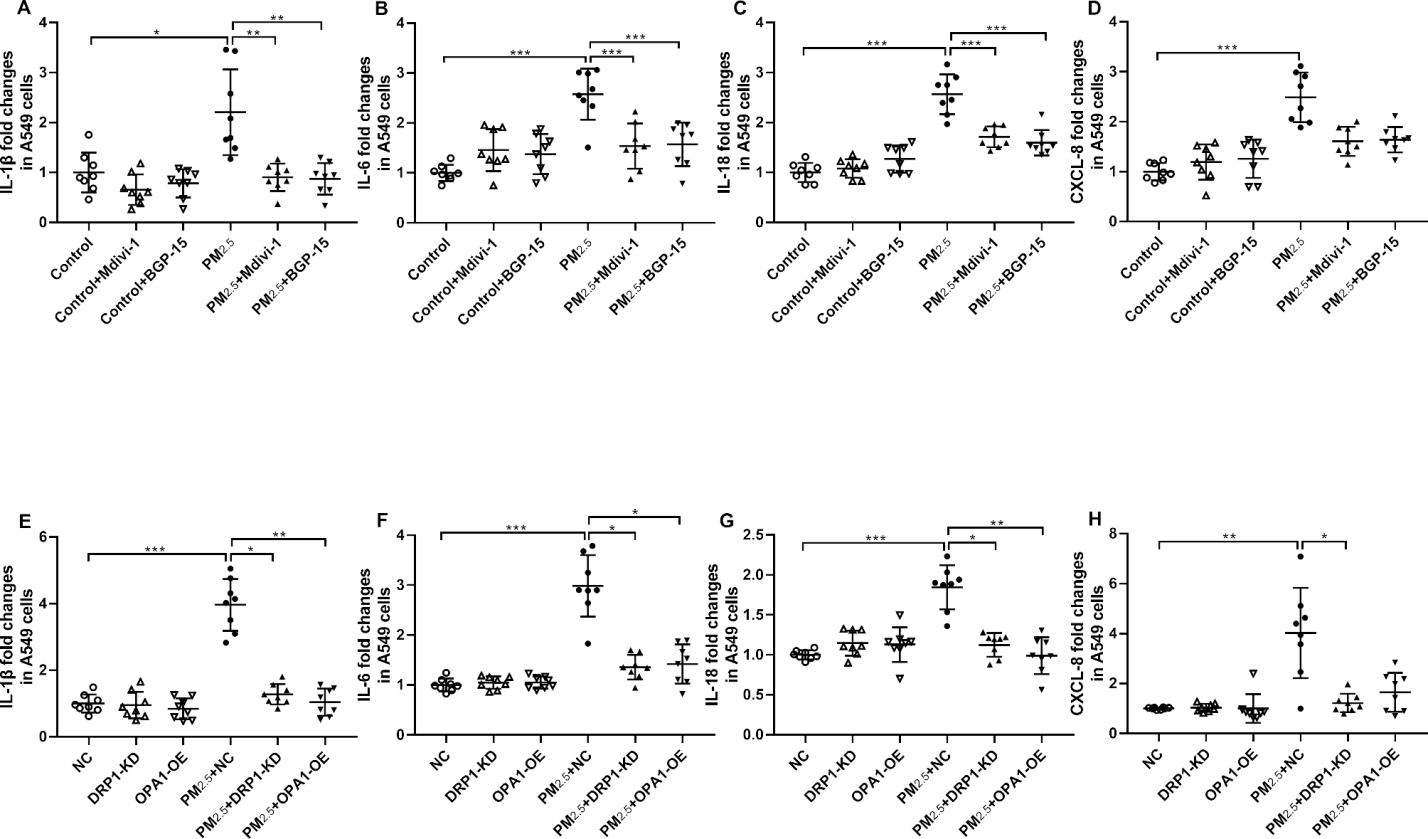
Effect of PM_2.5_, the DRP-1 inhibitor Mdivi-1, the OPA1 activator BGP-15 and of DRP1 knockdown (KD) and OPA1 overexpression (OE) on inflammatory gene expression in A549 cells. Fold changes in IL-1β (**A**), IL-6 (**B**), IL-18 (**C**) and CXCL-8 (**D**) mRNA expression in A549 cells exposed to PM_2.5_ in the presence and absence of Mdivi-1 and BGP-15. Fold changes in IL-1β (**E**), IL-6 (**F**), IL-18 (**G**) and CXCL-8 (**H**) mRNA expression in A549 cells exposed to PM_2.5_ in the presence and absence of DRP1-KD and OPA1-OE. Mean values and data from individual experiments are shown. One-way ANOVA with Bonferroni’s post hoc test (for equal variance) or Dunnett’s T3 post hoc test (for unequal variance) was performed for comparisons among multiple groups. *P < 0.05, **P < 0.01, ***P < 0.001

The cellular release of pro-inflammatory factors such as the cytokines interleukin (IL)-1β, IL-6, IL-8/CXCL8 and IL-18 can be stimulated by PM_2.5_. PM_2.5_ exposure for 48 h significantly increased the mRNA expression of interleukin-1β (IL-1β)(Fig. 2A), IL-6 (Fig. 2B), IL-18 (Fig. 2C) and interluekin-8 (CXCL-8) (Fig. 2D) compared with basal levels in A549 cells. Pretreatment with Mdivi-1 or BGP-15 reduced IL-1β (Fig. 2A), IL-6 (Fig. 2B)and IL-18 (Fig. 2C) mRNA expression. DRP1-KD inhibited IL-1β (Fig. 2E), IL-6 (Fig. 2F), IL-18 (Fig. 2G) and CXCL-8 (Fig. 2H) mRNA, while OPA1-OE inhibited IL-1β (Fig. 2E), IL-6 (Fig. 2F) and IL-18 (Fig. 2G) mRNA.

Caspase-1 protein expression was increased after PM_2.5_ exposure (Figure S4A, 4B). Pretreatment with Mdivi-1 or BGP-15 resulted in a decrease in protein expression of Caspase-1(Figure S4A). Both DRP1-KD and OPA1-OE decreased Caspase-1 protein expression (Figure S4B). Caspase-1, an important pro-inflammatory protease, was activated after PM_2.5_ exposure to process and stimulated a variety of pro-inflammatory factors, such as IL-1β and IL-18. These pro-inflammatory factors are associated with A549 cell damage, which can be prevented by inhibiting mitochondrial fission and promoting mitochondrial fusion.

### Effect of PM_2.5_, Mdivi-1, BGP-15, DRP1-KD and OPA1-OE on mitochondrial morphology and mitochondrial membrane potential

We employed MitoTracker to measure the location, quantity, and morphology of cellular mitochondria, and JC-1 to detect changes in ΔΨm.

**Fig. 3:**
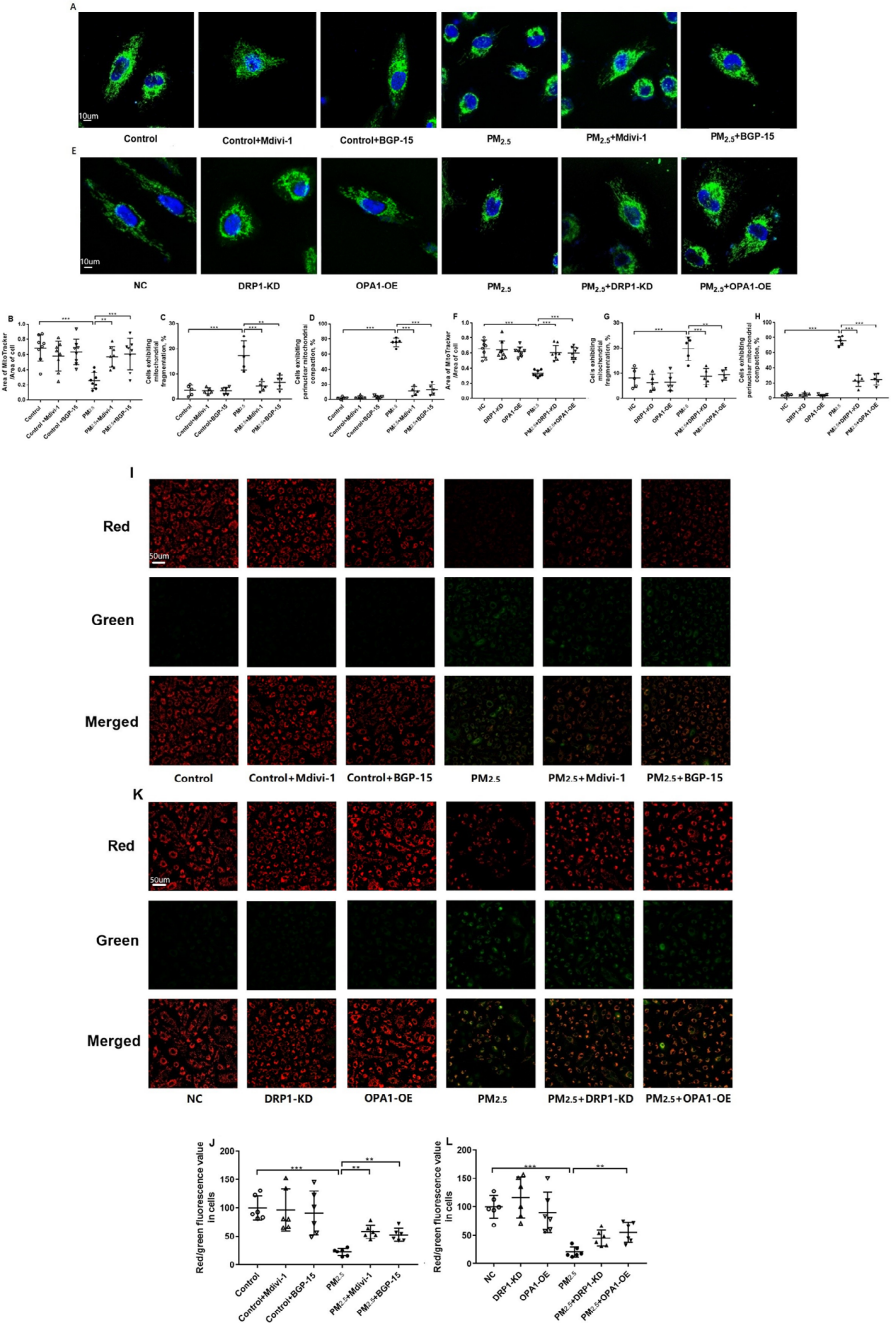
Effect of PM_2.5_, the DRP-1 inhibitor Mdivi-1, the OPA1 activator BGP-15 and of DRP1 knockdown (KD) and OPA1 overexpression (OE) on mitochondrial morphology in A549 cells. Representative microscopy images of the mitochondrial morphology as measured by Mito-Tracker Green FM in PM_2.5_-exposed A549 cells in the presence or absence of the DRP-1 inhibitor Mdivi-1 or the OPA-1 activator BGP-15 in A549 cells are shown (**A, original magnification, x63**). Statistical analysis of the effect of PM_2.5_ exposure in the presence or absence of Mdivi-1 and BGP-15 on mitochondrial area (**B**), the proportion of cells showing mitochondrial fragmentation (**C**) and mitochondrial perinuclear compaction (**D**). Representative microscopy images of the mitochondrial morphology in PM_2.5_-exposed cells in the presence or absence of DRP1 knockdown (KD) or OPA1 overexpression (OE) (**E**). Statistical analysis of the effect of PM_2.5_ exposure in the presence or absence of Mdivi-1 and BGP-15 on mitochondrial area (**F**), the proportion of cells showing mitochondrial fragmentation (**G**) and mitochondrial perinuclear compaction (**H**). Representative microscopy images of the ΔΨm as measured by JC-1 in PM_2.5_-exposed cells pretreated with the Mdivi-1 or BGP-15 (**I, original magnification, x20**) in A549 cells are shown. The graphical results of the mean values from individual experiments plotted as the red/green fluorescence values representing the ΔΨm changes are shown in (**J**). Representative images (**K**) and graphical analysis (**L**) showing the effect of PM_2.5_, DRP1-KD and OPA1-OE on ΔΨm are also shown. One-way ANOVA with Bonferroni’s post hoc test (for equal variance) or Dunnett’s T3 post hoc test (for unequal variance) was performed for comparisons among multiple groups. *P < 0.05, **P < 0.01, ***P < 0.001

Representative images of the effect of PM_2.5_, Mdivi-1 and BGP-15 on mitochondrial morphology in A549 cells are shown in Fig. 3A (original magnification, x63) with statistical analysis of the results provided in Fig. 3B-D. The ratio of mitochondrial area to cell area was significantly decreased by PM_2.5_ exposure (Fig. 3B). Pretreatment with Mdivi-1 orBGP-15 restored normal mitochondrial structure, and increased the ratio of mitochondrial area to cell area (Fig. 3B). PM_2.5_ exposure of A549 cells resulted in 75.6 ± 4.8% of cells exhibiting mitochondrial perinuclear compaction (Fig. 3C) and 17.4 ± 5.1% of cells mitochondrial fragmentation (Fig. 3D). Pretreatment with Mdivi-1 or BGP-15 restored the mitochondrial meshwork and reduced mitochondrial fragmentation (5.2 ± 1.7% and 6.8 ± 2.5% respectively, Fig. 3C) and mitochondrial perinuclear compaction (11.6 ± 5.2% and 13.4 ± 6.2% respectively, Fig. 3D).

Representative images of the effect of PM_2.5_, DRP1-KD and OPA1-OE cells on mitochondrial morphology in A549 cells are shown in Fig. 3E (original magnification, x63) with statistical analysis of the results provided in Fig. 3F-H. The ratio of mitochondrial area to cell area decreased by PM_2.5_ exposure in A549 cells and NC-shRNA cells (Fig. 3F). DRP1-KD and OPA1-OE restored normal mitochondrial structure, and increased the ratio of mitochondrial area to cell area (Fig. 3F). The effects of PM_2.5_ on mitochondrial fragmentation (Fig. 3G) and mitochondrial perinuclear compaction (Fig. 3H) were mimicked by the negative control-short hairpin RNA (NC-shRNA)-treated cells, where 75.8 ± 5.3% of cells exhibited mitochondrial perinuclear compaction and 19.8 ± 4.2% exhibited mitochondrial fragmentation. The proportion of mitochondrial perinuclear compaction was much larger than that of mitochondrial fragmentation after PM_2.5_ exposure. These changes were completely prevented in DRP1-KD and OPA1-OE cells (Fig. 3G, H).

Representative images of ΔΨm identified by red/green fluorescence values are shown in Fig. 3I **and K** (original magnification, x20). Red fluorescence represents normal ΔΨm, while green fluorescence represents decreased ΔΨm and the early stage of apoptosis [22]. The red/green fluorescence values of A549 cells were significantly decreased by PM_2.5_ exposure compared with the control cells and were significantly improved by pretreatment with Mdivi-1 or BGP-15 (Fig. 3I, J). Similar results were seen in control and PM_2.5_-exposed cells in the presence of NC-shRNA cells (Fig. 3K, L). OPA1-OE significantly ameliorated the reduction in red/green fluorescence by PM_2.5_, while DRP1-KD showed a trend towards increasing red/green fluorescence (Fig. 3K, L). Morphological abnormalities of mitochondria and decreased ΔΨm as indicators of mitochondrial damage were present in A549 cells after 48 h of PM_2.5_ exposure. Inhibition of mitochondrial fission and promotion of mitochondrial fusion can partially restore the normal mitochondrial morphology and ΔΨm.

### Effect of PM_2.5_, Mdivi-1, BGP-15, DRP1-KD and OPA1-OE on oxygen consumption rate

We used the Seahorse analyzer to measure cellular oxygen consumption rate (OCR), which is indicative of the basal respiration, maximal respiration, ATP production and spare respiration capacity.

**Fig. 4:**
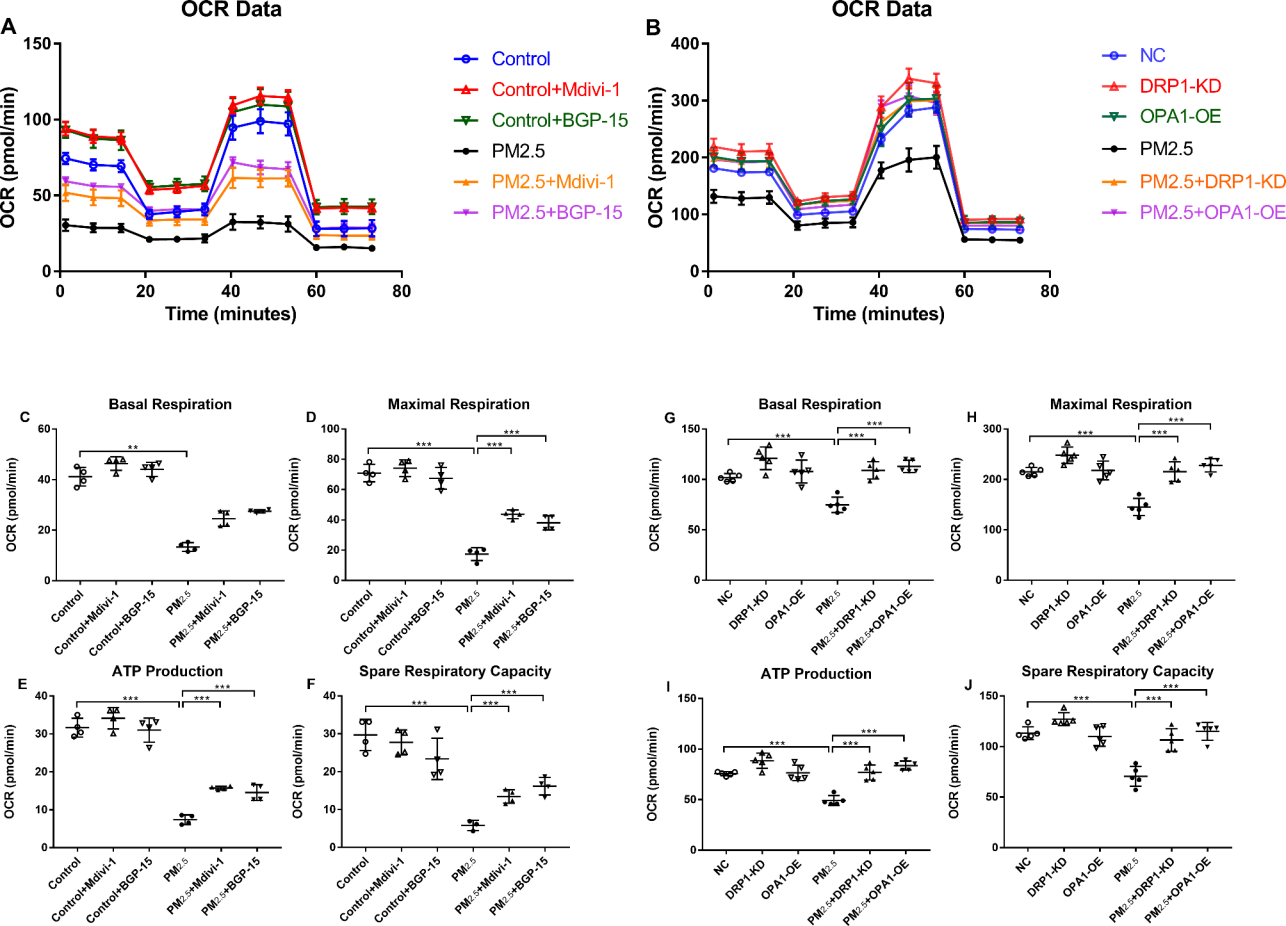
Effect of PM_2.5_, the DRP1 inhibitor Mdivi-1,/ the OPA1 activator BGP-15 and DRP1 knockdown (KD)/ OPA1 overexpression (OE) on mitochondrial function in A549 cells. The effect of Mdivi-1 and BGP-15 on oxygen consumption rate (OCR) in A549 cells (**A**). The effect of DRP1-KD and OPA1-OE on oxygen consumption rate (OCR) in A549 cells (**B**). Graphical presentation of the effect of Mdivi-1 and BGP-15 on PM_2.5_-induced in basal respiration (**C**), maximal respiration (**D**), ATP production (**E**) and spare respiratory capacity (**F**). Graphical analysis of the effect of DRP1-KD and OPA1-OE on PM_2.5_-induced in basal respiration (**G**), maximal respiration (**H**), ATP production (**I**) and spare respiratory capacity (**J**). Mean values and data from individual experiments are shown. One-way ANOVA with Bonferroni’s post hoc test (for equal variance) or Dunnett’s T3 post-hoc test (for unequal variance) was performed for comparisons among multiple groups. *P < 0.05, **P < 0.01, ***P < 0.001

Wild type A549 cells (Fig. 4A) and NC-shRNA cells (Fig. 4B) were exposed to PM_2.5_ for 48 h before measuring the OCR. PM_2.5_ significantly inhibited mitochondrial basal respiration (Fig. 4C, G), maximal respiration (Fig. 4D, H), ATP production (Fig. 4E, I) and spare respiration capacity (Fig. 4F, J) in A549 cells and NC-shRNA cells. Pretreatment with Mdivi-1 and BGP-15 significantly enhanced these cellular respiratory capacities except for basal respiration (Fig. 4C-F). DRP1-KD and OPA1-OE effectively restored cellular respiratory capacities including basal respiration (Fig. 4G), maximal respiration (Fig. 4H), ATP production (Fig. 4I) and spare respiratory capacity (Fig. 4J).

The energy metabolic function of mitochondria in A549 cells was affected after 48 h of PM_2.5_ exposure, leading to a decrease in intracellular ATP synthesis and thus affecting normal cell function. Pretreatment with Mdivi-1 or BGP-15 could restore mitochondrial energy metabolism and maintain normal cellular function. Similar results were seen with DRP1-KD or OPA1-OE.

### Effect of PM_2.5_, Mdivi-1 and BGP-15 on mitochondrial fission and fusion, mitophagy and necroptosis

We examined the levels of mitochondrial fission-related proteins DRP1, p-DRP1, and MFF, fusion-related proteins MFN2 and OPA1, mitophagy-related proteins PINK1, SQSTM1/P62, PARK2, and LC3, as well as necroptosis-related proteins MLKL, RIPK1, and RIPK3.

**Fig. 5:**
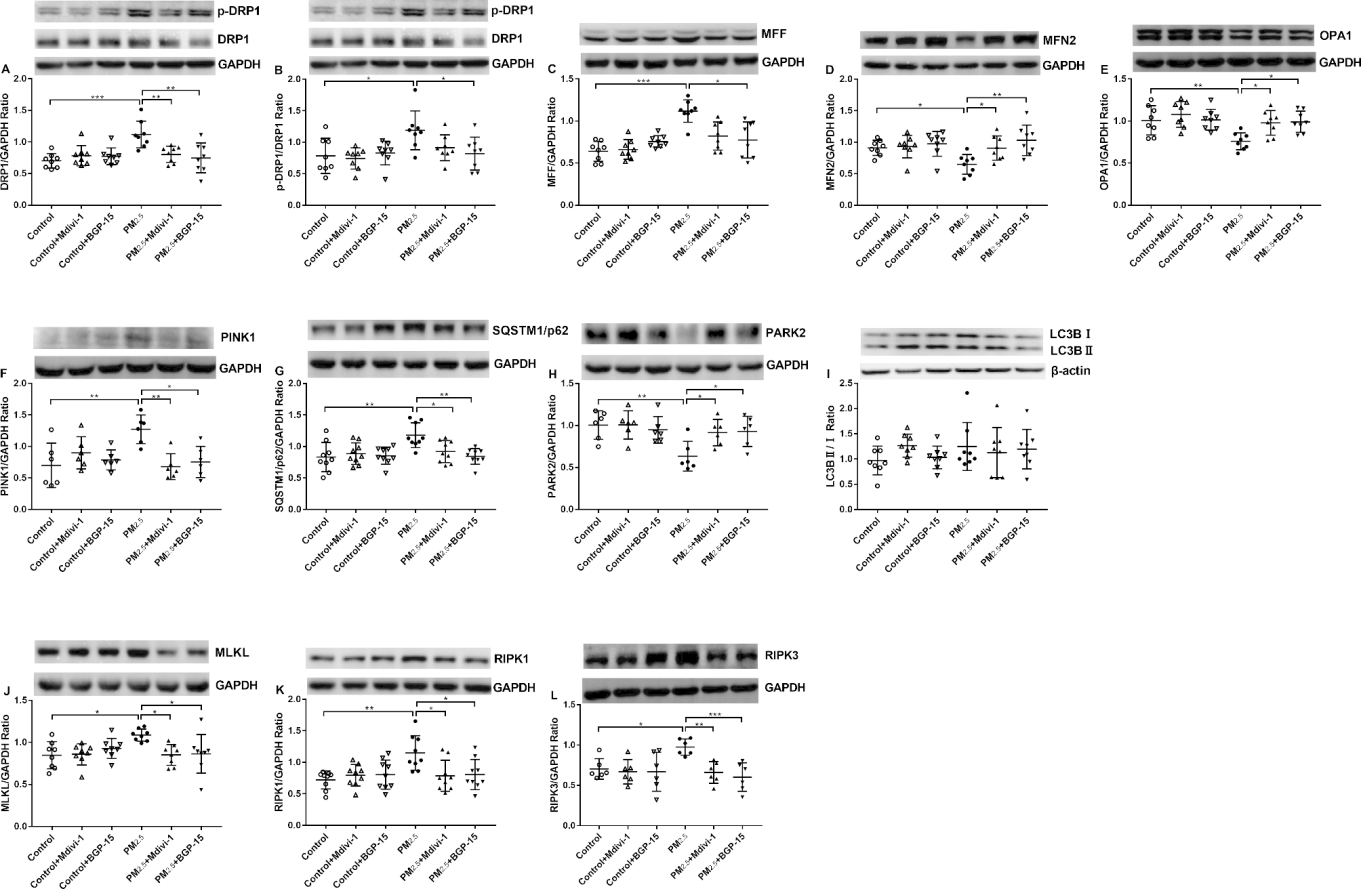
Effect of PM_2.5_, the DRP1 inhibitor Mdivi-1 and the OPA1 activator BGP-15 on mitochondrial fission and fusion proteins, mitophagy- and necroptosis-related proteins in A549 cells. Western blot analysis of the mitochondrial fusion and fission proteins DRP1 (**A**), phosphorylated DRP1 (p-DRP1) (**B**), mitochondrial fission factor (MFF) (**C**), mitofusin2 (MFN2) (**D**) and OPA1 (**E**) after exposure to PM_2.5_ or pretreatment with Mdivi-1/BGP-15. Effect of PM_2.5_, Mdivi-1 and BGP-15 on the expression of the mitophagy-related proteins PTEN induced putative kinase 1 (PINK1) (**F**), sequestosome-1 (SQSTM1/p62) (**G**), recombinant Parkinson disease protein 2 (PARK2) (**H**) and microtubule-associated protein 1 A/1B-light chain 3 (LC3B) (**I**). Effect of PM_2.5_, Mdivi-1 and BGP-15 on the expression of the necroptosis-related proteins mixed lineage kinase domain-like (MLKL)(**J**), receptor-interacting protein kinase 1 (RIPK1) (**K**) and receptor-interacting protein kinase 3(RIPK3) (**L**) Mean values and data from individual experiments are shown. One-way ANOVA with Bonferroni’s post hoc test (for equal variance) or Dunnett’s T3 post hoc test (for unequal variance) was performed for comparisons among multiple groups. *P < 0.05, **P < 0.01, ***P < 0.001

The protein levels of DRP1 (Fig. 5A), p-DRP1/DRP1 ratio (Fig. 5B), and MFF (Fig. 5C) were significantly increased, whilst that of MFN2 (Fig. 5D) and OPA1 (Fig. 5E) were significantly decreased in PM_2.5_ exposed A549 cells compared to controls. Pretreatment with Mdivi-1 significantly reduced DRP1 (Fig. 5A) and increased MFN2 (Fig. 5D) and OPA1 (Fig. 5E) expression. Pretreatment with BGP-15 significantly reduced DRP1 expression (Fig. 5A), p-DRP1/DRP1ratio (Fig. 5B) and MFF (Fig. 5C) expression whilst significantly enhancing MFN2 (Fig. 5D) and OPA1 (Fig. 5E) levels. The expression of mitochondrial fission-related proteins DRP1, p-DRP1, and MFF was elevated after PM2.5 exposure, and the expression of mitochondrial fusion-related proteins OPA1 and MFN2 were decreased, suggesting that mitochondrial fission and fusion disorder was indeed present in PM_2.5_-exposed damaged A549 cells.

The protein expression of PINK1 (Fig. 5F) and SQSTM1/P62 (Fig. 5G) were increased while PARK2 (Fig. 5H) were decreased after 48 h of PM_2.5_ exposure. However, LC3B II/I ratio remained unchanged (Fig. 5I). Pretreatment with Mdivi-1 or BGP-15 inhibited the protein expression of PINK1 (Fig. 5F) and SQSTM1/P62 (Fig. 5G) and increased the protein of PARK2 (Fig. 5H). Combining the results of two aspects of A549 cells after PM_2.5_ exposure, firstly, the mitochondrial morphology mainly presented as perinuclear compaction of mitochondria (a state in which broken mitochondria have been cleared), and secondly, the changes of mitophagy-related proteins PINK1, PARK2, SQSTM1/P62 and LC3. We inferred that mitochondria were in a state of excessive mitochondrial autophagy. Inhibition of mitochondrial fission and promotion of mitochondrial fusion can prevent or inhibit excessive mitochondrial autophagy.

The protein levels of MLKL (Fig. 5J), RIPK1 (Fig. 5K) and RIPK3 (Fig. 5L) were increased in PM_2.5_ exposed A549 cells. Pretreatment with Mdivi-1 or BGP-15 inhibited the expression of MLKL (Fig. 5J), RIPK1 (Fig. 5K) and RIPK3 (Fig. 5L) proteins. Increased expression of necroptosis-related proteins MLKL, RIPK1 and RIPK3, increased mRNA expression of pro-inflammatory factors and inhibition of A549 cell proliferation suggested that the necroptosis pathway was activated in PM_2.5_-exposed A549 cells.

### Effect of PM_2.5_, DRP1-KD and OPA1-OE on mitochondrial fission and fusion, mitophagy and necroptosis

**Fig. 6:**
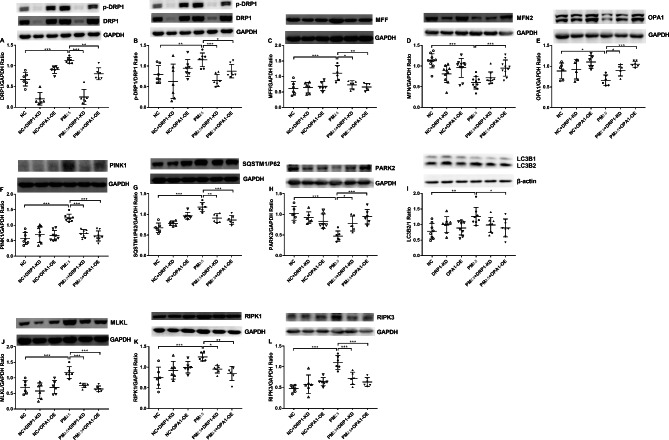
Effect of PM_2.5_, DRP1 knockdown (KD) and OPA1 overexpression (OE) on mitochondrial fission and fusion proteins expression, mitophagy- and necroptosis-related proteins in A549 cells. Western blot analysis of the mitochondrial fusion and fission proteins DRP1 (**A**), phosphorylated DRP1 (p-DRP1) (**B**), mitochondrial fission factor (MFF) (**C**), mitofusin2 (MFN2) (**D**) and OPA1 (**E**) after exposure to PM_2.5_ in DRP1-KD and OPA1-OE cells. Effect of PM_2.5_, DRP1-KD and OPA1-OE on the expression of the mitophagy-related proteins PTEN induced putative kinase 1 (PINK1) (**F**), sequestosome-1 (SQSTM1/p62) (**G**), recombinant Parkinson disease protein 2 (PARK2) (**H**) and microtubule-associated protein 1 A/1B-light chain 3 (LC3B) (**I**). Effect of PM_2.5_, DRP1-KD and OPA1-OE on the expression of the necroptosis-related proteins mixed lineage kinase domain-like (MLKL)(**J**), receptor-interacting protein kinase 1 (RIPK1) (**K**) and receptor-interacting protein kinase 3(RIPK3) (**L**) Mean values and data from individual experiments are shown. One-way ANOVA with Bonferroni’s post hoc test (for equal variance) or Dunnett’s T3 post hoc test (for unequal variance) was performed for comparisons among multiple groups. *P < 0.05, **P < 0.01, ***P < 0.001

The protein levels of DRP1 (Fig. 6A), p-DRP1/DRP1 ratio (Fig. 6B), and MFF (Fig. 6C) were significantly increased, whilst that of MFN2 (Fig. 6D) and OPA1 (Fig. 6E) were significantly decreased in PM_2.5_ exposed cells compared to NC-shRNA cells. In PM_2.5_-exposed cells DRP1-KD and OPA1-OE significantly decreased levels of DRP1 (Fig. 6A), p-DRP1/DRP1 (Fig. 6B) and MFF (Fig. 6C) protein and increased levels of OPA1 protein (Fig. 6E). OPA1-OE cells also showed increased MFN2 protein (Fig. 6D).

Similarly, elevated levels of PINK1 (Fig. 6F) SQSTM1/P62 (Fig. 6G) were observed in PM_2.5_ exposed NC-shRNA cells. The levels of PARK2 declined (Fig. 6H) whilst that of the LC3BII/I ratio (Fig. 6I) also increased significantly. DRP1-KD and OPA1-OE downregulated the protein of PINK1 (Fig. 6F) and SQSTM1/p62 (Fig. 6G) and upregulated expression of PARK2 (Fig. 6H). Moreover, OPA1-OE reduced LC3B II/I ratio (Fig. 6I).

The protein levels of MLKL (Fig. 6J), RIPK1 (Fig. 6K) and RIPK3 (Fig. 6L) were increased in PM_2.5_-exposed NC-shRNA cells. The protein levels of MLKL (Fig. 6J), RIPK1 (Fig. 6K) and RIPK3 (Fig. 6L) were significantly reduced back to control levels in DRP1-KD and OPA1-OE cells.

DRP1-KD and OPA1-OE results were consistent with the Mdivi-1 and BGP-15 groups, and inhibition of mitochondrial fission and promotion of mitochondrial fusion inhibited excessive mitophagy and necroptosis.

### Effect of PM_2.5_, Mdivi-1, BGP-15, DRP1-KD and OPA1-OE on transmission electron microscopy (TEM) analysis

To obtain detailed ultrastructural information of cells, we perform TEM examination.

The cells exposed to PM_2.5_ showed the TEM characteristics of necroptosis, including cell plasma membrane rupture, mitochondrial crista blurred or even disappeared, and nuclear membrane retention (Figure S5A, B). Mitochondrial length was reduced after PM_2.5_ exposure and restored after Mdivi-1 or BGP-15 pretreatment (Figure S5C). This is consistent with our previous findings on mitochondrial fission/fusion. The percentage of abnormal mitochondria increased significantly after PM_2.5_ exposure, and Mdivi-1 or BGP-15 pretreatment reduced the percentage of abnormal mitochondria (Figure S5D). The results of DRP1-KD/OPA1-OE group were similar to those of the pharmacological group (Figure S5E, F). Based on the necroptosis cellular changes we observed in TEM and changes in necroptosis-related proteins MLKL, RIPK1 and RIPK3, we inferred that necroptosis of A549 cells was present after PM2.5 exposure was under control after inhibition of mitochondrial fission and promotion of mitochondrial fusion.

### Effect of PM_2.5_, Mdivi-1 and BGP-15 on AHR

**Fig. 7:**
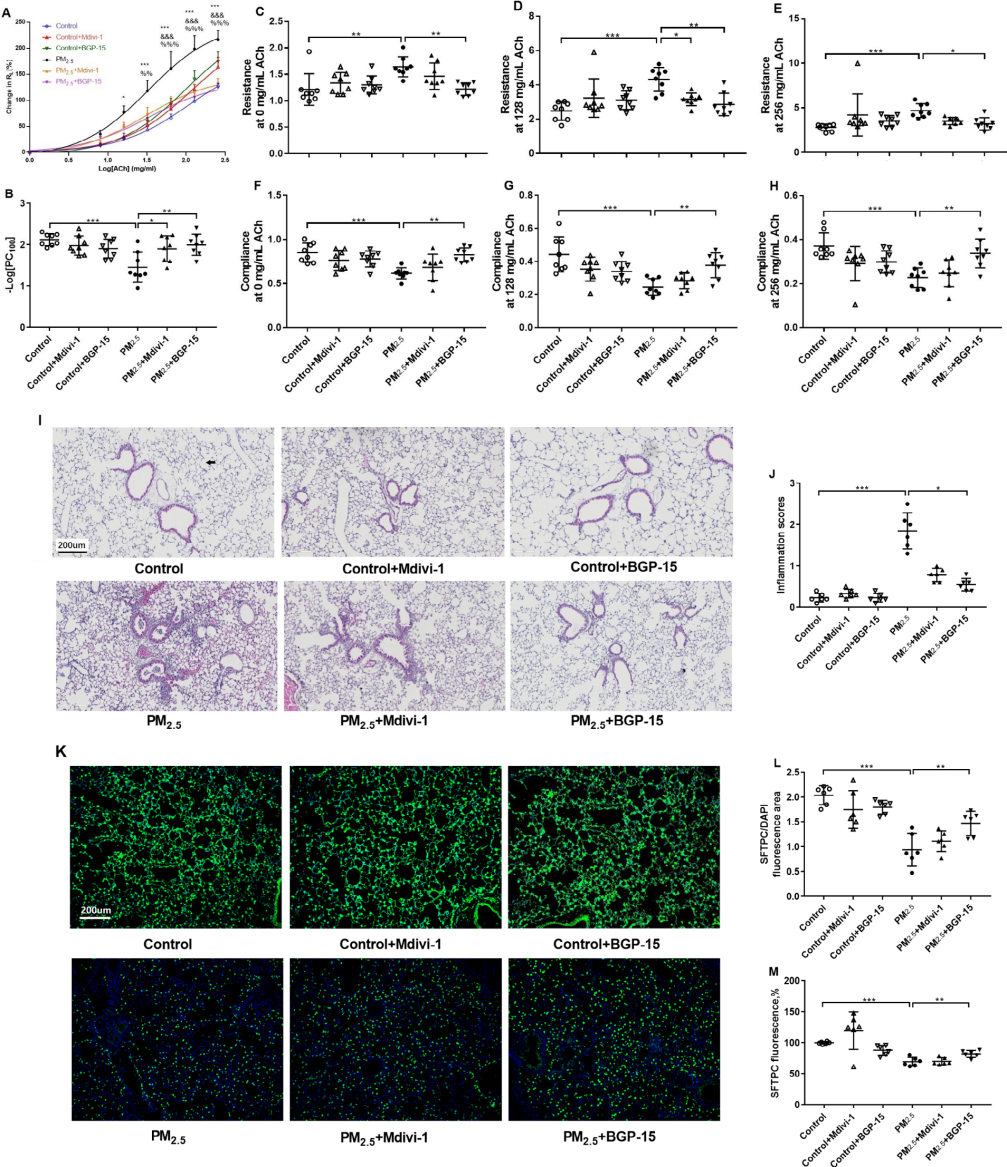
Effect of PM_2.5_, the DRP1 inhibitor Mdivi-1 and the OPA1 activator BGP-15 on airway hyper-responsiveness (AHR) in mice. Mean percentage increase in lung resistance (RL) to increasing concentrations of acetylcholine (**A**). *P < 0.05, **P < 0.01, and ***P < 0.001 compared with control mice, &P < 0.05, &&P < 0.01, and &&&P < 0.001 compared with the DRP1 inhibitor (Mdivi-1) + PM_2.5_ mice, %P < 0.05, %%P < 0.01, and %%%P < 0.001 compared with the OPA-1 activator (BGP-15) + PM_2.5_ mice. Individual and mean –logPC100, an indicator of bronchial responsiveness. n = 8 in each group (**B**). Individual and mean airway resistance (**C-E**) or compliance (**F-H**) at 0 (**C, F**), 128 (**D, G**) and 256 mg/L (**E, H**) of acetylcholine. Representative photomicrographs of mouse lung tissues in hematoxylin and eosin (H&E)-stained sections from control distilled water instilled mice, PM_2.5_-instilled mice, Mdivi-1-pretreated PM_2.5_-instilled mice and BGP-15-pretreated PM_2.5_-instilled mice **(I, original magnification, x10)**. Individual and mean values of inflammation scores measured from H&E-stained sections (**J**). Representative pictures of SFTPC immunofluorescence staining in the lung tissues of PM_2.5_, Mdivi-1- and BGP-15-treated cells are shown **(K, original magnification, x10)**. Graphical analysis of SFTPC/DAPI fluorescence area (**L**) and SFTPC fluorescence (**M**). One-way ANOVA with Bonferroni’s post hoc test (for equal variance) or Dunnett’s T3 post hoc test (for unequal variance) was performed for comparisons among multiple groups. *P < 0.05, **P < 0.01, ***P < 0.001

AHR, indicated as lung compliance and resistance towards different concentrations of ACh, and lung inflammtion are two common pathophysiological characteristics of PM_2.5_ exposure of animals.Compared to distilled water-instilled mice, PM_2.5_-instilled mice demonstrated a significant leftward shift of the concentration-response curve at 16 to 256 mg/mL of ACh (Fig. 7A) with decreased -logPC_100_ (minimum concentration of acetylcholine required to cause a 100% increase in airway resistance, 2.12 ± 0.14 vs. 1.45 ± 0.36, Fig. 7B) indicating an increase in airway responsiveness to the ACh challenge and increased lung resistance. In PM_2.5_-instilled mice, pretreatment with Mdivi-1 or BGP-15 reduced airway responsiveness in terms of -logPC_100_ compared to that in distilled water-pretreated mice (1.90 ± 0.31 vs. 1.45 ± 0.36, 2.00 ± 0.25 vs. 1.45 ± 0.36). Pretreatment with Mdivi-1 inhibited RL at 64, 128 and 256 mg/mL of ACh (Fig. 7A), and pretreatment with BGP-15 inhibited RL at 32, 64, 128 and 256 mg/mL of ACh (Fig. 7A).

PM_2.5_-instilled mice demonstrated a significant increased lung resistance(Fig. 7C-E) and decreased lung compliance (Fig. 7F-H) at 0 (Fig. 7C, F), 128 (Fig. 7D, G) and 256 mg/L (Fig. 7E, H) of ACh compared to distilled water-instilled mice. In PM_2.5_-instilled mice, pretreatment with BGP-15 reduced lung resistance (Fig. 7C-E) and restored lung compliance (Fig. 7F-H) at 0, 128 and 256 mg/L of ACh compared to that in PM_2.5_-instilled mice, and pretreatment with Mdivi-1 exhibited a reduction in pulmonary resistance in mice only at 128 mg/L of ACh (Fig. 7D).

### Effect of PM_2.5_, Mdivi-1 and BGP-15 on lung histology and immunofluorescence

The extent of lung inflammation was evaluated in the H&E-stained lung sections. Assessment of Type II alveolar epithelial cell (AECII) injury was performed by SFTPC immunofluorescence assay.

Representative images of the lung tissue with infiltration of inflammatory cells around the bronchus and vessel after instillation of PM_2.5_ are shown in Fig. 7I (original magnification, x10). There were higher inflammation scores in PM_2.5_-instilled mice compared with distilled water-instilled mice (Fig. 7J). Pretreatment with BGP-15, but not Mdivi-1, significantly reduced inflammation scores in PM_2.5_-instilled mice compared with distilled water-pretreated mice (Fig. 7J). Type II alveolar epithelial cells (AECII) are the stem cell in the newborn lung that can effectively differentiate into Type I alveolar epithelial cells (AECI), which constitute the alveolar structure involved in pulmonary respiratory function [23]. SFTPC protein is the characteristic protein expressed in AECII. Representative images of SFTPC in lung alveolar space are shown in Fig. 7K (original magnification, x10). SFTPC/DAPI fluorescence area (Fig. 7L) and SFTPC fluorescence intensity (Fig. 7M) are used to represent the number an integrity of AECII. SFPTC expression was decreased in PM_2.5_-instilled mice compared with distilled water-instilled mice, while pretreatment with BGP-15, but not Mdivi-1, restored PM_2.5_-induced decrease in SFTPC expression (Fig. 7L, M). SFTPC immunofluorescence was restored and lung alveolar cell injury was attenuated in BGP-15 pretreated mice, but not in Mdivi-1 pretreated mice.

### Effect of PM_2.5_, Mdivi-1 and BGP-15 on lung mitochondrial dynamics and necroptosis

**Fig. 8:**
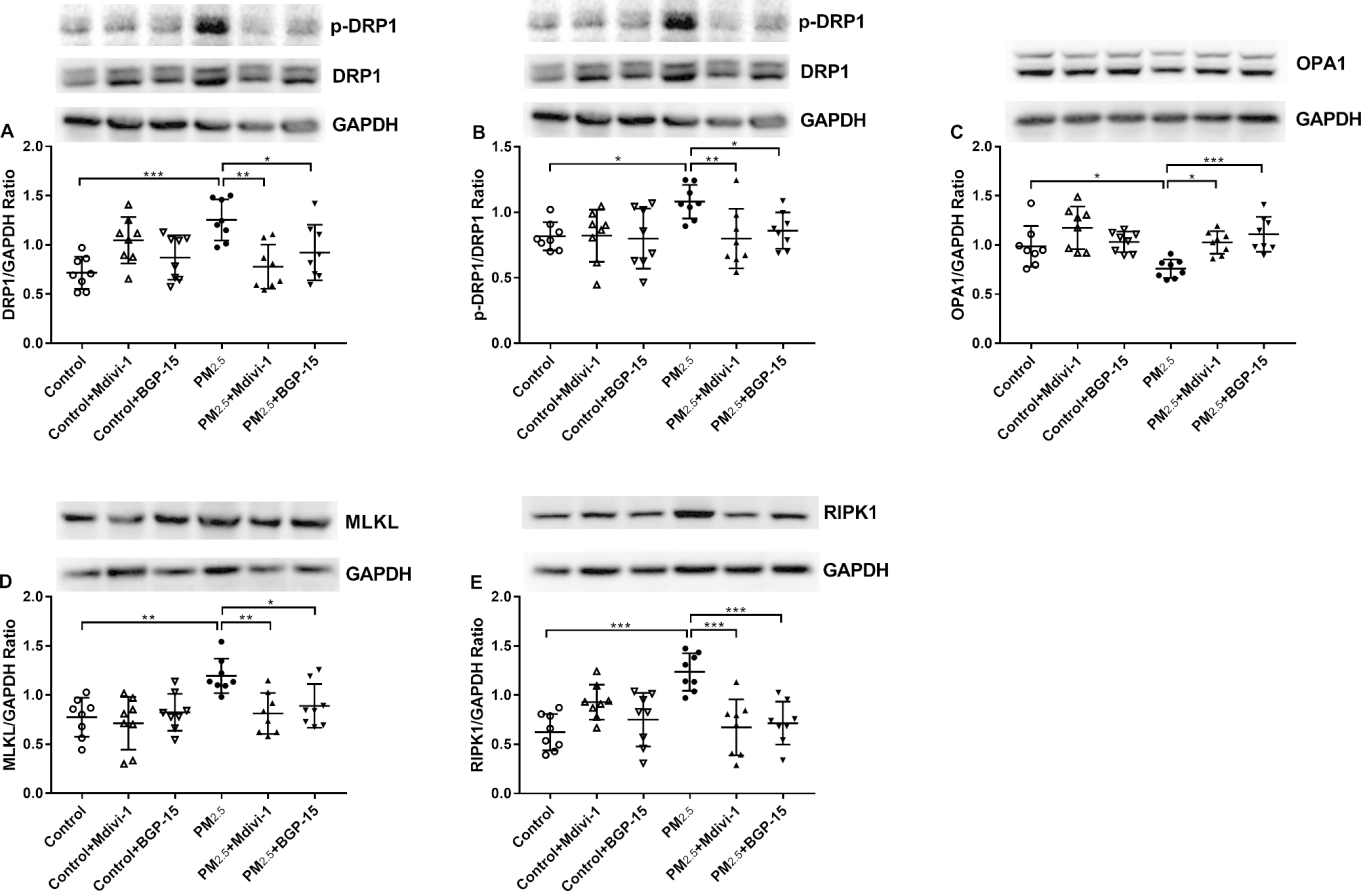
Effect of PM_2.5_, the DRP1 inhibitor Mdivi-1 and the OPA1 activator BGP-15 on mitochondrial fission and fusion proteins and necroptosis-related proteins in lungs in mice. Effect of PM_2.5_, Mdivi-1 and BGP-15 on Western blot analysis of the mitochondrial fusion and fission proteins DRP-1 (**A**), phosphorylated DRP-1 (p-DRP1) (**B**) and OPA1 (**C**) after exposure to PM_2.5_. Effect of PM_2.5_ and Mdivi-1/BGP-15 on necroptosis related proteins expression in mice. Western blot analysis of the necroptosis related proteins mixed lineage kinase domain-like (MLKL) (**D**) and receptor-interacting protein kinase 1 (RIPK1) (**E**) after exposure to PM_2.5_ or pretreatment with Mdivi-1 or BGP-15. Mean values and data from individual experiments are shown. One-way ANOVA with Bonferroni’s post hoc test (for equal variance) or Dunnett’s T3 post-hoc test (for unequal variance) was performed for comparisons among multiple groups. *P < 0.05, **P < 0.01, ***P < 0.001

There was increased expression of the mitochondrial fission proteins DRP1 (Fig. 8A) and of the p-DRP1/DRP1 ratio (Fig. 8B) together with significantly reduced OPA1 expression (Fig. 8C) in lungs exposed to PM_2.5_. Pretreatment with Mdivi-1 and BGP-15 significantly attenuated PM_2.5_-induced changes in DRP1 (Fig. 8A), p-DRP1/DRP1 (Fig. 8B) and OPA1 (Fig. 8C) levels.

The necroptosis-related protein expression of MLKL and RIPK1, was increased in lung tissues of PM_2.5_-instilled mice and decreased in Mdivi-1 or BGP-15 pretreated mice (Fig. 8D, E). Combining the results of in vivo experiments and in vitro experiments, the necroptosis pathway was activated in PM_2.5_-induced damage to A549 cells as well as in lung tissue, and inhibition of mitochondrial fission and promotion of mitochondrial fusion prevented the activation of the necroptosis pathway.

## Discussion

In the present study, we confirmed the damaging effects of PM_2.5_ in alveolar epithelial cells and murine lung tissue. Co-culture with PM_2.5_, A549 cells exhibited pro-inflammatory responses, redox-responses, abnormal mitochondrial function and structure, increased mitochondrial fission with reduced mitochondrial fusion, and enhanced mitophagy and necroptosis. PM_2.5_ intranasal instillation induced lung inflammation, AHR, disturbed mitochondrial dynamics, enhanced necroptosis and type II alveolar epithelial cell (AECII) injury. Mdivi-1 or BGP-15 and DRP1-KD or OPA1-OE alleviated the damage and abnormal mitochondrial function in PM_2.5_ exposed A549 cells by inhibiting the pro-inflammatory response, redox-responses and necroptosis, regulating mitochondrial dynamics and mitophagy. Similar results were found in murine lung tissue, where Mdivi-1or BGP-15 demonstrated protective effects by regulating mitochondrial dynamics, necropotosis, and pro-inflammatory responses.

Biochemical analyses of PM_2.5_ have been reported previously [7]. Organic carbon (OC) may play an important role in lung epithelial damage by PM_2.5_. A recent study in Shanghai suggested that PM_2.5_ constituents from the combustion of fossil fuel (e.g., OC and EC) may have an appreciable influence on the health impact attributable to PM_2.5_. OC concentrations correlated significantly with adverse health effects, such as cardiopulmonary diseases, which require emergency hospitalization [24, 25]. In addition, organic extracts of PM_2.5_ collected in Seoul induced neutrophilic inflammation, cellular aging and enhanced macro autophagy in primary lung epithelial cells [26]. The biochemical effects of PM_2.5_ rely on its multiple components and this is similarly likely for its adverse health effects which may vary depending on its chemical characteristics and where it is generated.

PM_2.5_ can induce pro-inflammatory responses and redox-responses in cells and in the lung. For example, PM_2.5_ can increase the expression of pro-inflammatory cytokines and their receptors [8]. Our experimental results are consistent with previous studies in that IL-1, IL-6, IL-18 and CXCL-8 mRNA expression was increased in A549 cells after PM_2.5_ exposure, and inhibition of mitochondrial fission and promotion of mitochondrial fusion reduced the pro-inflammatory response caused by PM_2.5_. Short-term exposure of PM_2.5_ to mice induced inflammatory cell infiltration and lung tissue congestion, increasing the expression of pro-inflammatory mediators such as TNF-α, IL-6 and IL-1β, and oncogenes such as c-Fos and c-Jun [27]. AHR considered as a hallmark of allergic asthma, may also be evoked by neutrophilia and increases in pro-inflammatory cytokines such as TNF-α and IL-1β [28, 29]. PM_2.5_ exposure aggravates AHR and allergic airway responses [30, 31] similar to the results shown in our study. PM_2.5_-induced AHR in mice represents, to some extent, an enhanced pro-inflammatory response and oxidative stress in mice in the respiratory tract, leading to a narrowing of the airways and the stiffening of the alveoli and lung tissue. Administration of Mdivi-1or BGP-15 significantly inhibited PM_2.5_-induced lung inflammatory cell infiltration and prevented PM_2.5_-induced AHR. These results demonstrate that imbalance of mitochondria fission and fusion may be needed for the expression of pro-inflammatory mediators.

PM_2.5_-induced redox-responses, characterized by an altered redox regulation with increased ROS, could lead to disruption of redox homeostasis and sustained cell or tissue damage [5, 32]. Regulation of mitochondrial dynamics could protect the cells from redox-responses. In vivo and in vitro studies revealed that Mdivi-1 or BGP-15 treatment can significantly reduce lipopolysaccharide (LPS)-induced oxidative stress [15, 33, 34]. Furthermore, microglial cells deficient in mitochondrial fission were protected from LPS-mediated ROS production and pro-inflammatory mediator production [35]. Extramitochondrial ROS can amplify mitochondrial ROS production leading to mitochondrial damage and cell death [36]. Alterations in mitochondrial ROS induced by stress can affect mitochondrial quality control mechanisms in various tissues leading to activation of inflammatory pathways that may influence pathological processes [37]. In our study, PM_2.5_-stimulated cells produced more intracellular and mitochondrial ROS, while Mdivi-1, BGP-15, DRP1-KD and OPA1-OE reduced this cellular redox-responses.

Mitochondria adapt to homeostatic perturbations induced by cellular stress. This includes changes in biogenesis or remodeling of the mitochondrial network by fission or fusion events and elimination of damaged mitochondria by mitophagy [38]. Mitophagy involves mitochondrial fission proteins such as DRP1 [39] and mild oxidative stress specifically triggers mitophagy in a DRP1-dependent manner. PINK1 stabilization on the OMM recruits the E3 ligase PARK2. The E3 ligase Gp78 which is associated with smooth endoplasmic reticulum membranes is activated independently of PARK2 to promote fission. Both PARK2 and Gp78 potentiates the degradation of MFN 1/2. AMPK activates MFF to recruit DRP1 which mediates membrane “constriction” at the site of fission through its GTPase activity. The scaffolding protein SQSTM1/p62 binds to OMM ubiquitin in addition to Smurf1 and Nix, to facilitate mitochondria targeting to LC3 coated autophagosomes [39]. Our results confirmed that mitophagy, combined with altered mitochondrial dynamics, controls mitochondrial quality. Based on changes in the mitochondrial morphology (mainly presented as perinuclear compaction of mitochondria) and changes of the mitophagy-related proteins (PINK1, PARK2, SQSTM1/P62 and LC3) after PM_2.5_ exposure, we inferred that mitochondria were in a state of excessive mitophagy. Inhibition of mitochondrial fission and promotion of mitochondrial fusion can inhibit excessive mitophagy.

The enhanced mitochondrial fission and diminished mitochondrial fusion by PM_2.5_ were accompanied by PINK1-dependent mitophagy. In our study, PARK2 decreased after PM_2.5_ exposure confirming previous results [19, 40]. However, the redundancy of PARK2 in regulating autophagy and the conflicting reports on its role in the fission and fusion processes suggest that PARK2 does not play a leading role in mitophagy [39]. Here, we have shown that in PM_2.5_-exposed alveolar epithelial cells, mitochondrial dysfunction was associated with mitochondrial fission and fusion as well as mitophagy, and that this process depended on the stability of PINK1. By inhibiting mitochondrial fission and promoting fusion, PINK1-dependent mitophagy was surpressed and mitochondrial respiratory functions such as basal respiration, maximal respiration, ATP production, and spare respiratory capacity were restored.

Changes in mitochondrial dynamics observed in response to PM_2.5_ suggest that the total mitochondrial content of a cell is in a dynamic equilibrium between mitochondrial biogenesis and mitochondrial degradation, including mitophagy and other forms of mitochondrial recycling [41]. In general, more-networked mitochondria are more efficient at generating ATP, particularly by aerobic metabolism, and low mitochondrial mass impedes production of a more-networked morphology, which is consistent with our findings [42, 43]. Different levels, duration and timing of stimuli can have varying impacts on mitochondrial morphology. Low level chronic exposure increases fusion and gives a mild increase in fission, resulting in more-networked mitochondria as well as increased turnover of damaged components [44].This may also be an adaptive mechanism to avoid cell death.

Fusion can also be beneficial by permitting “functional complementation”: if specific mitochondria carry a high level of damaged components or mutated mitochondrial DNA (mtDNA), the deleterious effects of these dysfunctional components may be compensated for by functional components from other mitochondria [45]. Fission may allow identification of dysfunctional daughter mitochondria and their subsequent removal via lysosomal degradation. Acute exposure to high levels of stimuli promotes fission and may simultaneously inhibit fusion due to reduced ΔΨm. High level exposure may also result in enhanced fission, leading to excessive mitophagy, blocks efficient ATP generation and limits rates of functional complementation, in addition to potentially leading to apoptosis [41]. Diminished mitochondrial fusion results in reduced endoplasmic reticulum and lysosomal membrane fusion and phagocytosed and/or damaged mitochondria cannot be removed whilst dysfunctional mitochondria carrying large amounts of mitochondrial DAMPs cannot be compensated for [46]. In our study, an imbalance of mitochondrial fission and fusion caused by high level acute exposure to PM_2.5_ emphasizes the effect on mitochondrial and cellular damage.

The increased expression of necroptosis-related proteins MLKL and RIPK1 observed following PM_2.5_ exposure in A549 cells and mice, increased mRNA expression of pro-inflammatory factors and inhibition of A549 cell proliferation suggested that the necroptosis pathway was activated after PM_2.5_ exposure. Necroptosis in A549 cells and murine lung was controlled after inhibition of mitochondrial fission and promotion of mitochondrial fusion. Previous studies have also suggested that Beas2B cells undergo mitophagy-dependent necroptosis mediated by PINK1-induced mitophagy, and that Mdivi-1 protected against CS-induced cell death and mitochondrial dysfunction in vitro by reducing the phosphorylation of MLKL [19]. These data suggest that PM_2.5_-induced changes in molecules related to mitochondrial dynamics can regulate the necrosome.

AECII in lung tissues play an important role in timely repair of damaged cells to prevent invasion by external pathogens, while SFTPC, surfactant-associated protein A (SFTPA) and surfactant-associated protein D (SFTPD) proteins can improve lung compliance and play an immunomodulatory role [47, 48]. The number and size of mitochondria are reduced in AECII when they differentiate to AECI to repair or replace the lung epithelium after damage. Data on lung resistance and lung compliance in mice found that elevated lung resistance and decreased compliance in mice due to PM_2.5_ could be restored by modulating mitochondrial fission and fusion. Pulmonary resistance and compliance are used as a measure of pulmonary edema and an indication of AECII impairment. Our results suggested that BGP-15 reduced the AECII apoptosis under PM_2.5_ exposure. PM_2.5_-induced apoptosis of AECII was accompanied by increased expression of MLKL, RIPK1 and RIPK3, so we assume that necropotosis plays an important role in AECII apoptosis, which can be controlled by modulation of mitochondrial fusion.

There are several limitations in the present research. First, as our results show, there are some differences between the effects of drugs versus KD/OE in cells and in vivo. We consider that it may be due to the existence of other compensation mechanisms. Second, due to the limitations of experimental conditions, we used A549 cells as a model of primary AECII in this study. Third, to better stimulate the physiological state of lung cells in vivo, 3D air-liquid interface (ALI) culture can be applied in later studies. Fourth, increased cell permeability[49–51] and impaired intercellular barrier function are induced by exposure to PM_2.5_[52, 53], which are not examined in the current study and can be resolved in the future. Fifth, we did not monitor the tightness of the cell monolayer by measuring transepithelial electrical resistance (TEER), which is under our planning. Although we did not formally assess whether the PM_2.5_ effects were due to the presence of endotoxin in our experiments, the low levels presence (0.68EU/ml) suggest that this is unlikely. Therefore, our experimental results may only be applicable to the PM_2.5_ exposure of A549 cells under submerged conditions at a single time point.

## Conclusions

In summary, we have demonstrated that an imbalance of mitochondrial fission and fusion plays an important role in PM_2.5_-induced cells and lung injury. Inhibition of mitochondrial fission and promotion of mitochondrial fusion can restore PM_2.5_-induced cellular and lung damage by suppressing pro-inflammatory responses and ROS responses, regulating the expression of mitophagy-related proteins (e.g., PINK1, PARK2), and decreasing the expression of necroptosis-related proteins (e.g., MLKL, RIPK1). Future studies should examine these effects in primary cells at various time points after PM_2.5_ exposure.

## Electronic supplementary material

Below is the link to the electronic supplementary material.

Supplementary Material 1

Supplementary Material 2

## Data Availability

The datasets during and/or analyzed during the current study are available from the corresponding author on reasonable request.

## Abbreviations

Ach: Acetylcholine
AECI: Type I alveolar epithelial cell
AECII: Type II alveolar epithelial cell
AHR: Airway hyperresponsiveness
ATP: Adenosine-triphosphate
BCA: Bicinchoninic Acid
CCK8: Cell counting kit-8
CSE: Cigarette smoke extract
ΔΨm: Mitochondrial membrane potential
DAMPs: Damage-associated molecular patterns
DAPI: 4',6-diamidino-2-phenylindole
DCFH-DA: 2',7'-Dichlorodihydrofluorescein diacetate
DMSO: Dimethyl sulfoxide
DRP1: Dynamin-related protein 1
ETC: Electron transfer chain
Fis1: Fission 1
H&E: Hematoxylin and eosin
HRP: Horse radish peroxidase
IL: Inflammatory cytokines
IMM: Inner mitochondrial membranes
KD: Knockdown
OE: Overexpressing
LC3: 1A/1B-light chain 3
LPS: Lipopolysaccharide
MFF: Mitochondria fission factor
MFN1: Mitofusin 1
MFN2: Mitofusin 2
MID49: Mitochondrial dynamics protein of 49 kDa
MID51: Mitochondrial dynamics protein of 51 kDa
MLKL: Mixed-lineage kinase domain-like protein
mtDNA: Mitochondrial DNA
mtROS: Mitochondrial ROS
NC-shRNA: Negative control-short hairpin RNA
Nrf2: NF-E2-related factor-2
OC: Organic carbon
OCR: Mitochondrial oxygen consumption rate
OMM: Outer mitochondrial membranes
OPA1: Optic atrophy 1
PAHs: Polycyclic aromatic hydrocarbons
PARK2: Parkinson Disease protein 2
PBS: Phosphate buffer solution
PCR: Polymerase chain reaction
p-DRP1: Phosphorylated DRP1
PEG 300: Polyethylene glycol 300
PINK1: PTEN induced putative kinase 1
PM_2.5_: Particulate matter with an aerodynamic diameter less than 2.5µm
PVDF: Polyvinylidene fluoride
RIPK: Receptor-interacting protein kinase
RL: Lung resistance
ROS: Reactive oxygen species
SDS-PAGE: Sodium dodecyl sulfate polyacrylamide gel electrophoresis
SFTPA: Surfactant-associated protein A
SFTPC: Surfactant-associated protein C
SFTPD: Surfactant-associated protein D
SQSTM1/p62: Sequestosome-1
TOC: Total organic carbon
