# Supplementary material for: Attenuation of PM2.5-induced alveolar epithelial cells and lung injury through regulation of mitochondrial fission and fusion
Source: Part Fibre Toxicol. 2023 Jul 18;20:28. doi: 10.1186/s12989-023-00534-w (PMC10353144; doi:10.1186/s12989-023-00534-w)
Supplement: Supplementary file 1 — Supplementary Material 1 [file 12989_2023_534_MOESM1_ESM.docx]

**Figure1**

DRP1
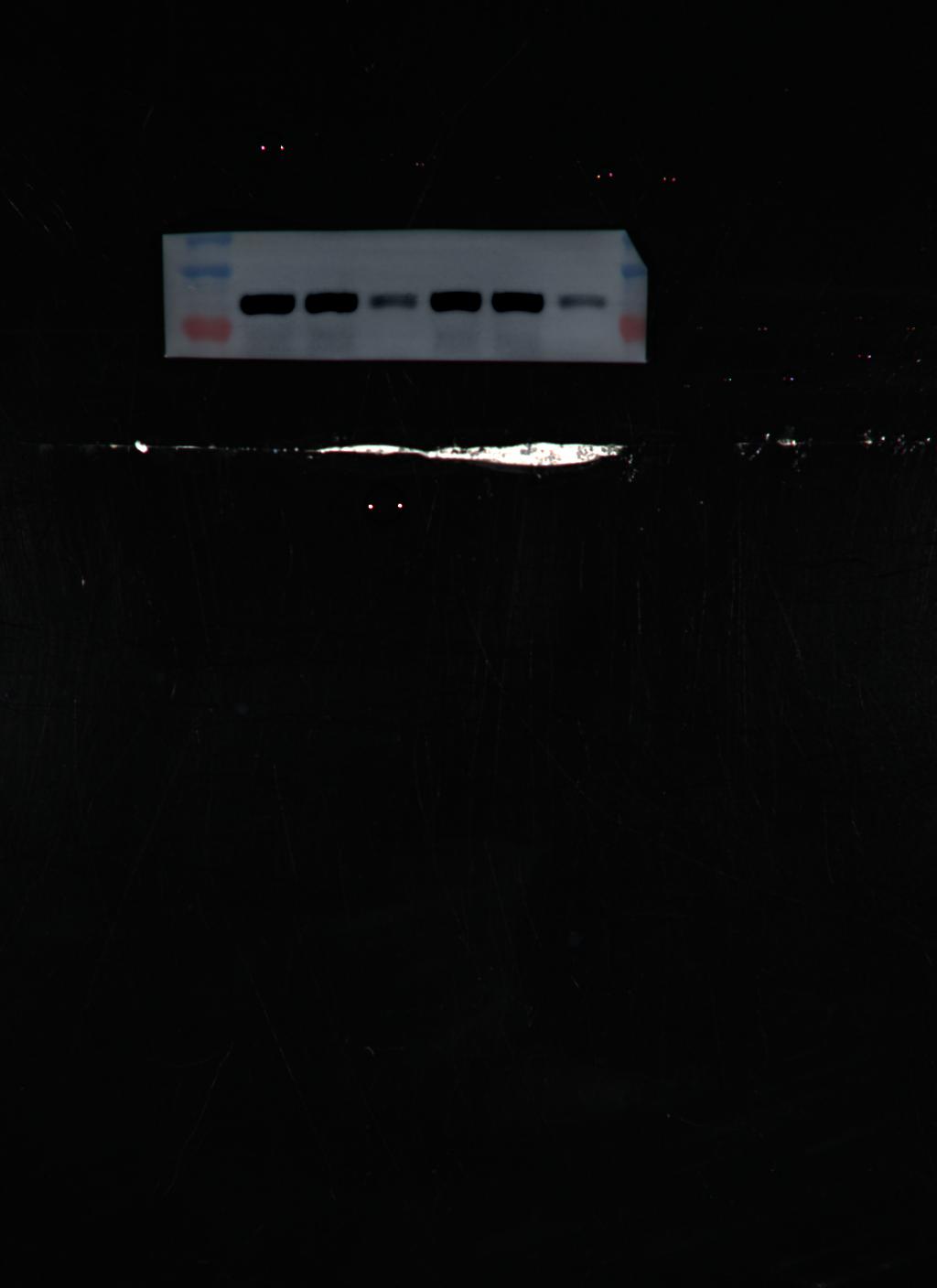


GAPDH
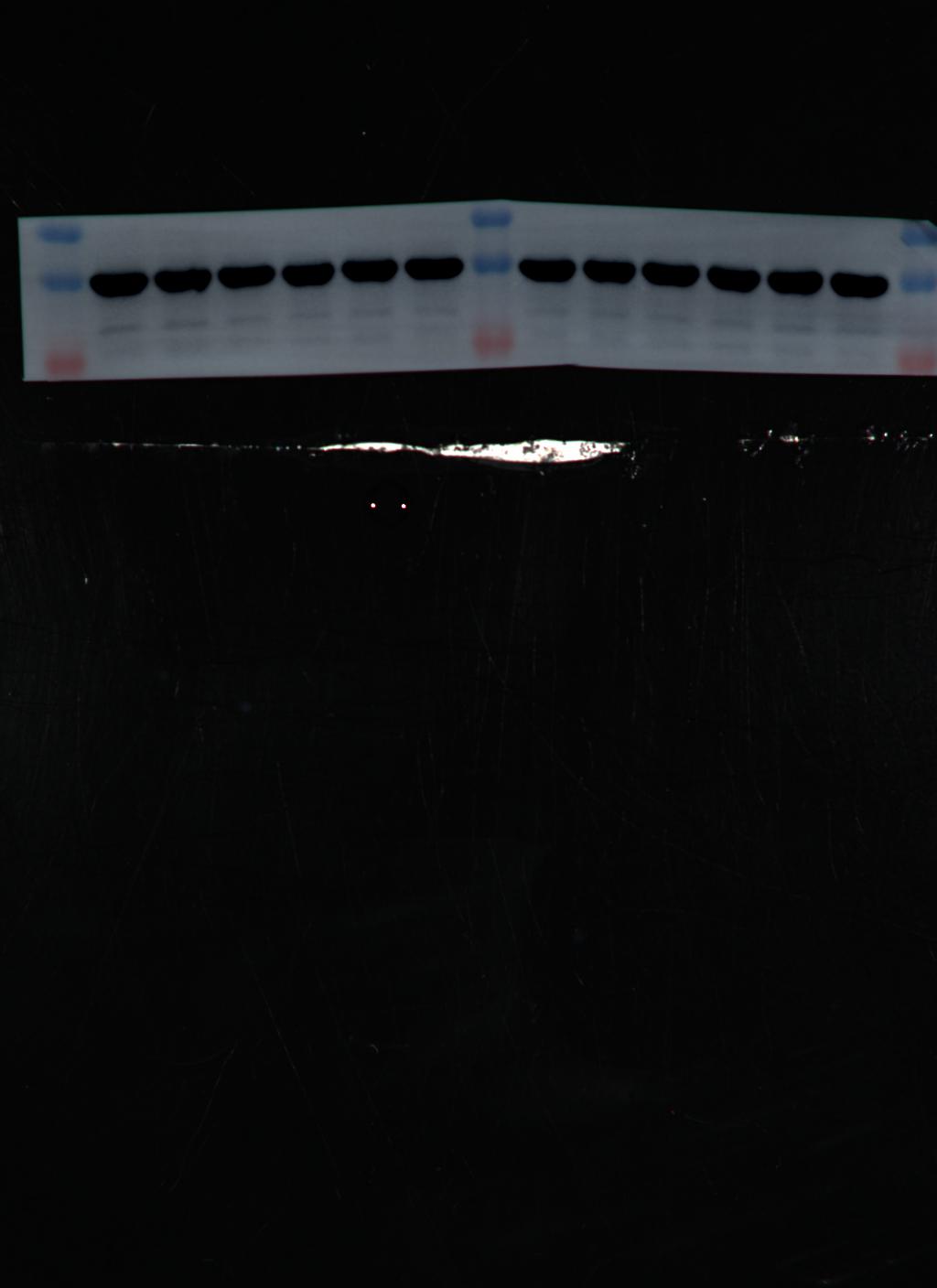


OPA1
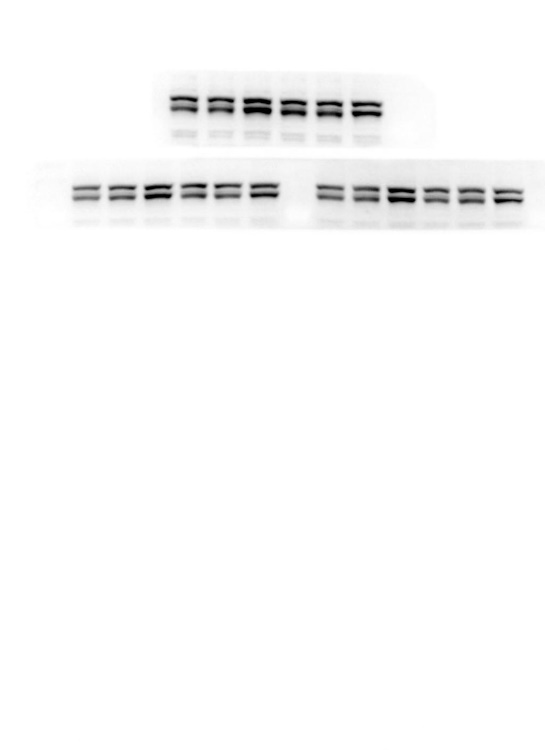

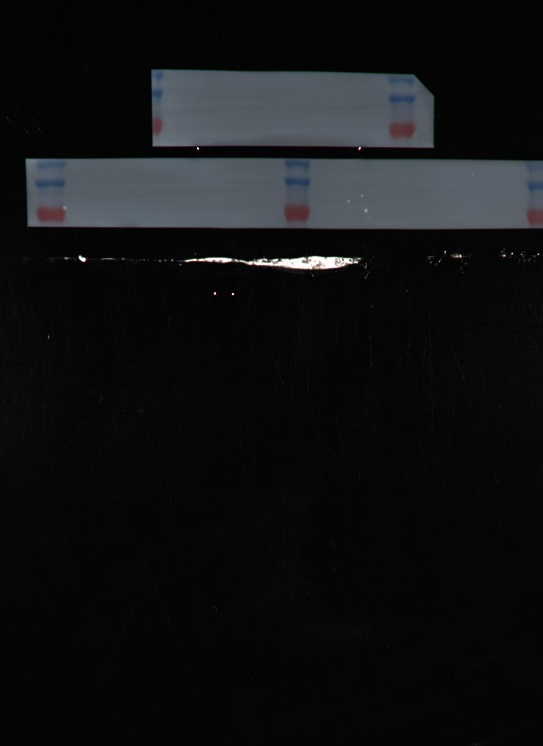


GAPDH
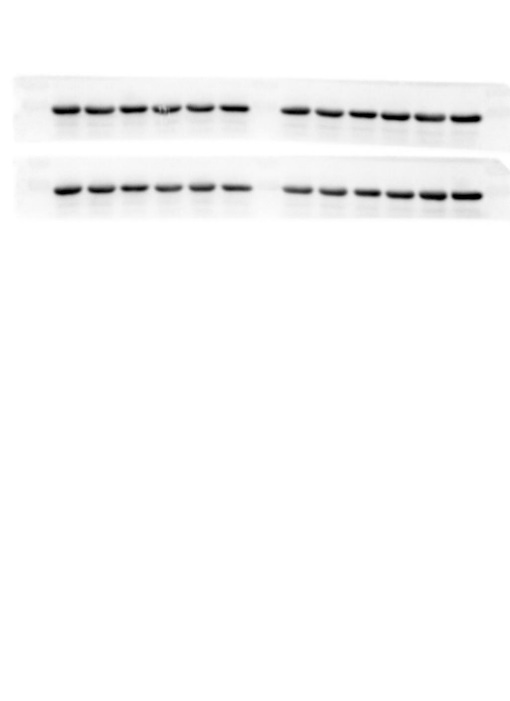

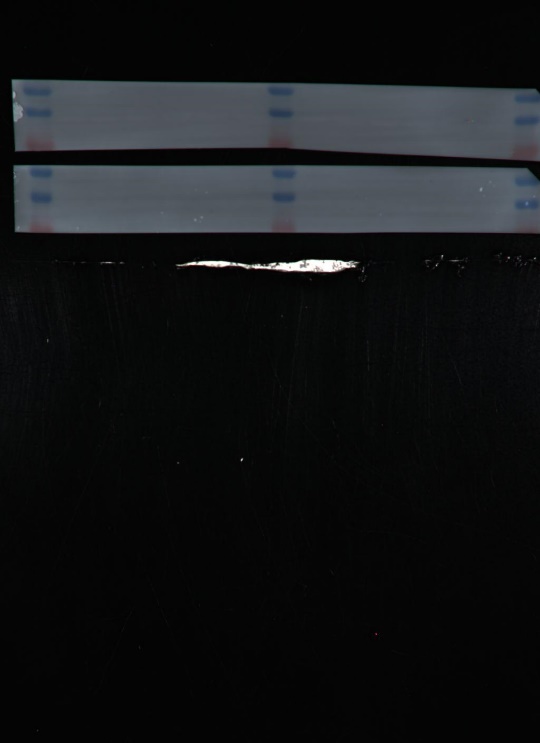


**Figure5**

p-DRP1
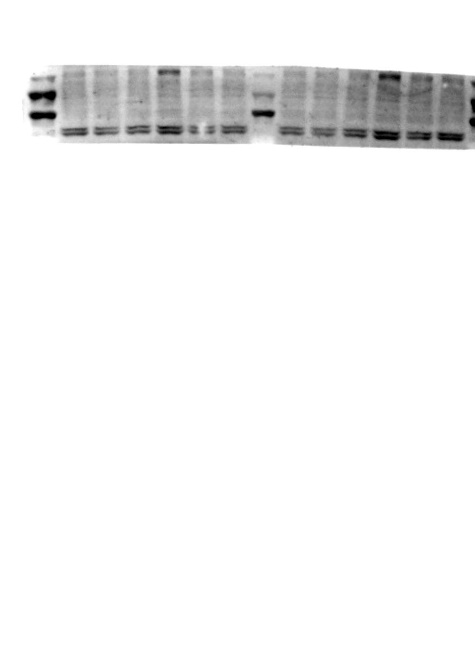

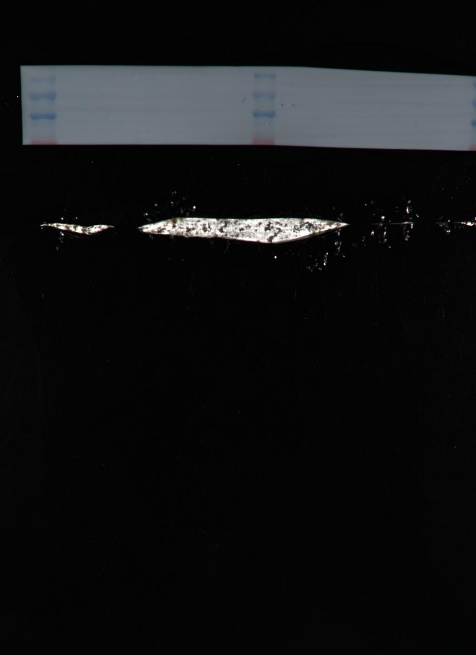


DRP1
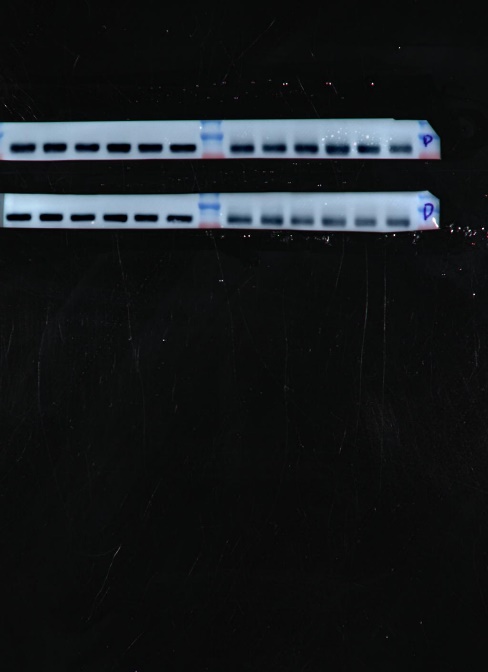


GAPDH
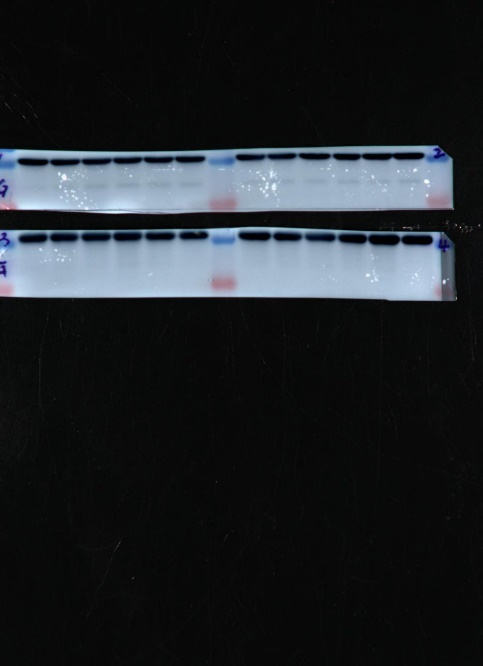


MFF
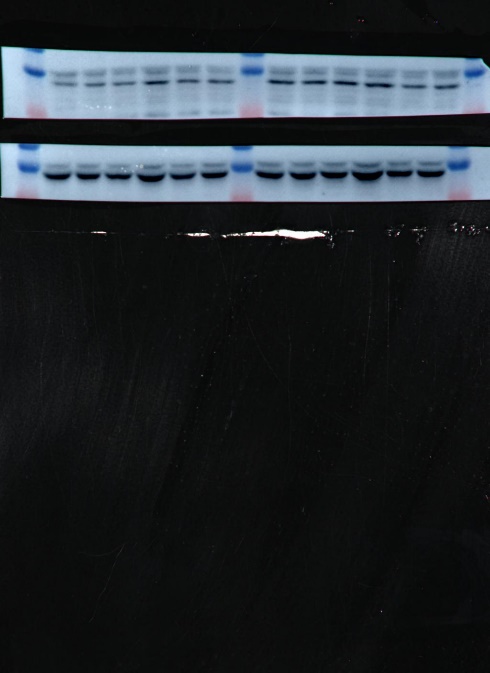


GAPDH
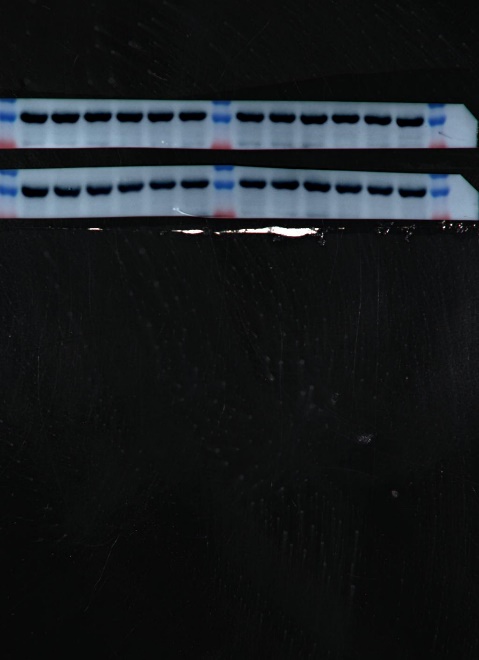


MFN2
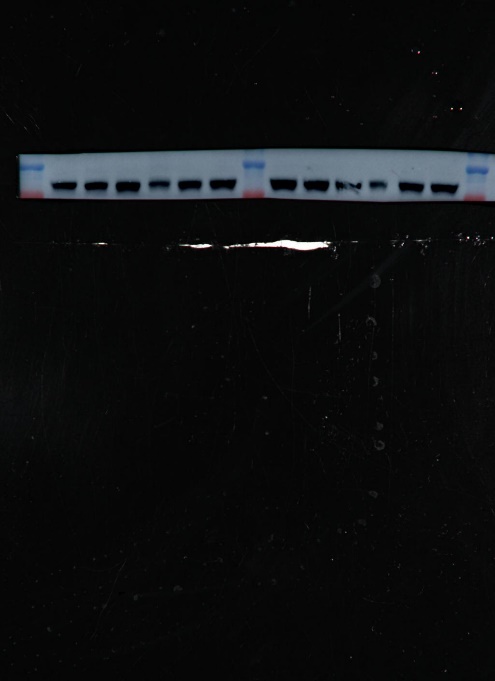


GAPDH
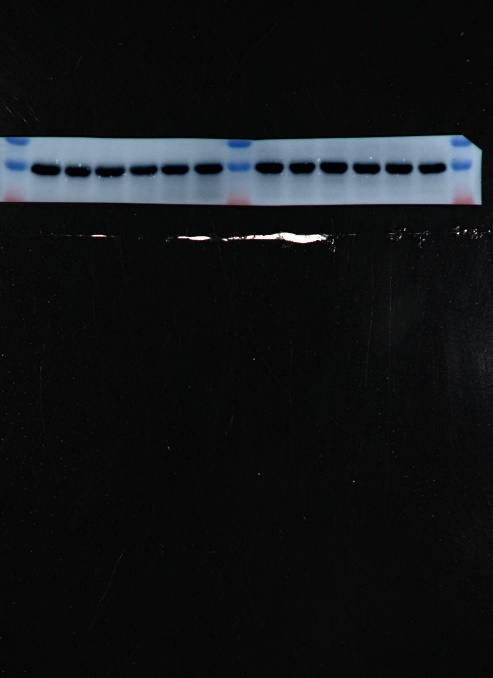


OPA1
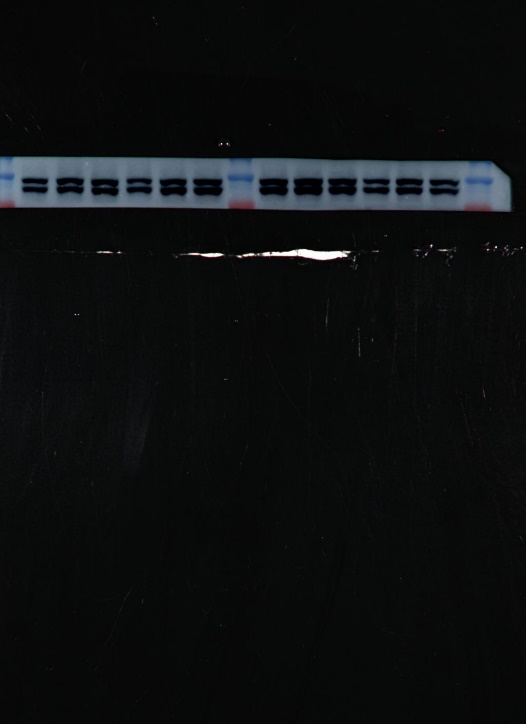


GAPDH
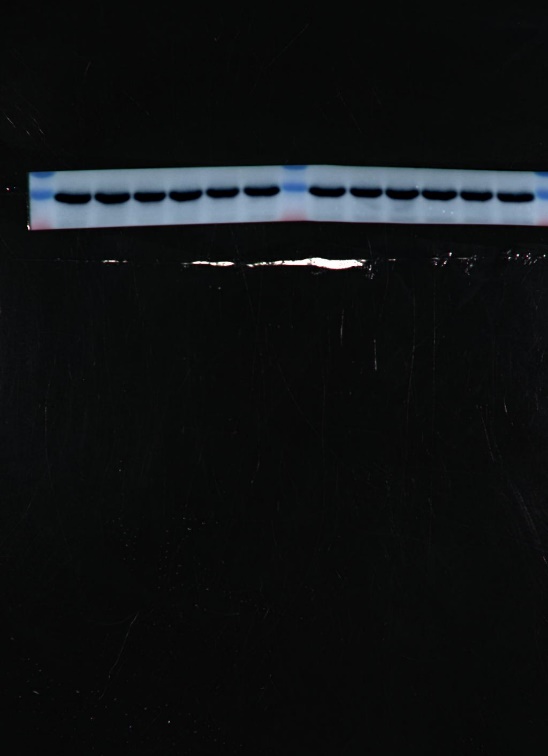


PINK1
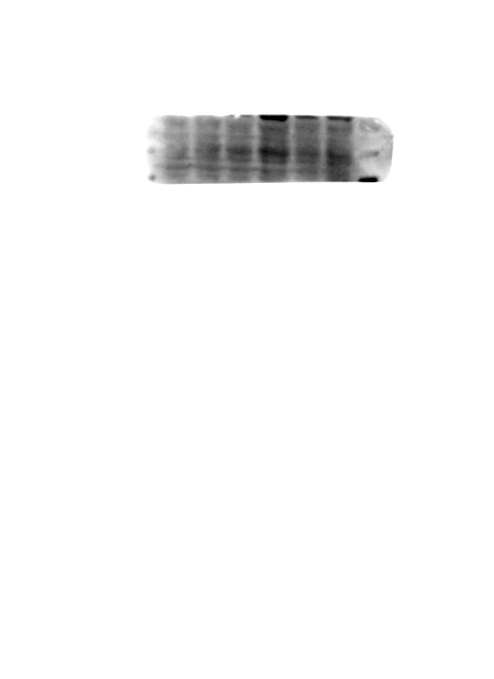

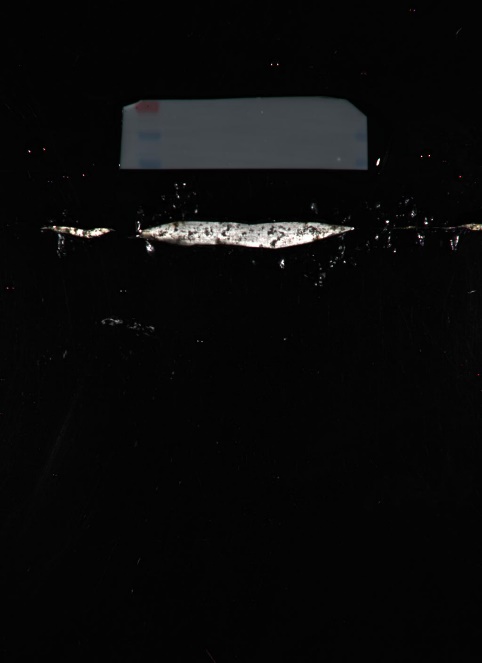


GAPDH
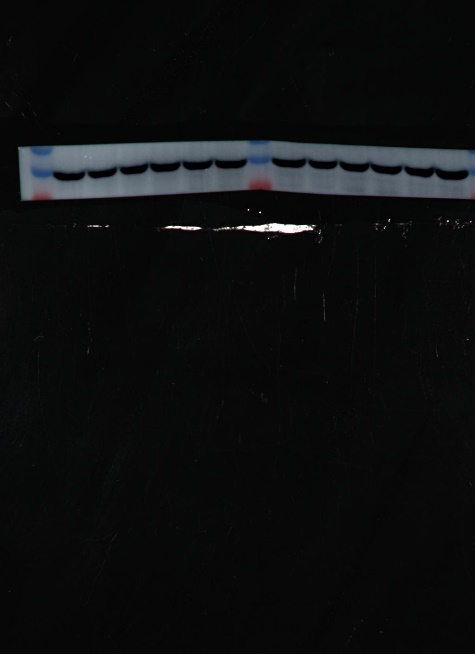


SQSTM1/P62
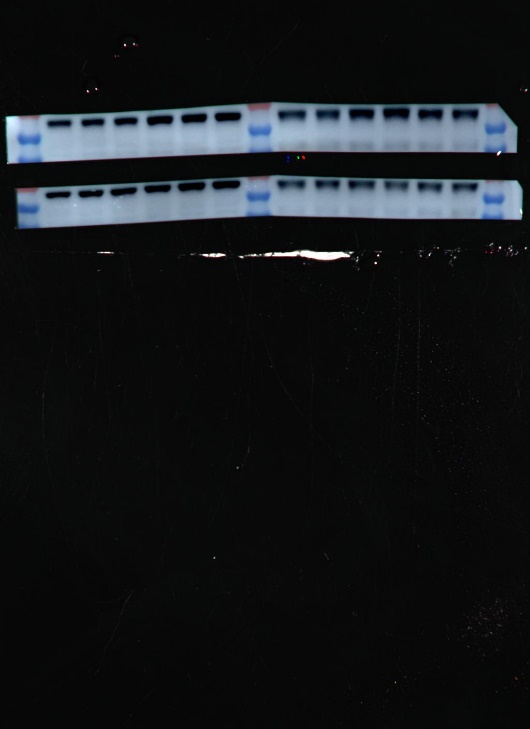


GAPDH
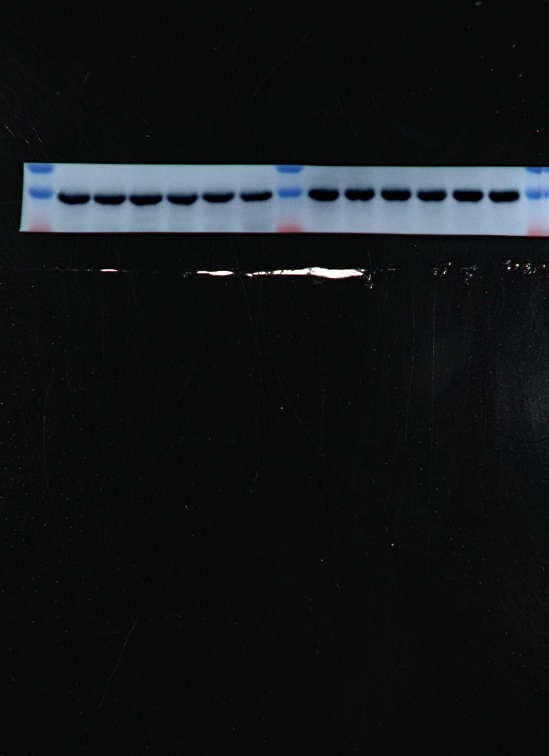


PARK2
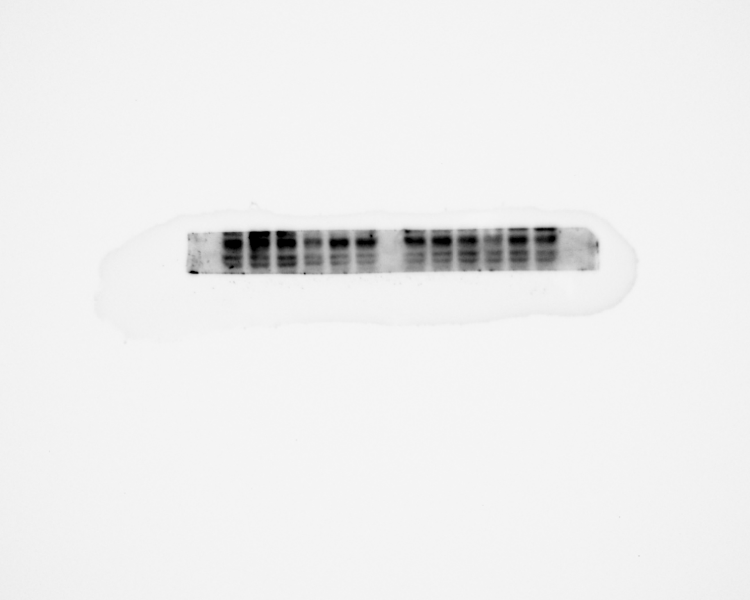

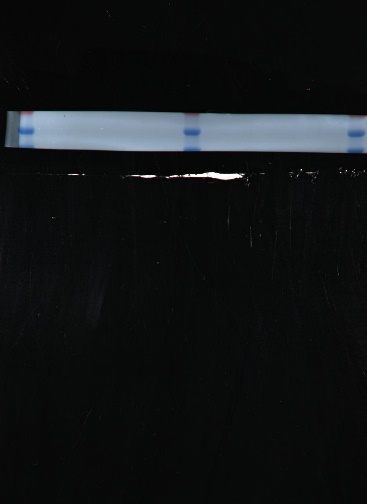


GAPDH
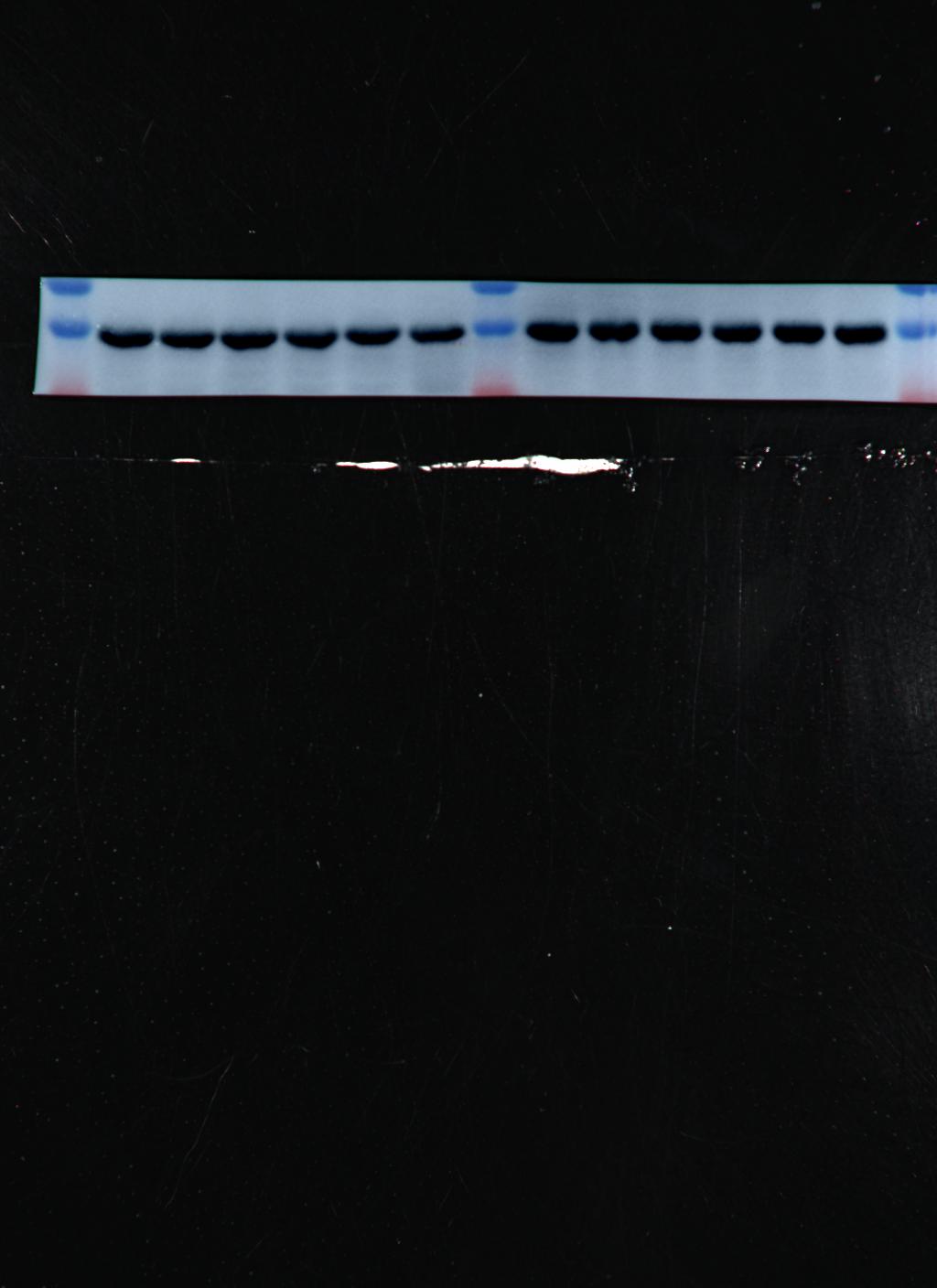


LC3B
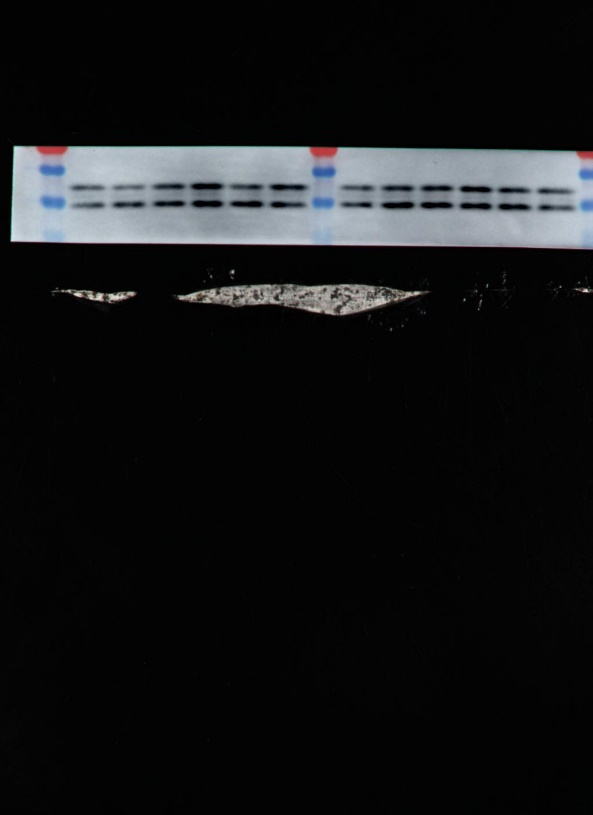


β-actin
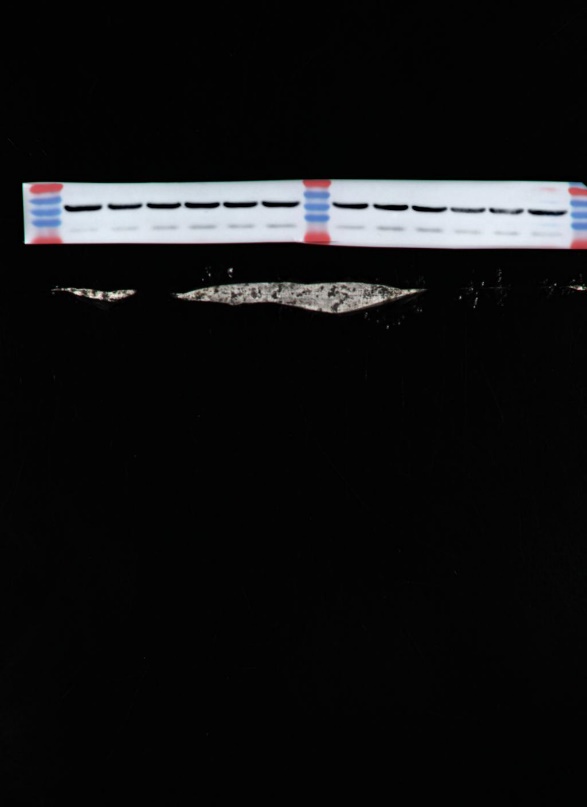


MLKL
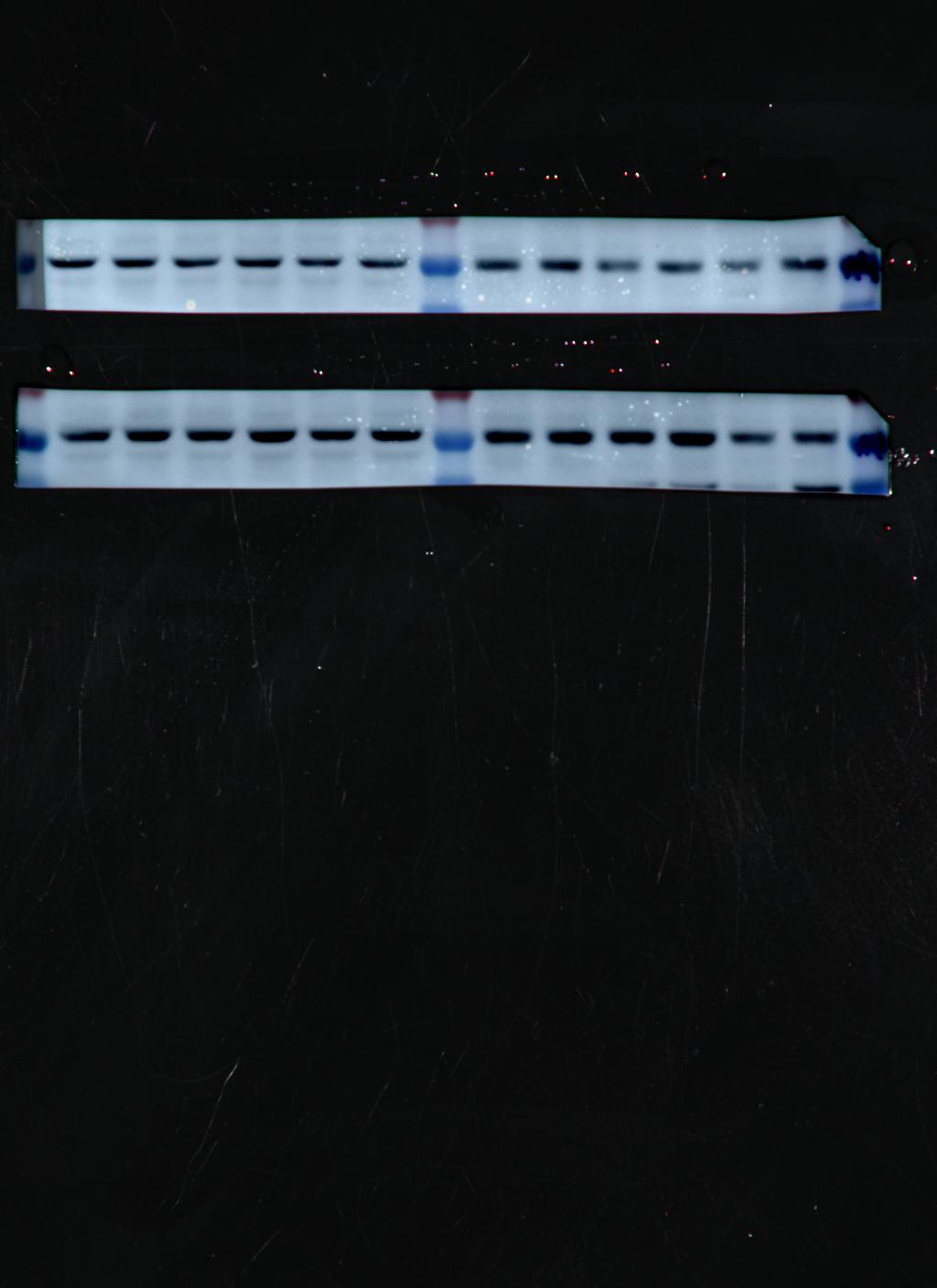


GAPDH
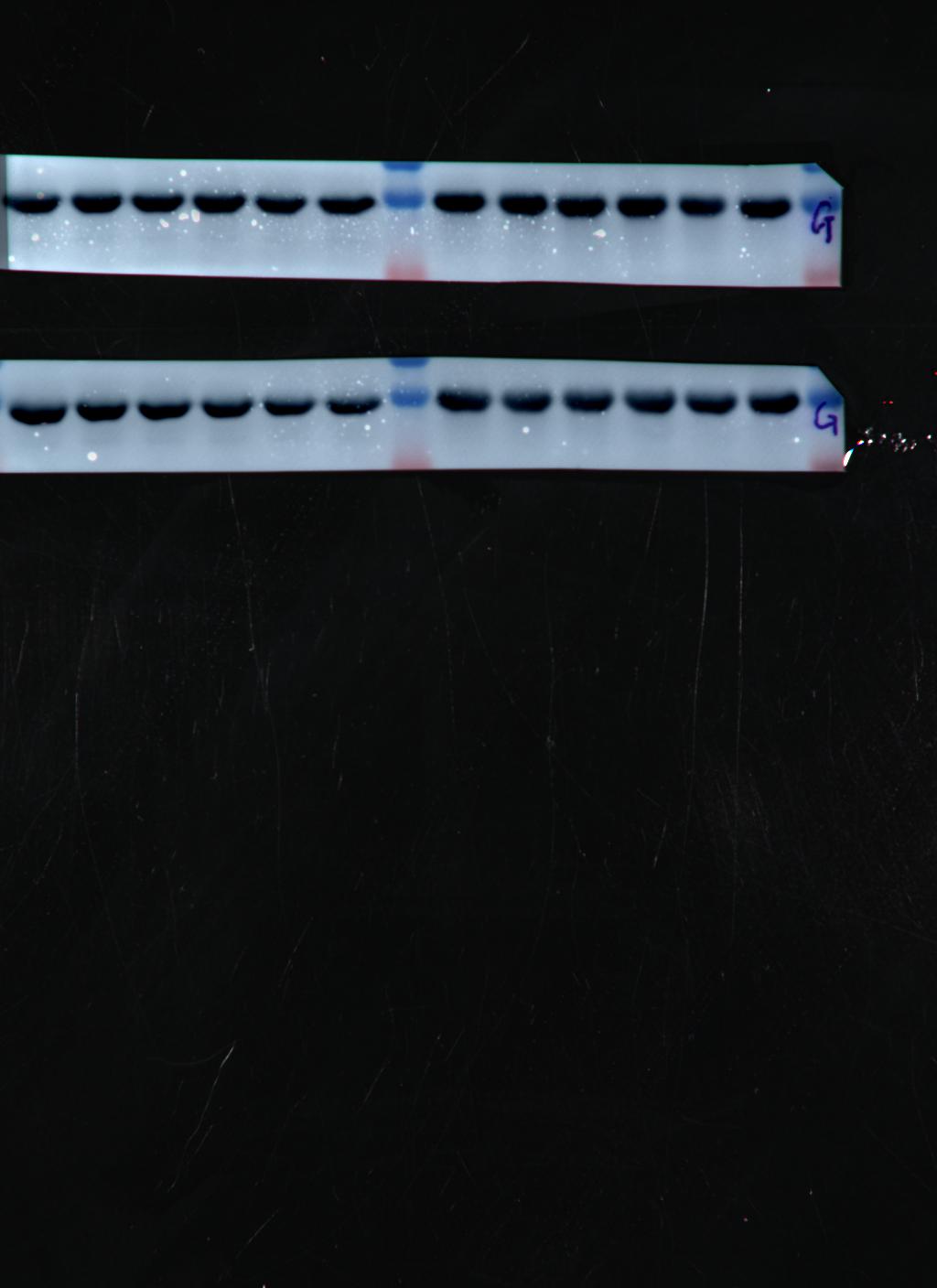


RIPK1
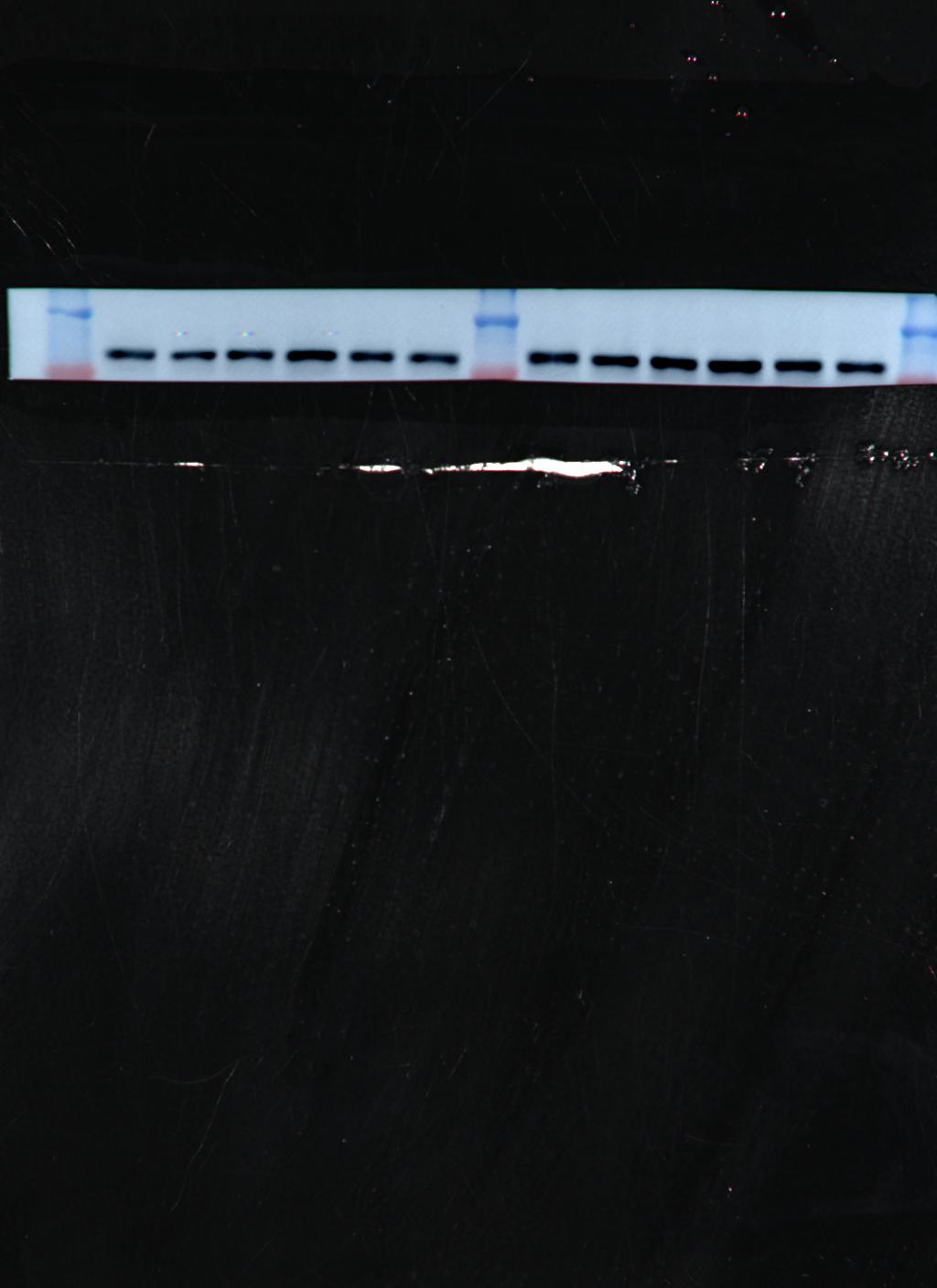


GAPDH
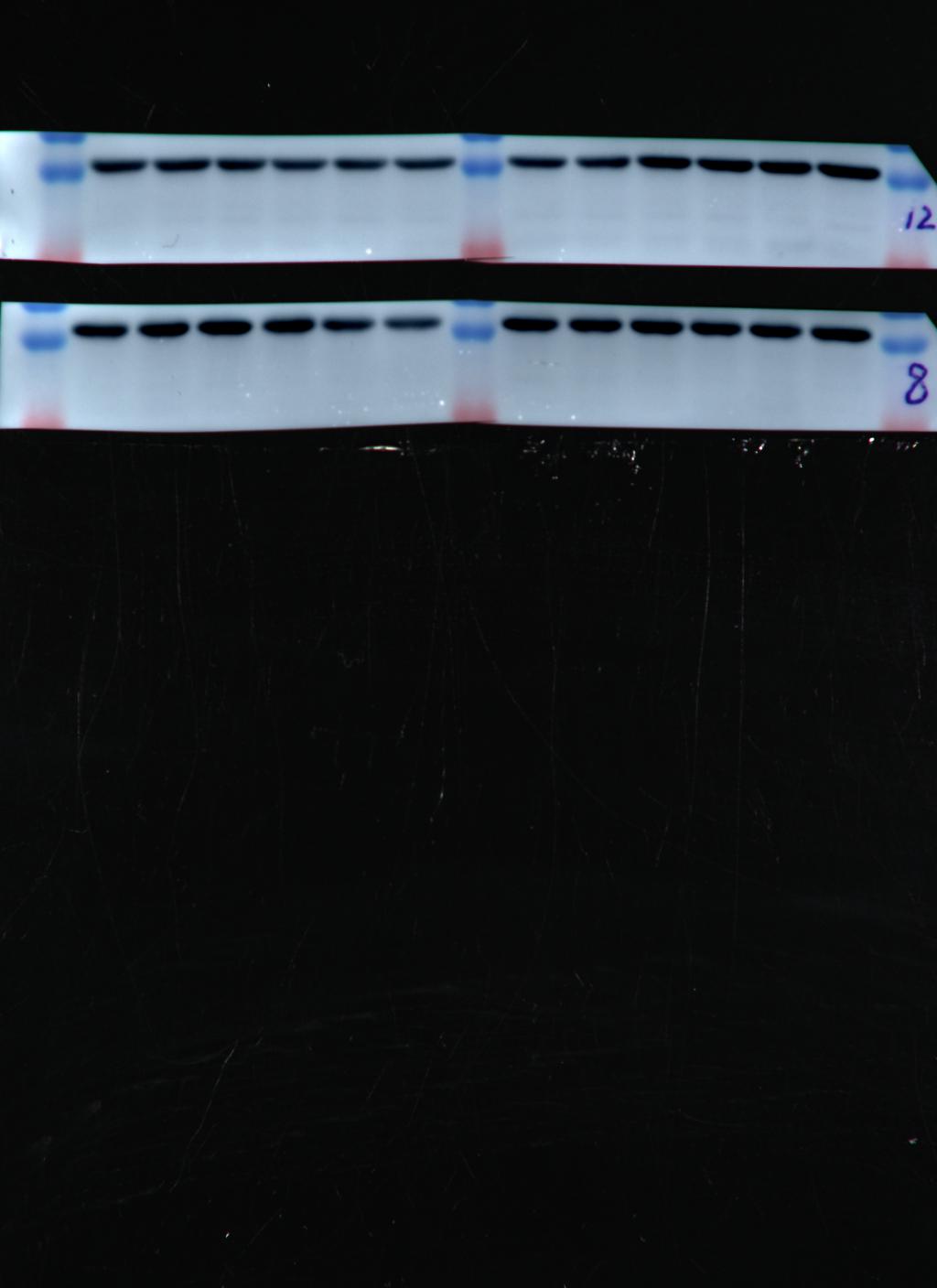


RIPK3
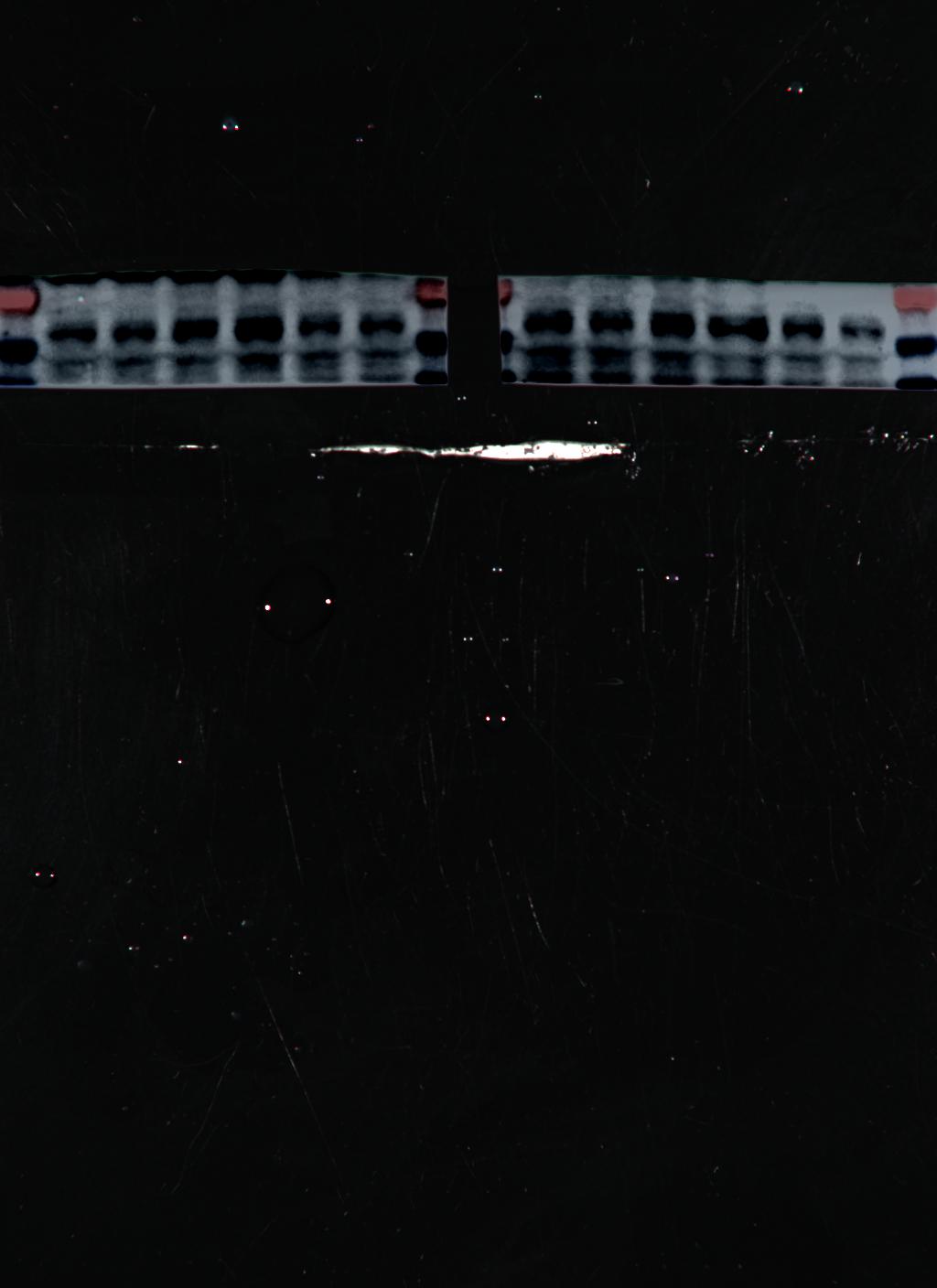


GAPDH
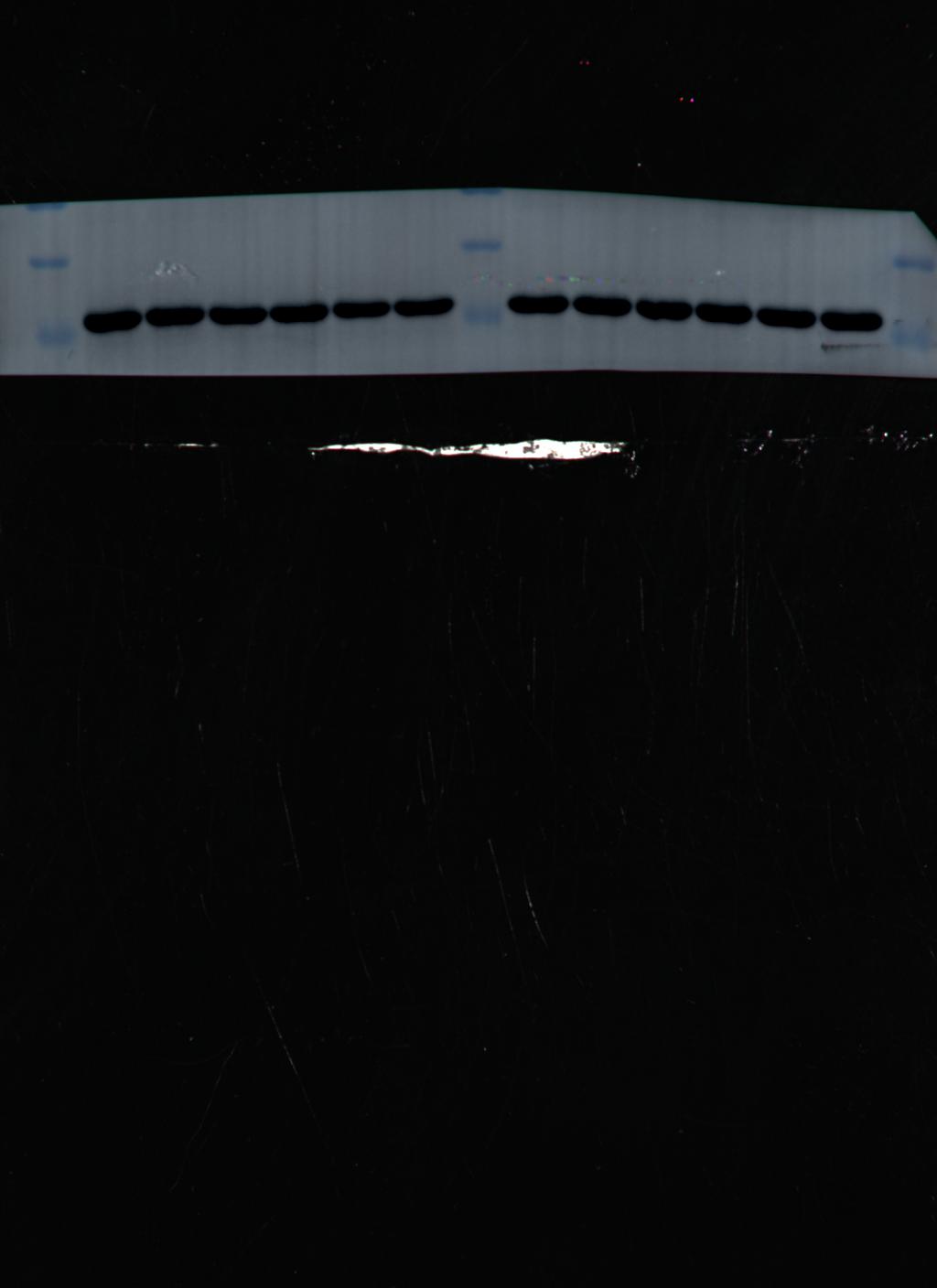


**Figure6**

p-DRP1
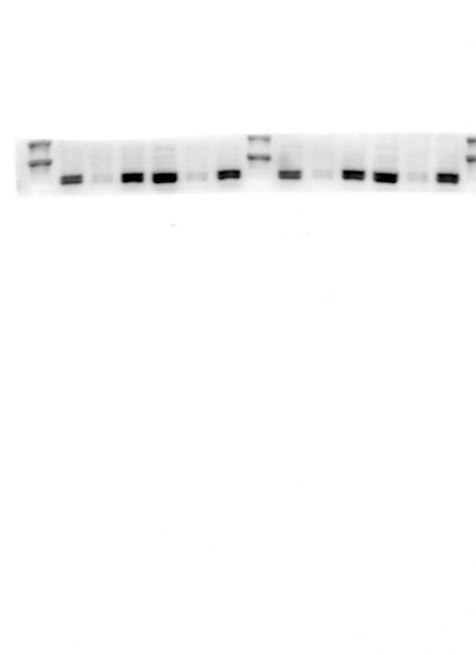

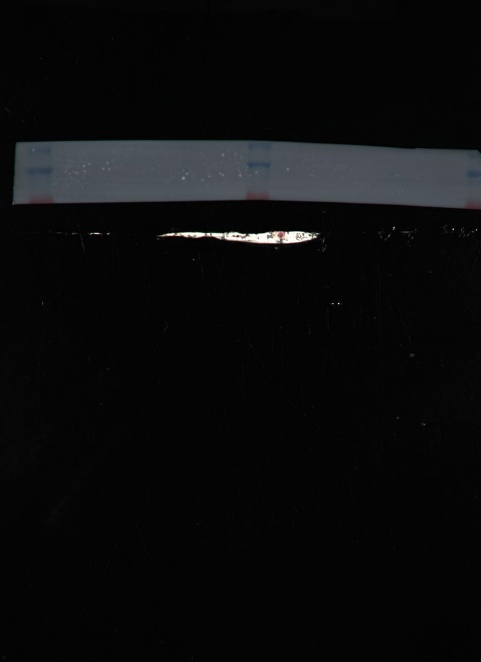


DRP1
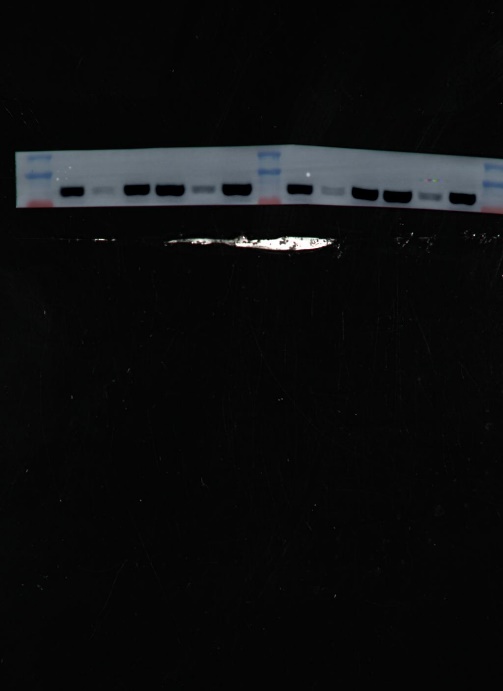


GAPDH
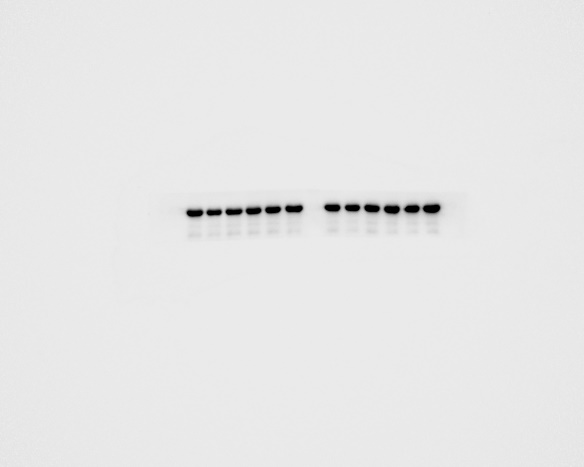

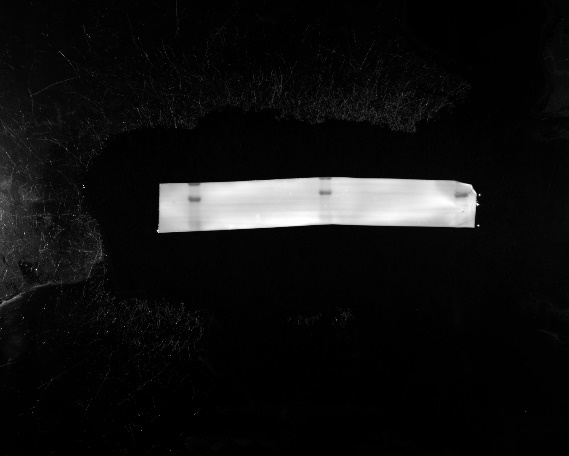


MFF
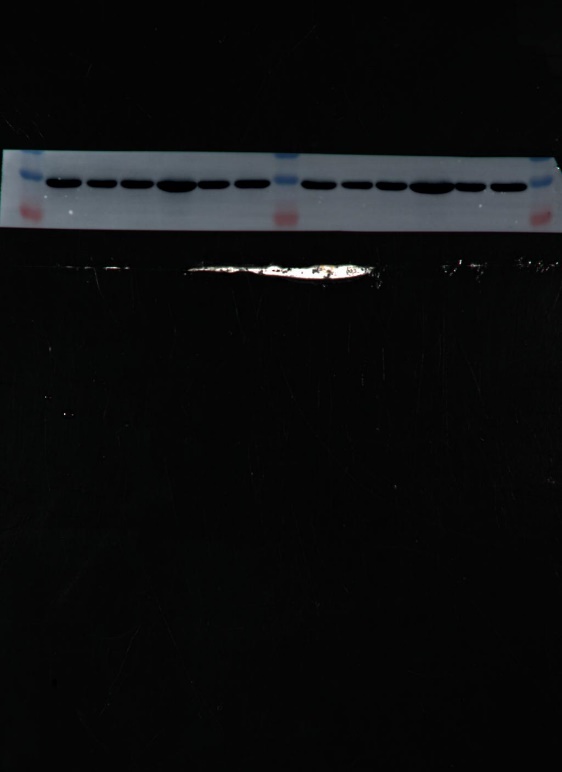


GAPDH
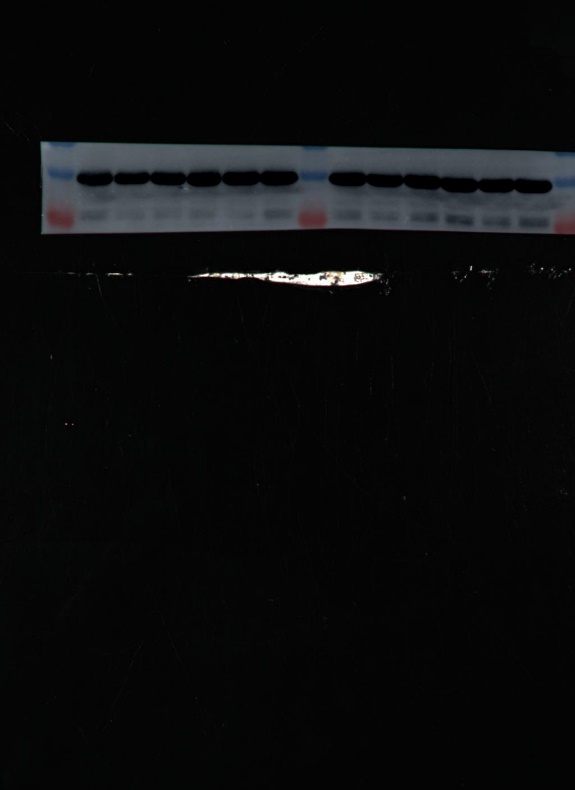


MFN2
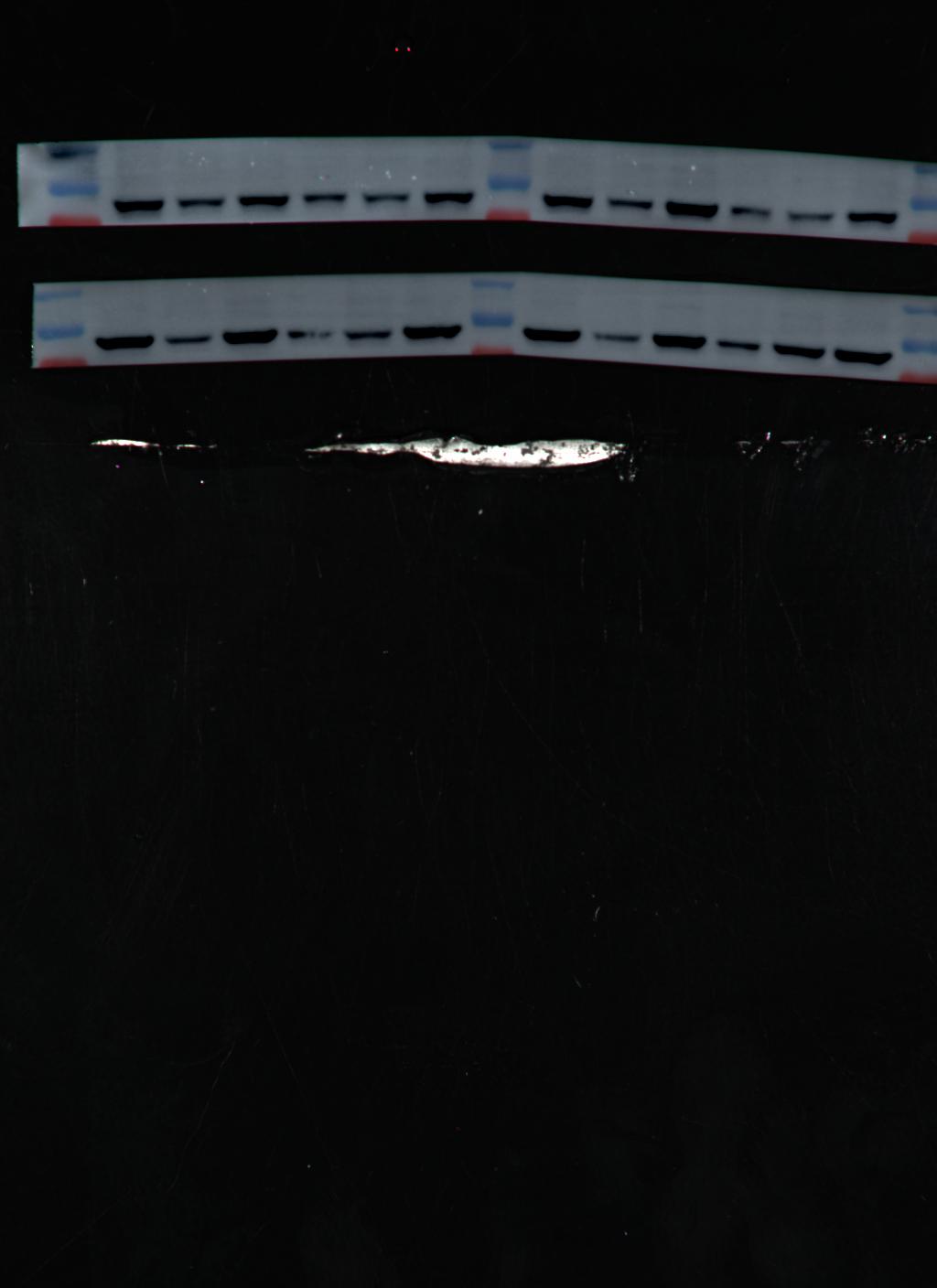


GAPDH
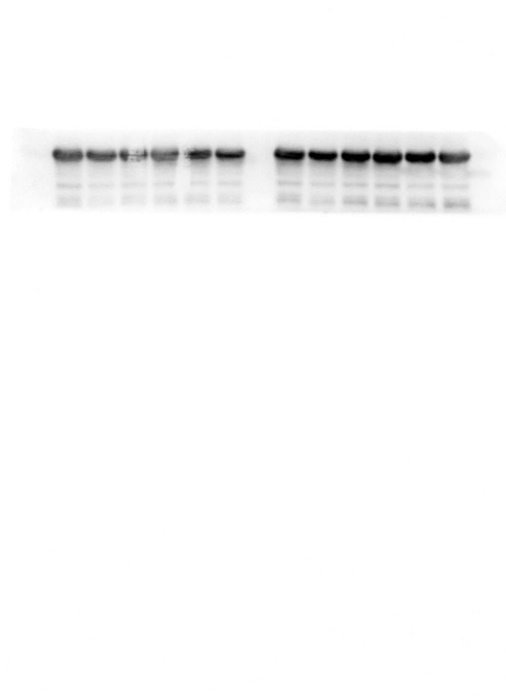

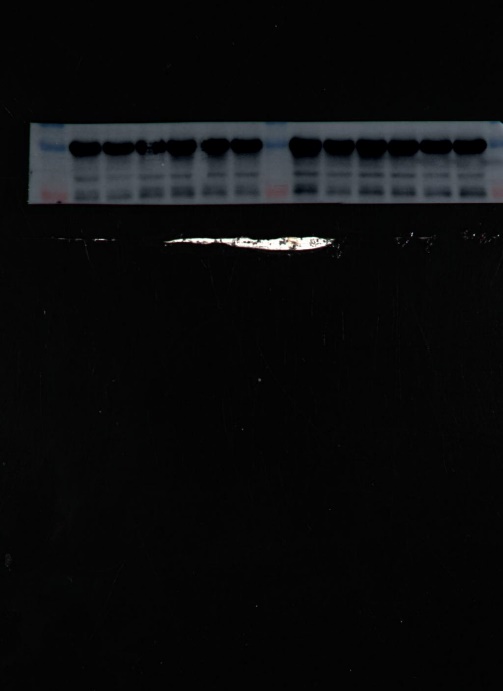


OPA1
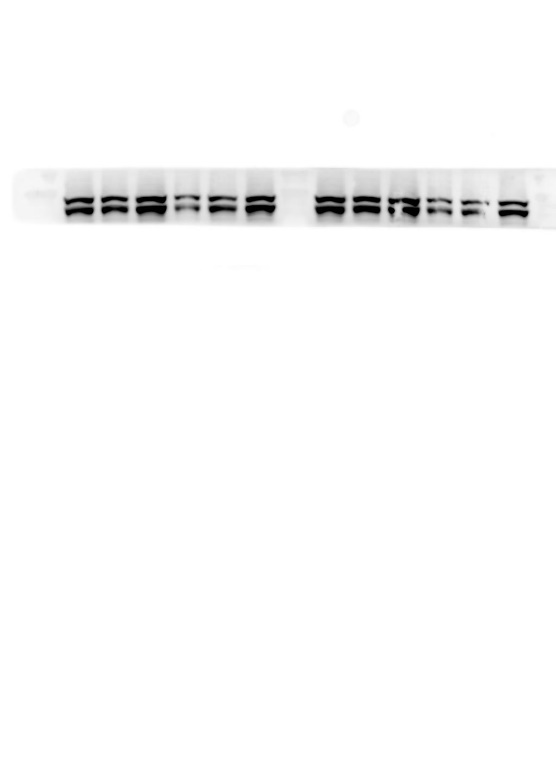

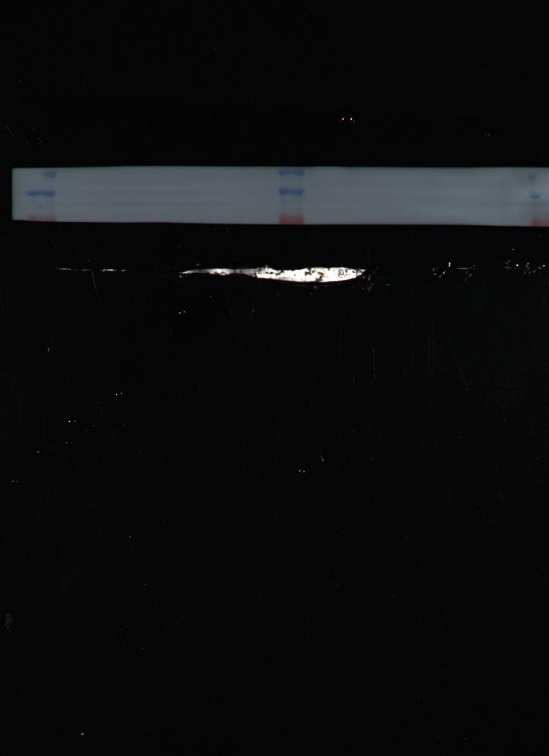


GAPDH
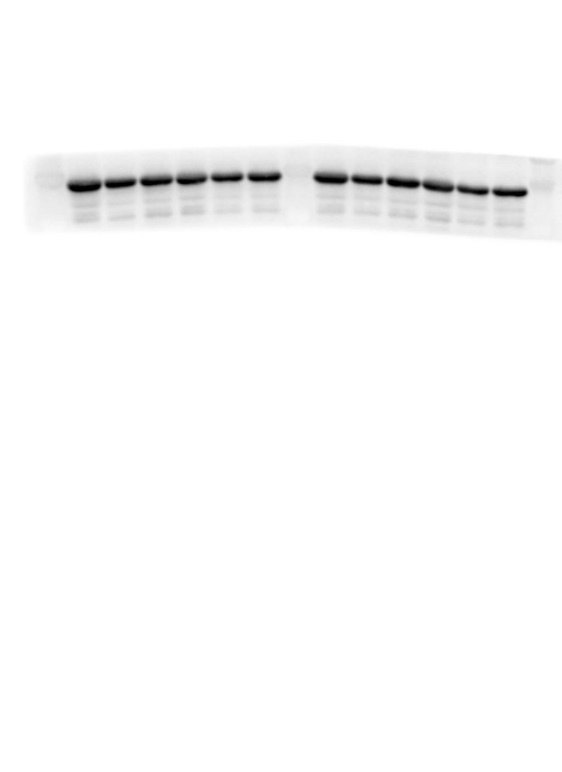

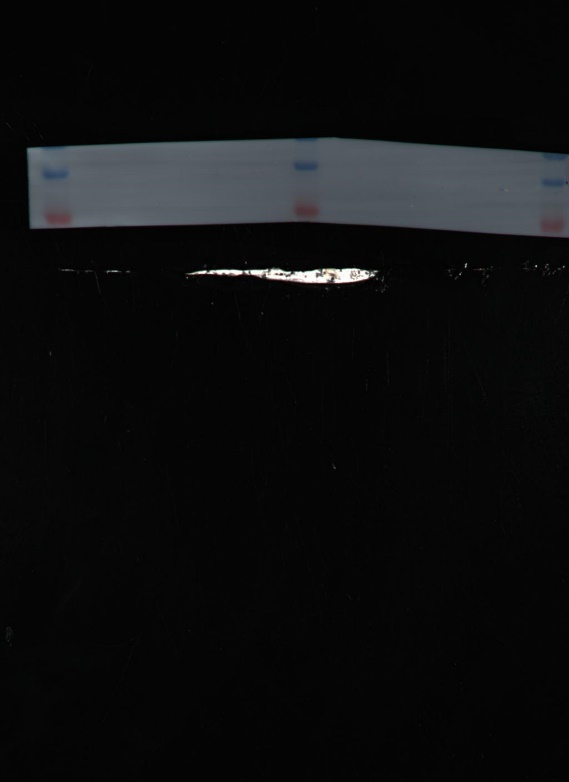


PINK1
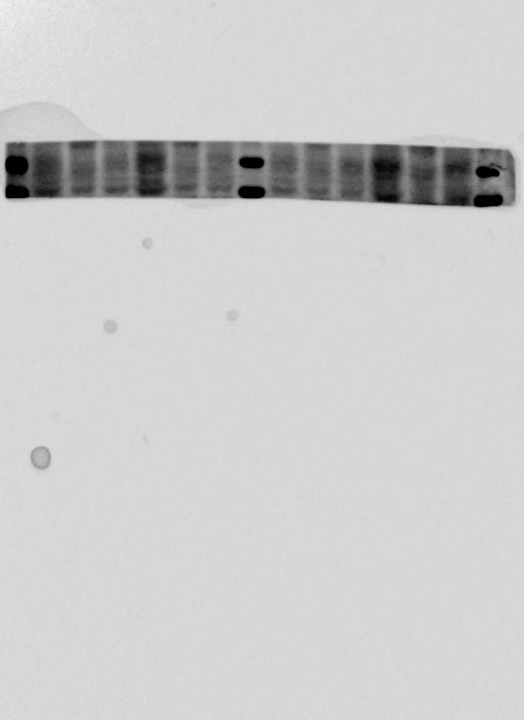

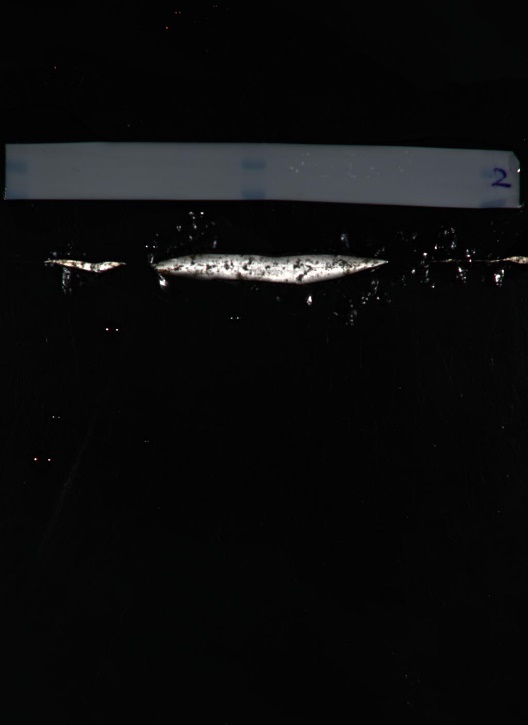


GAPDH
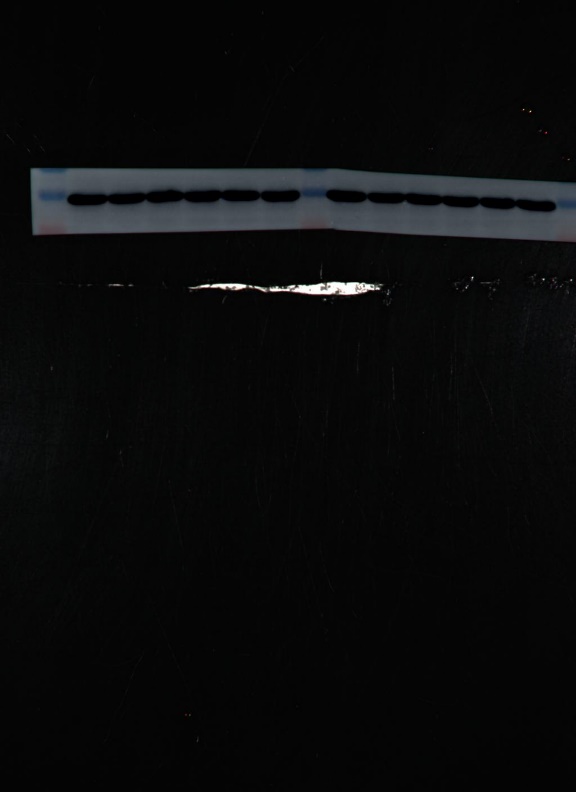


SQSTM1/P62
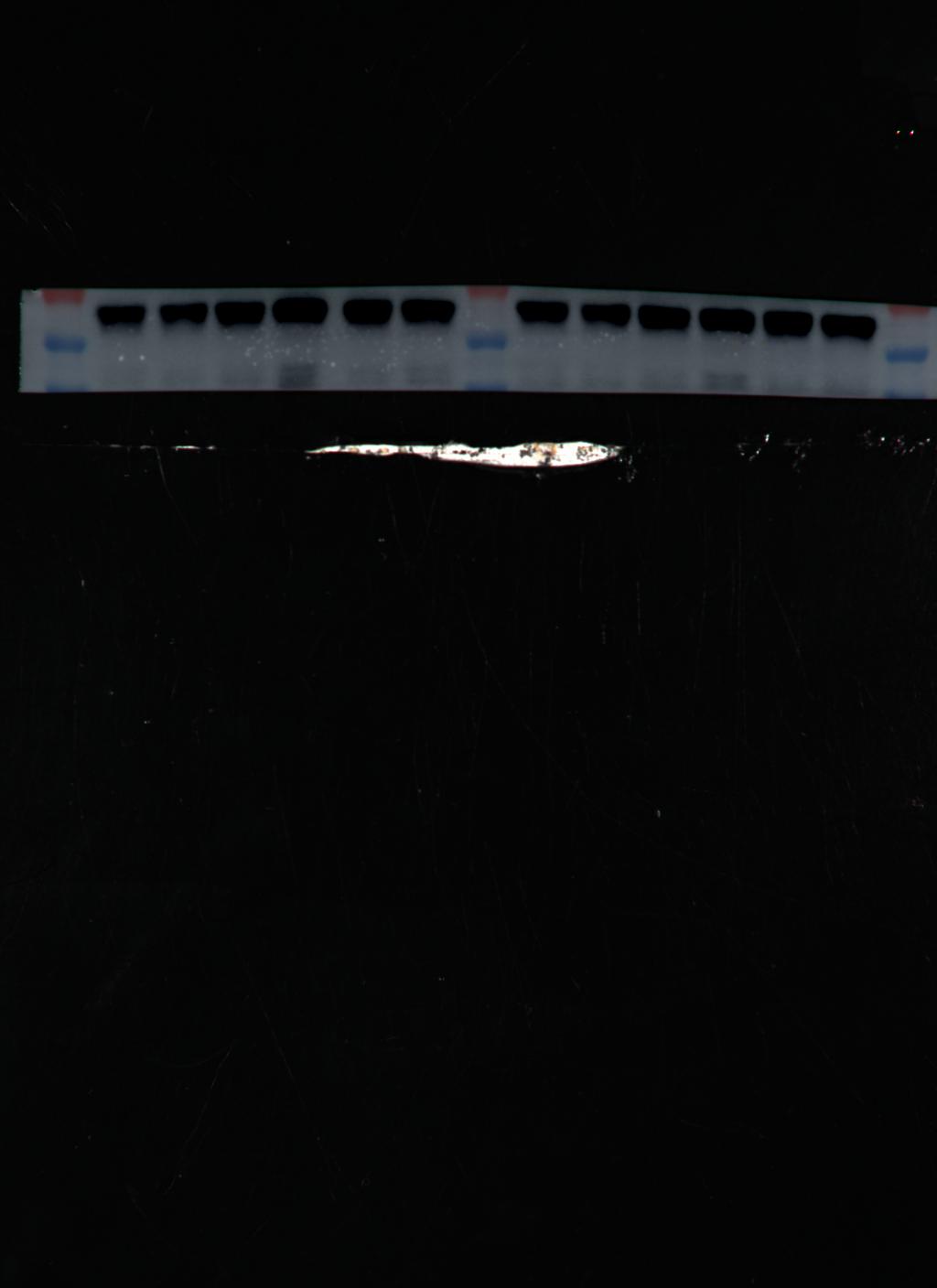


GAPDH
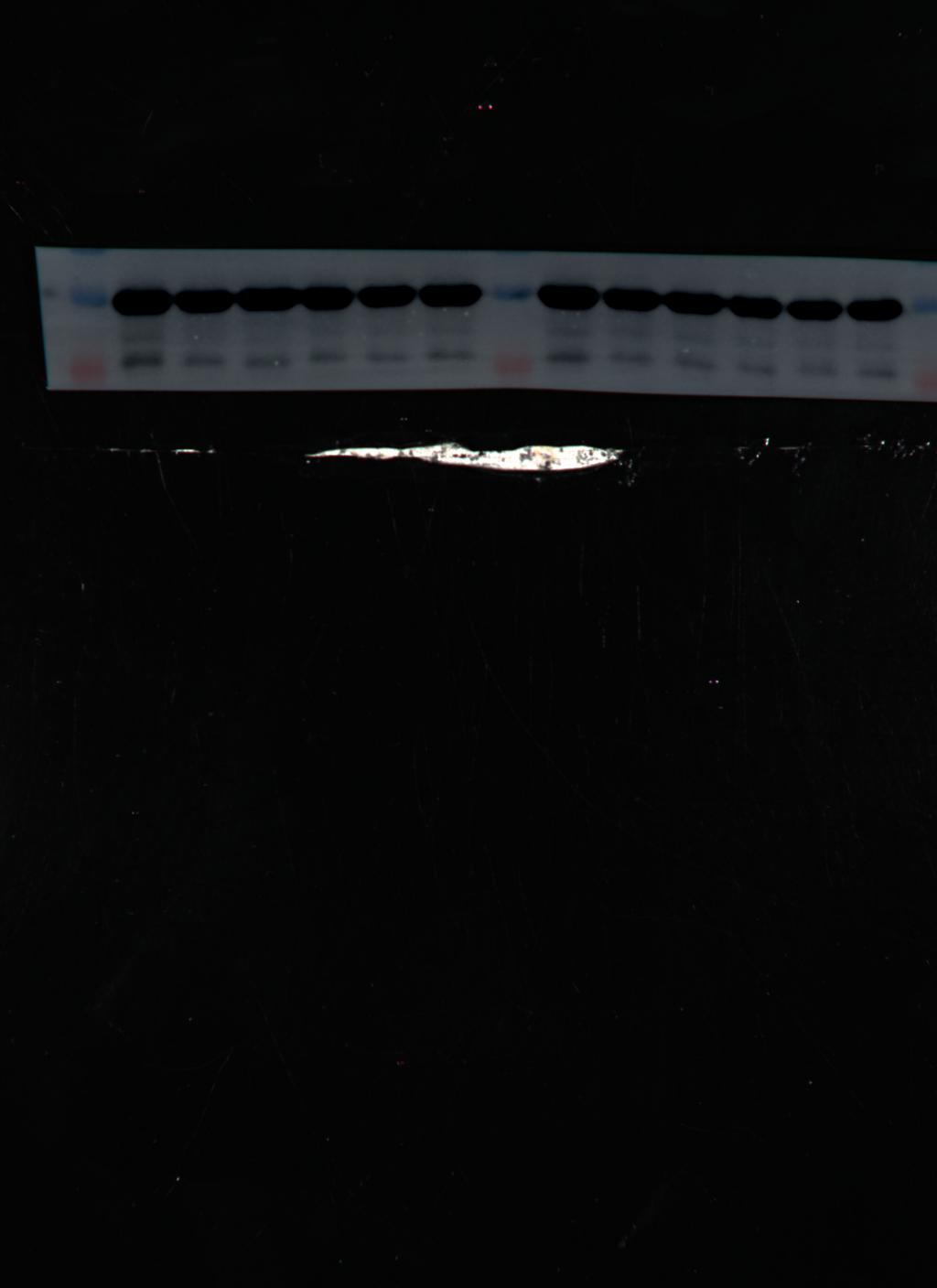


PARK2
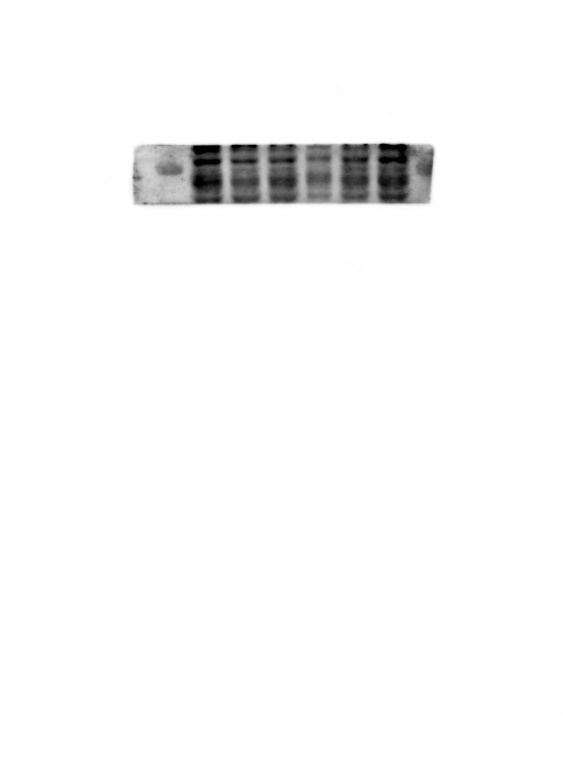

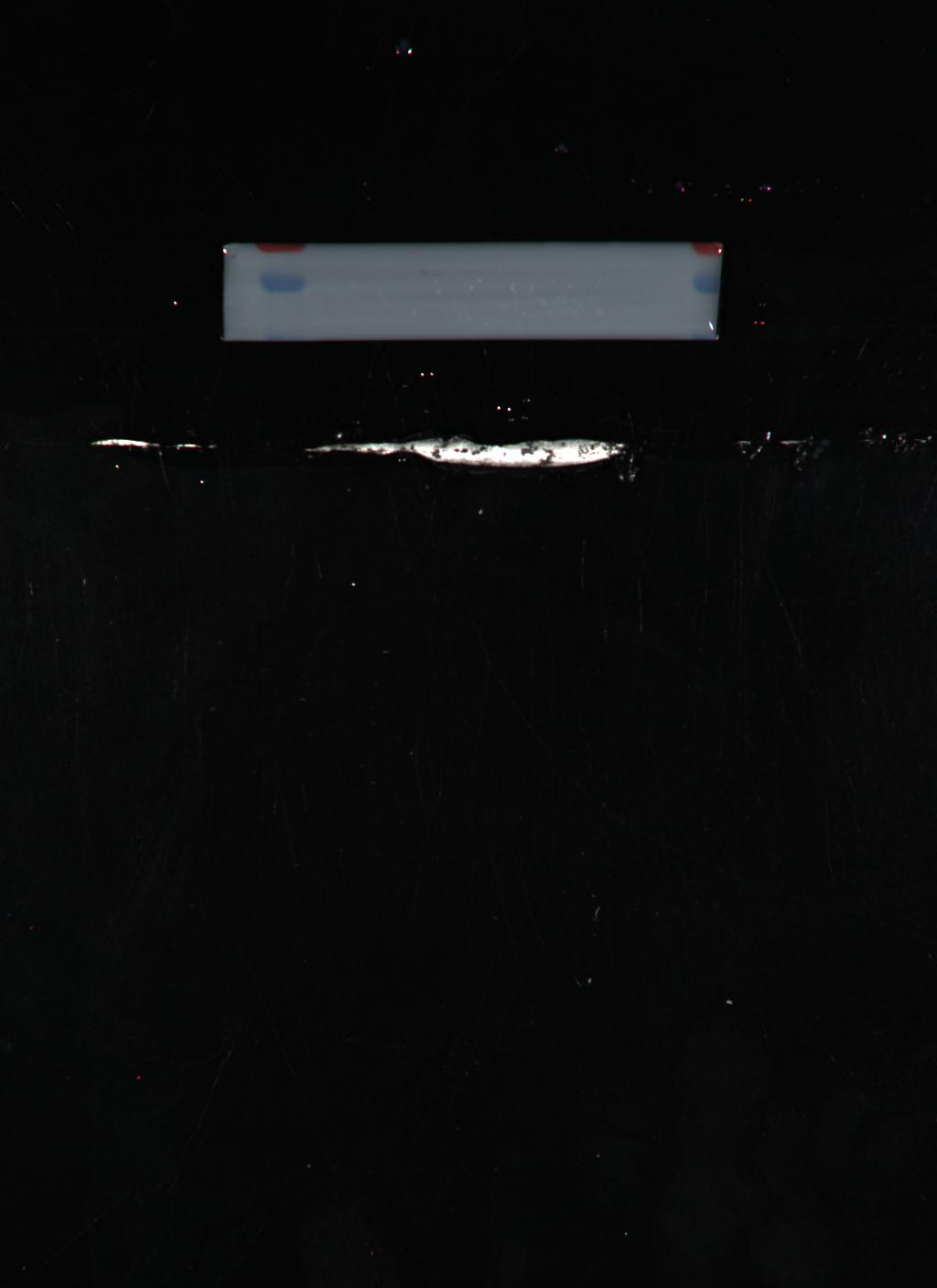


GAPDH
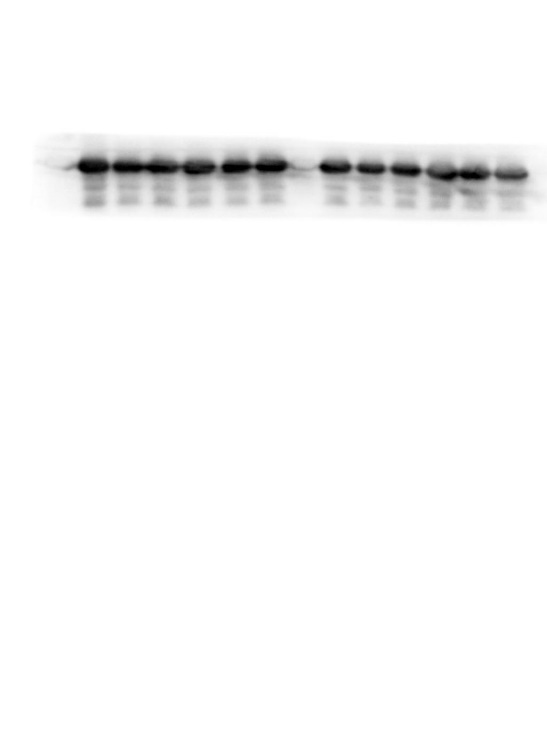

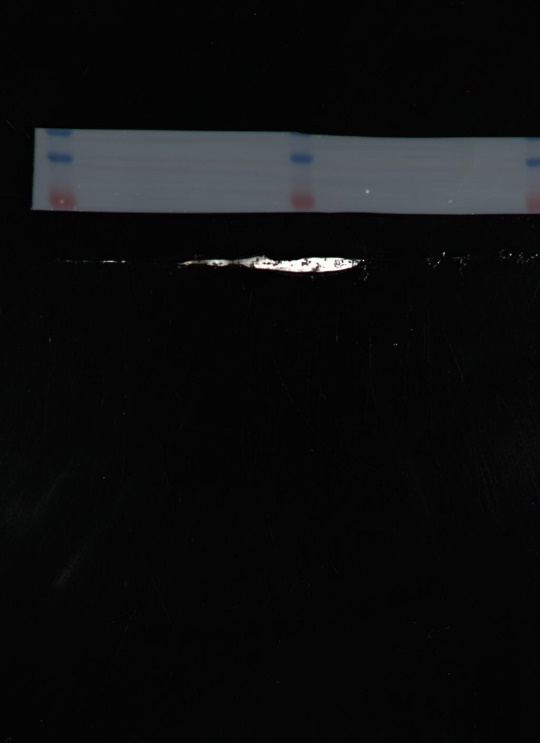


LC3B
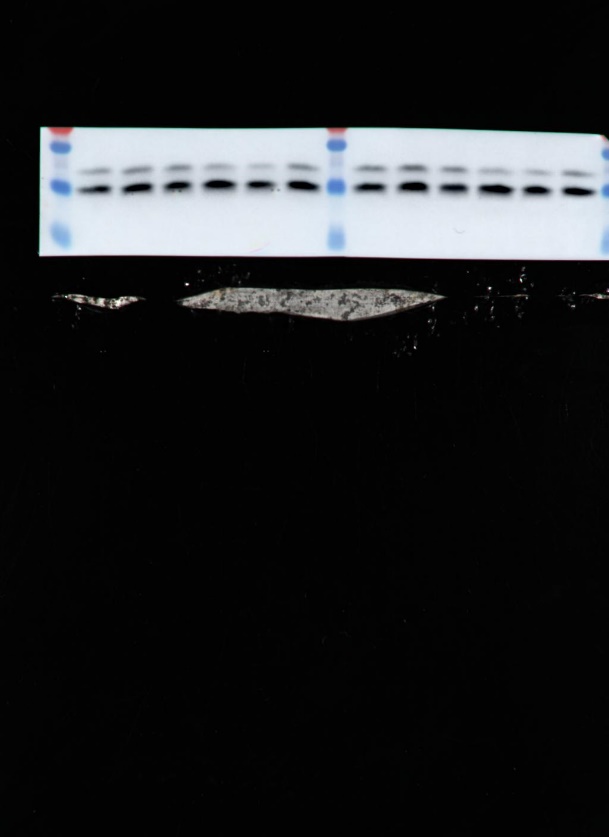


β-actin
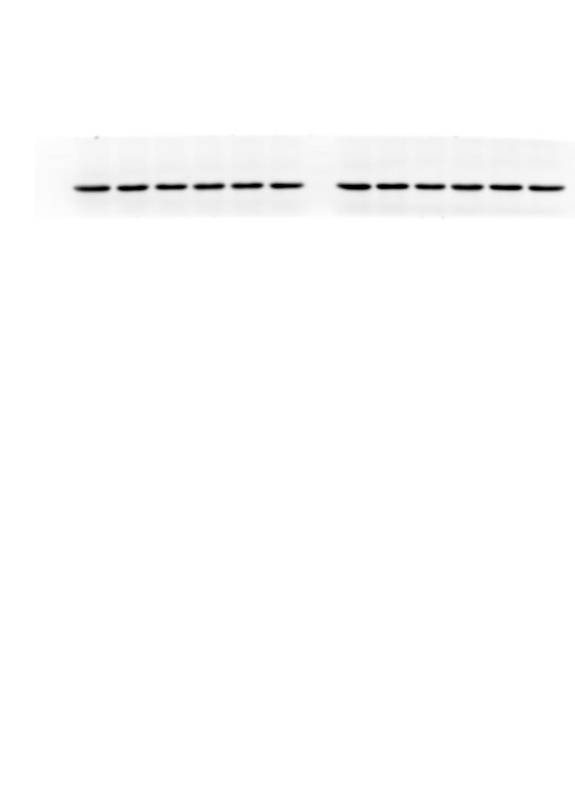

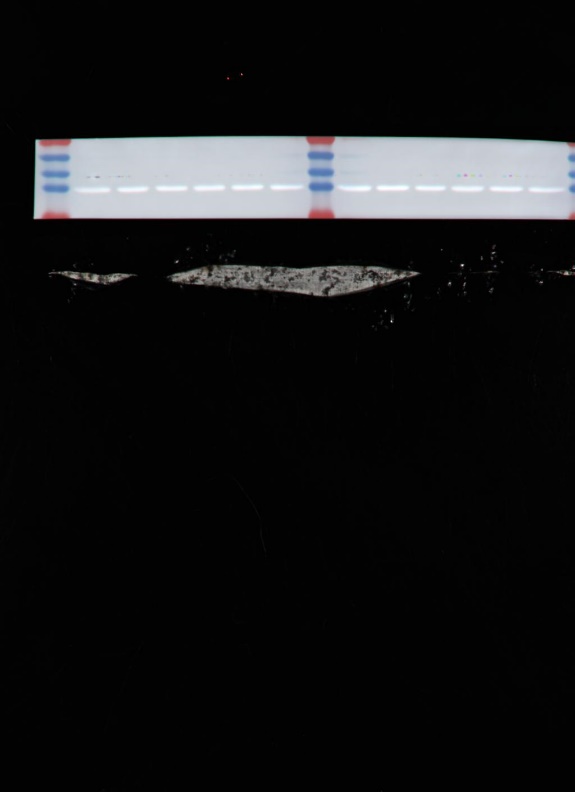


MLKL
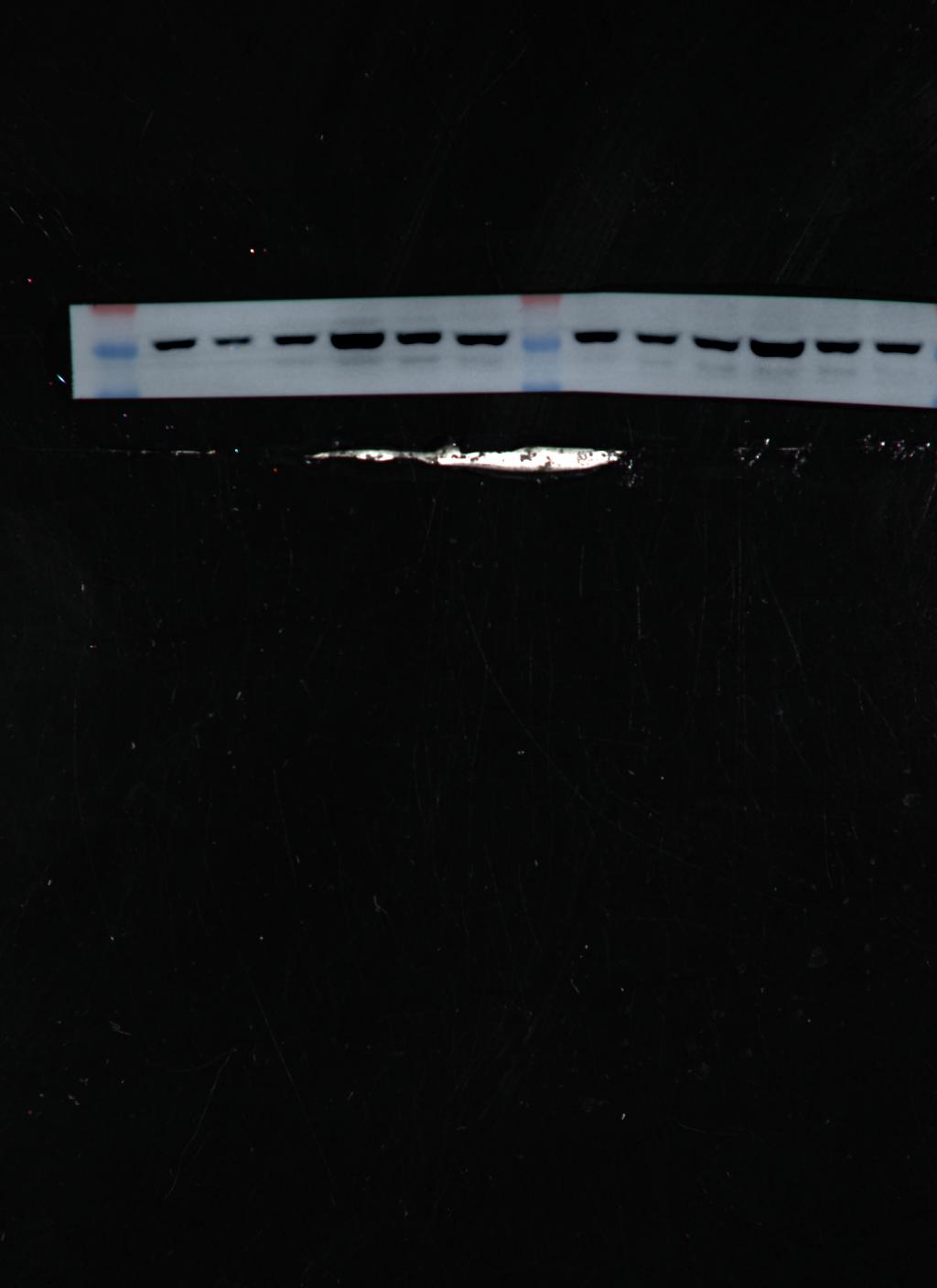


GAPDH
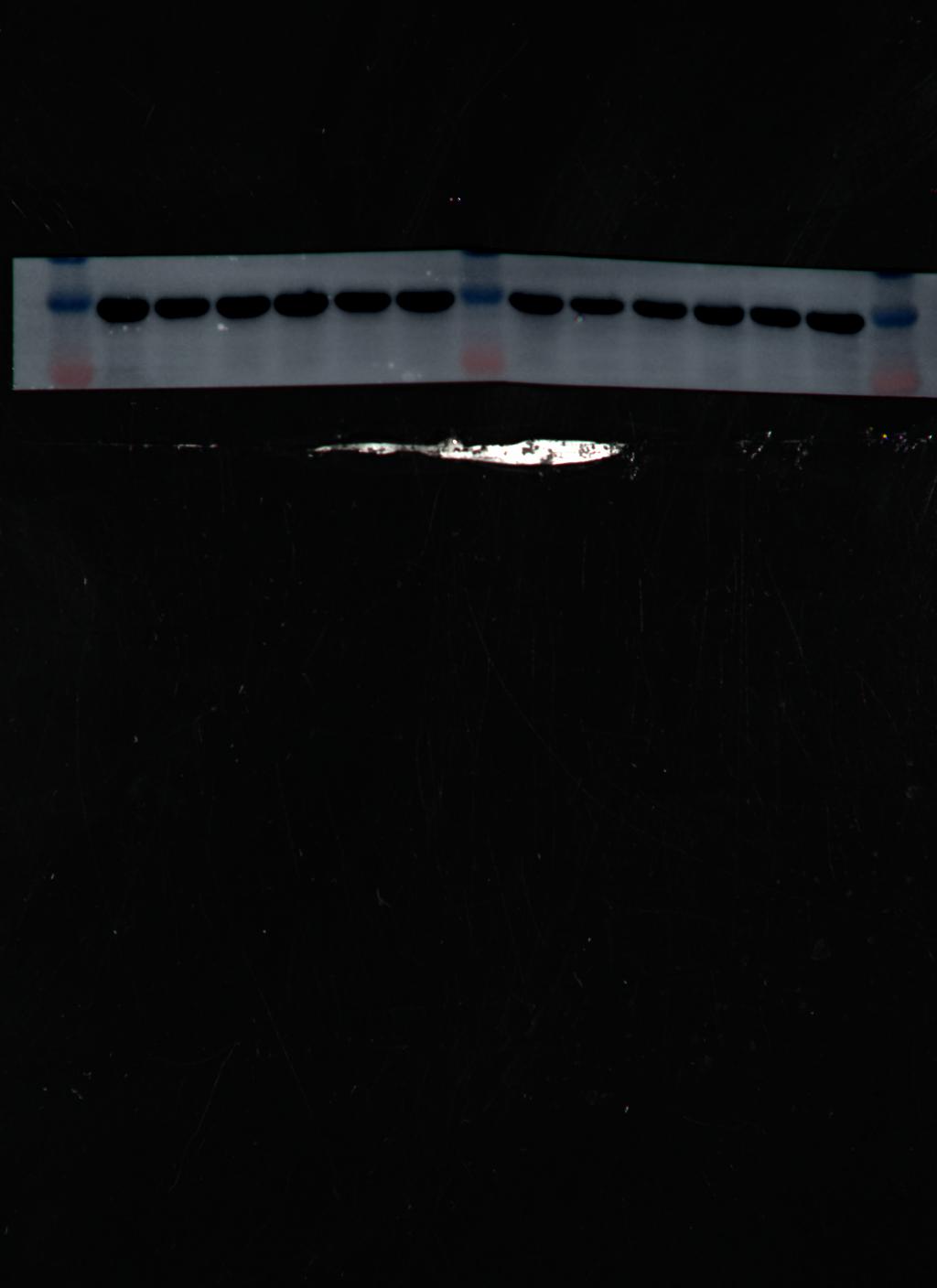


RIPK1
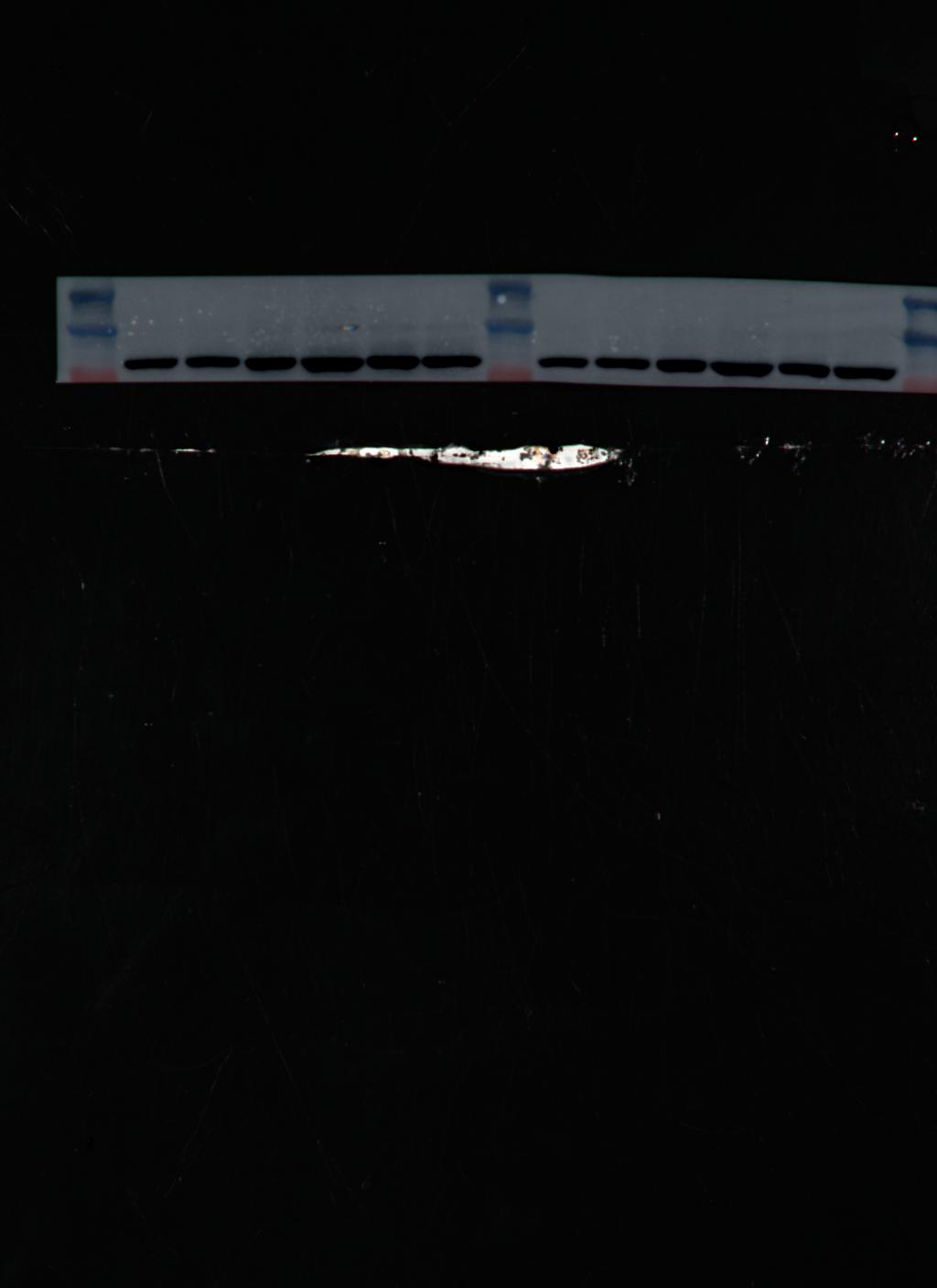


GAPDH
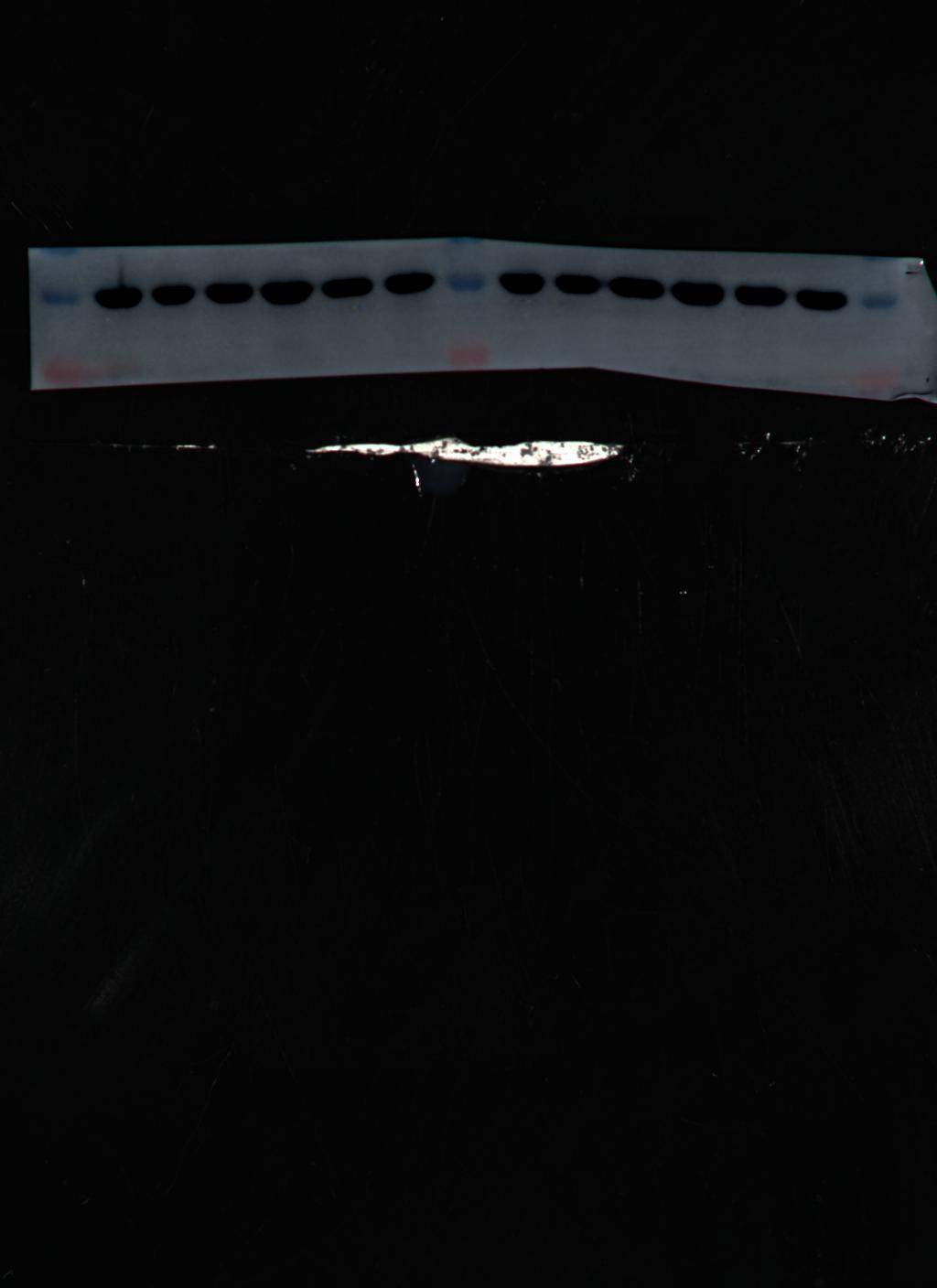


RIPK3
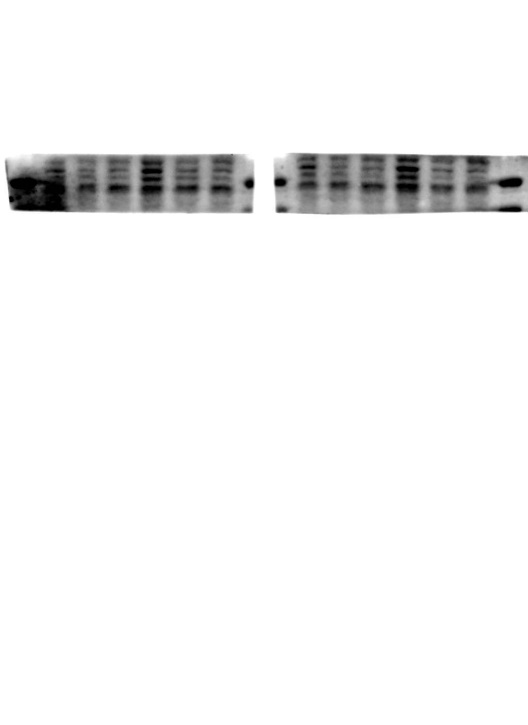

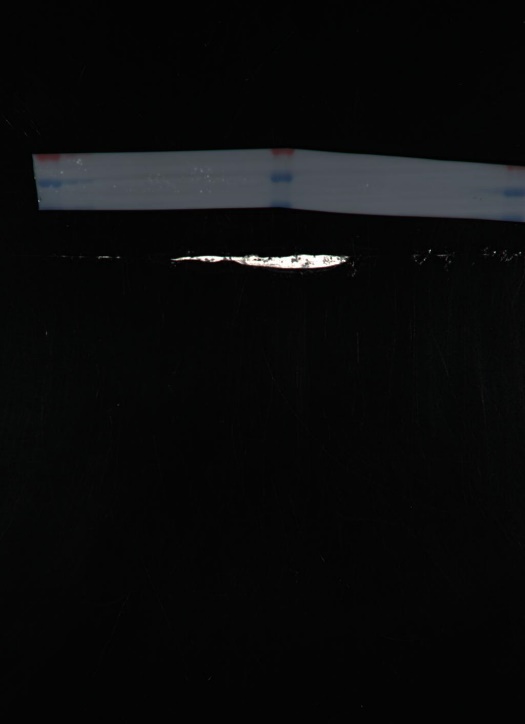


GAPDH
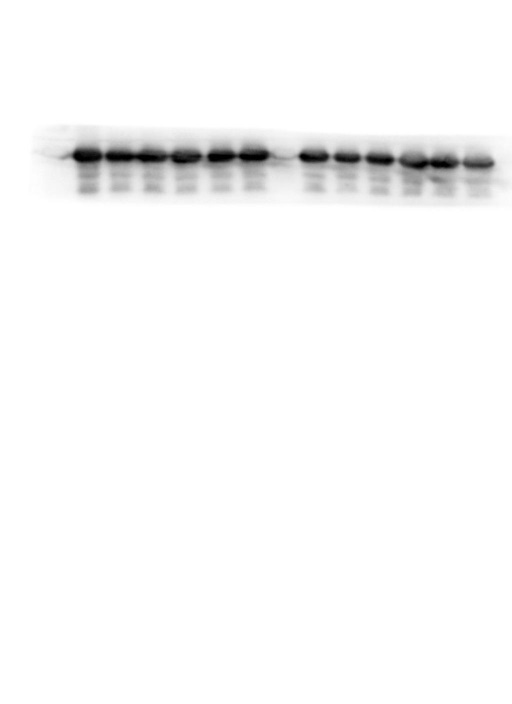

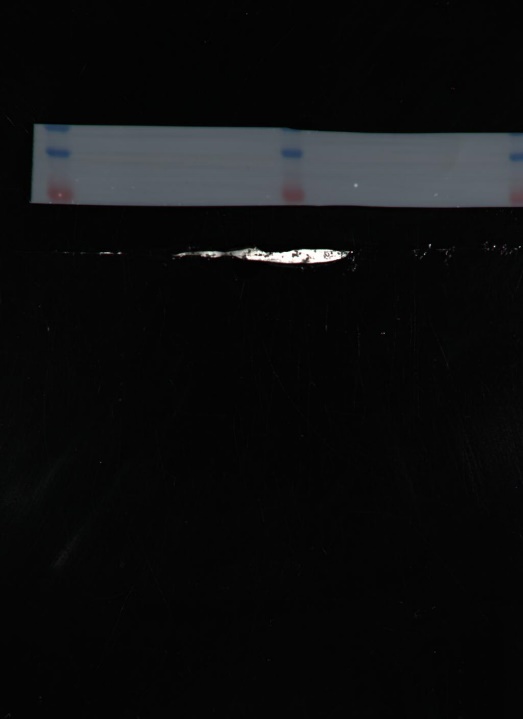


**Figure8**

p-DRP1
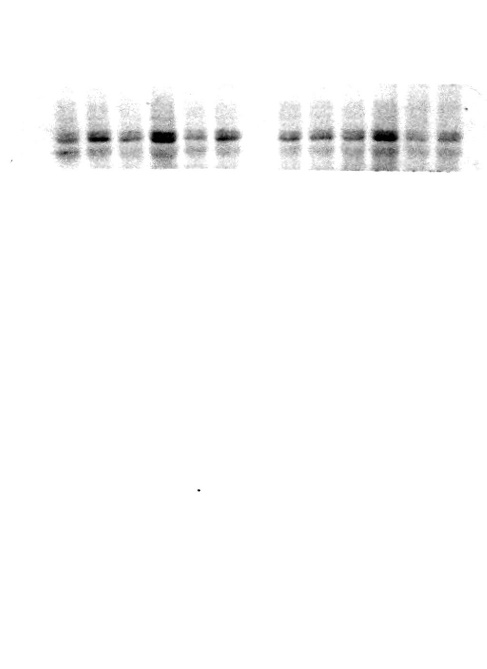

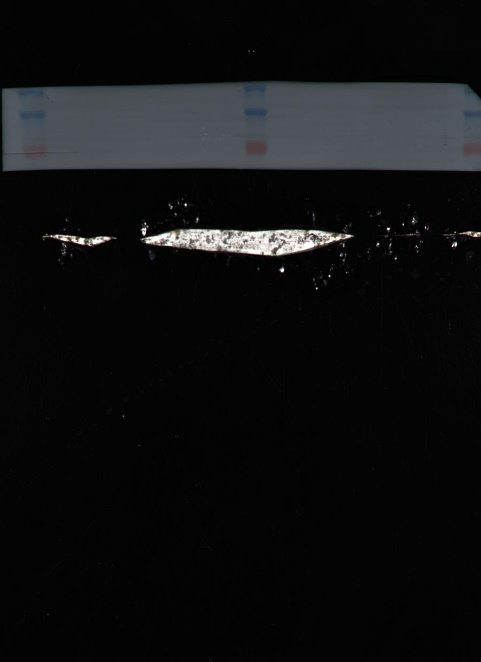


DRP1
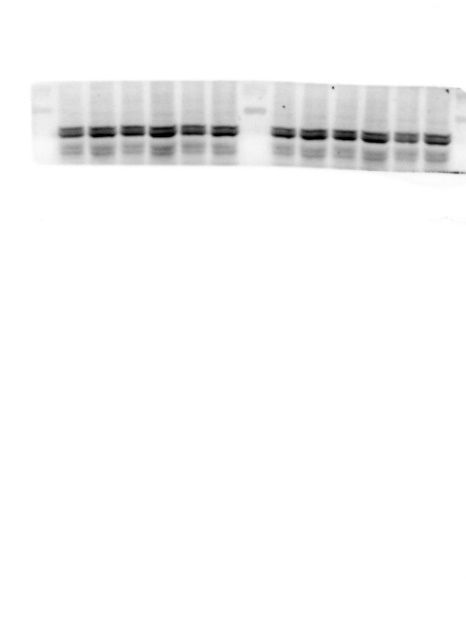

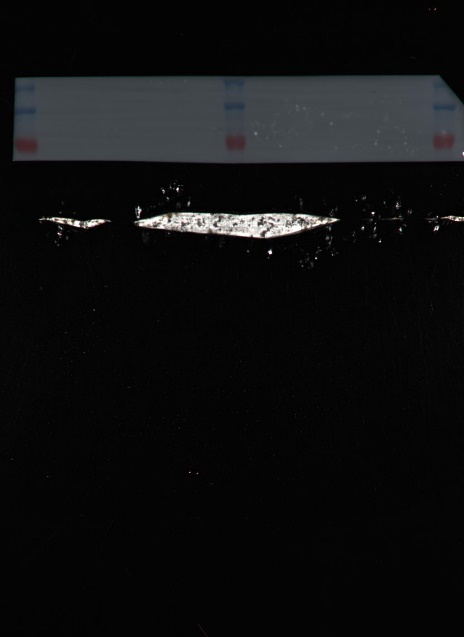


GAPDH
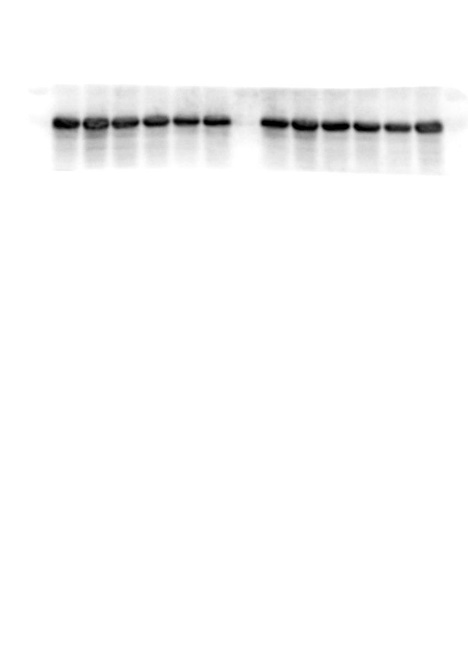

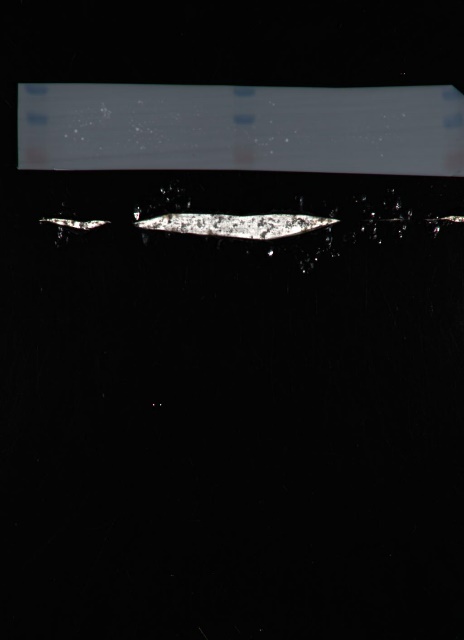


OPA1
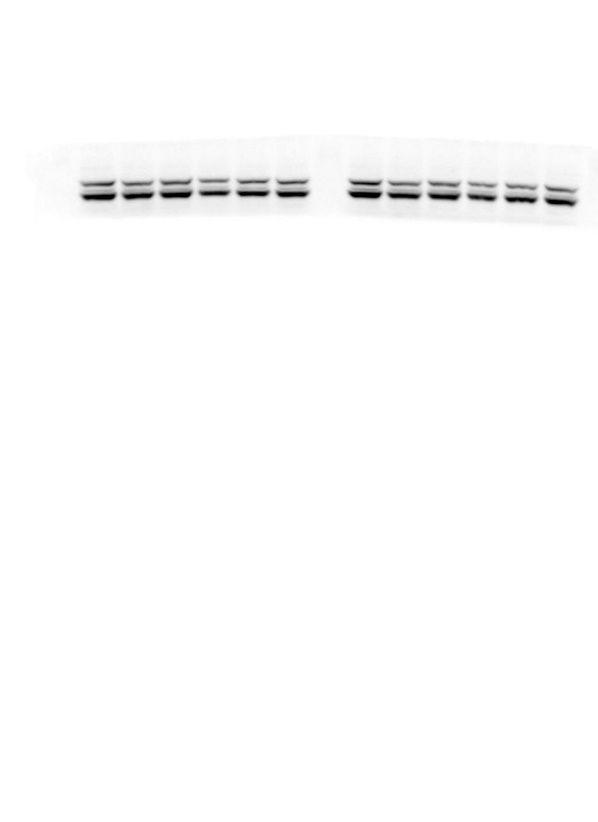

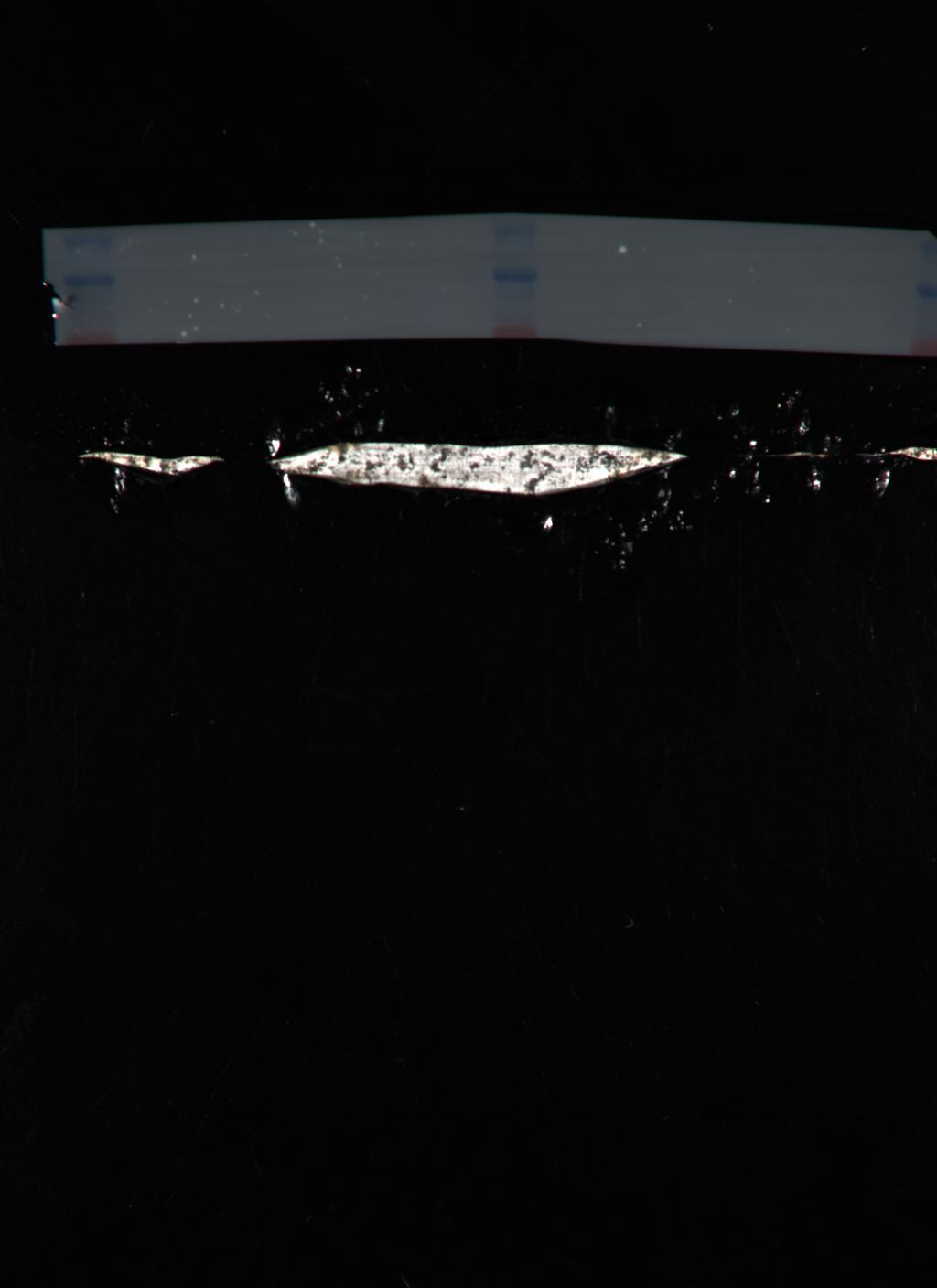


GAPDH
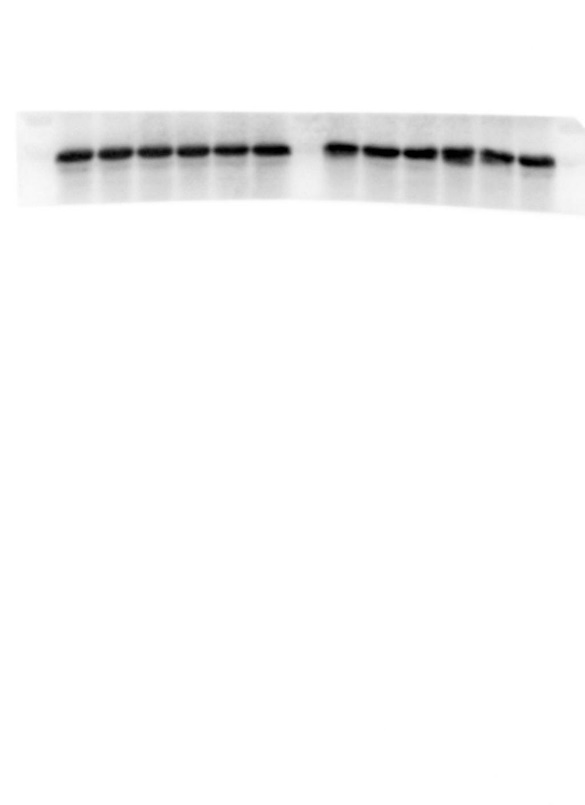

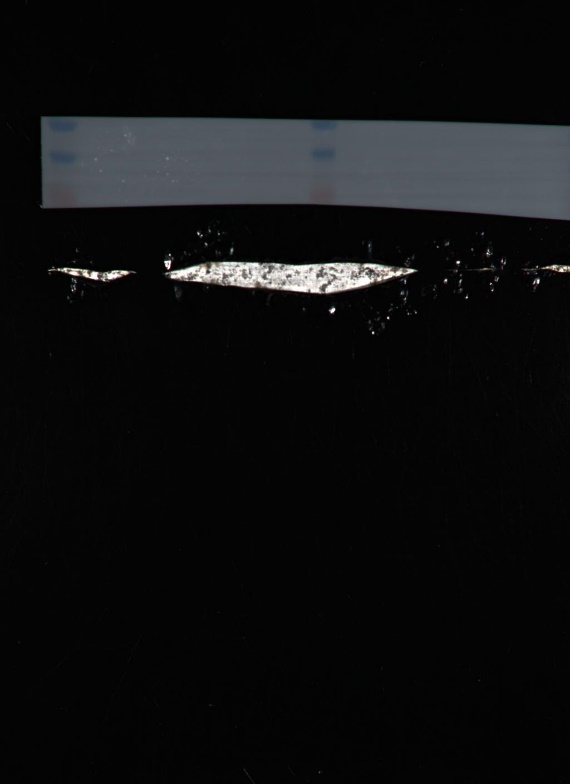


MLKL
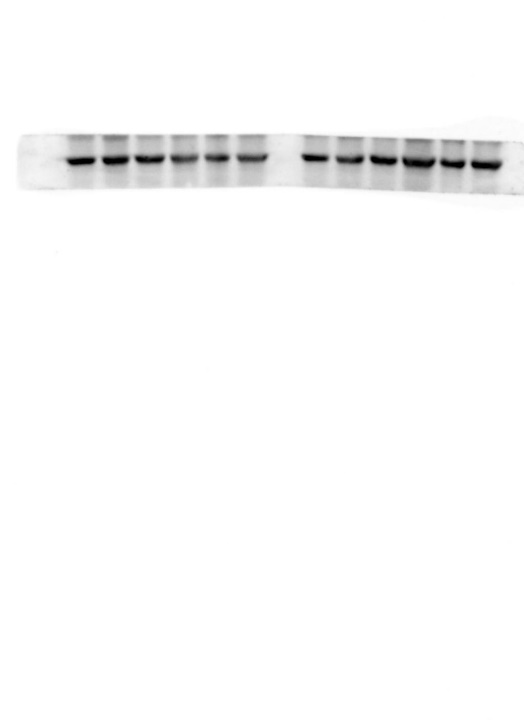

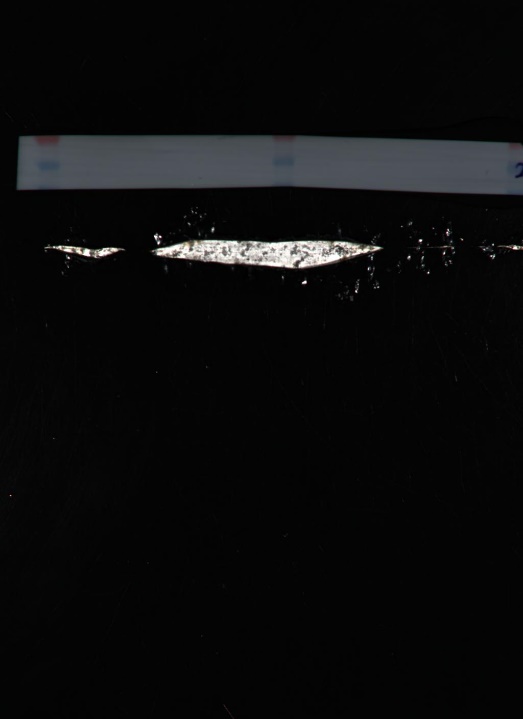


GAPDH
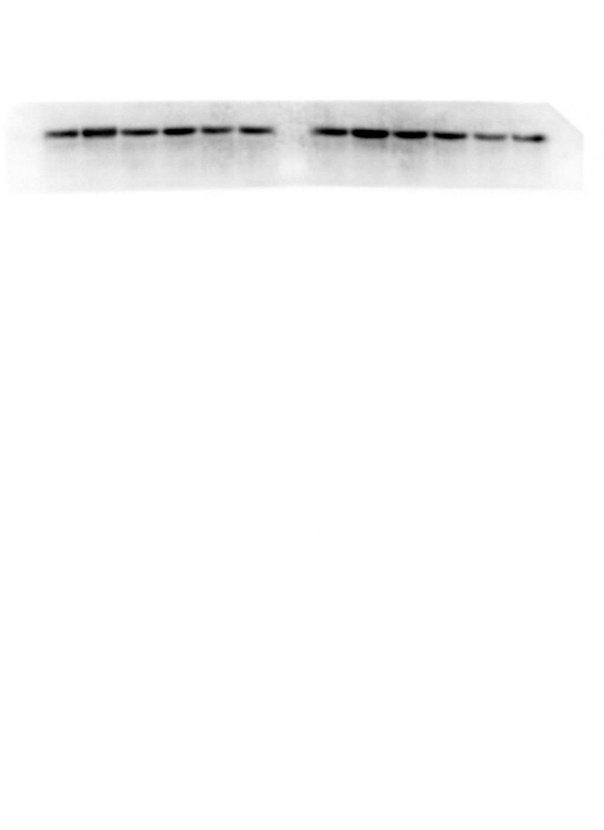

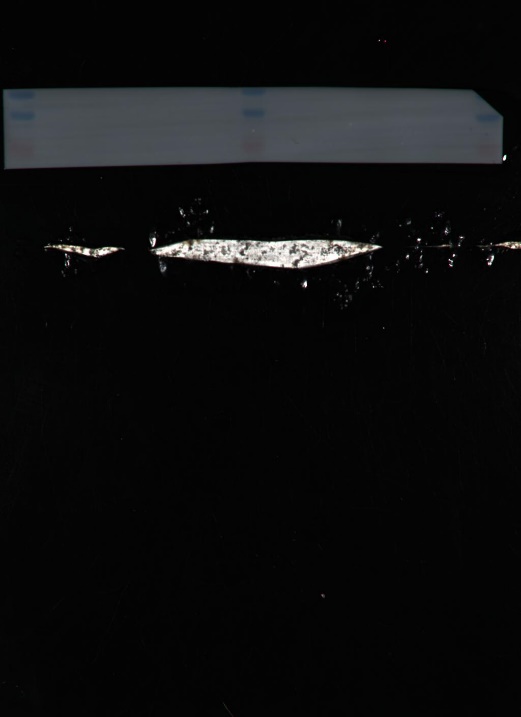


RIPK1
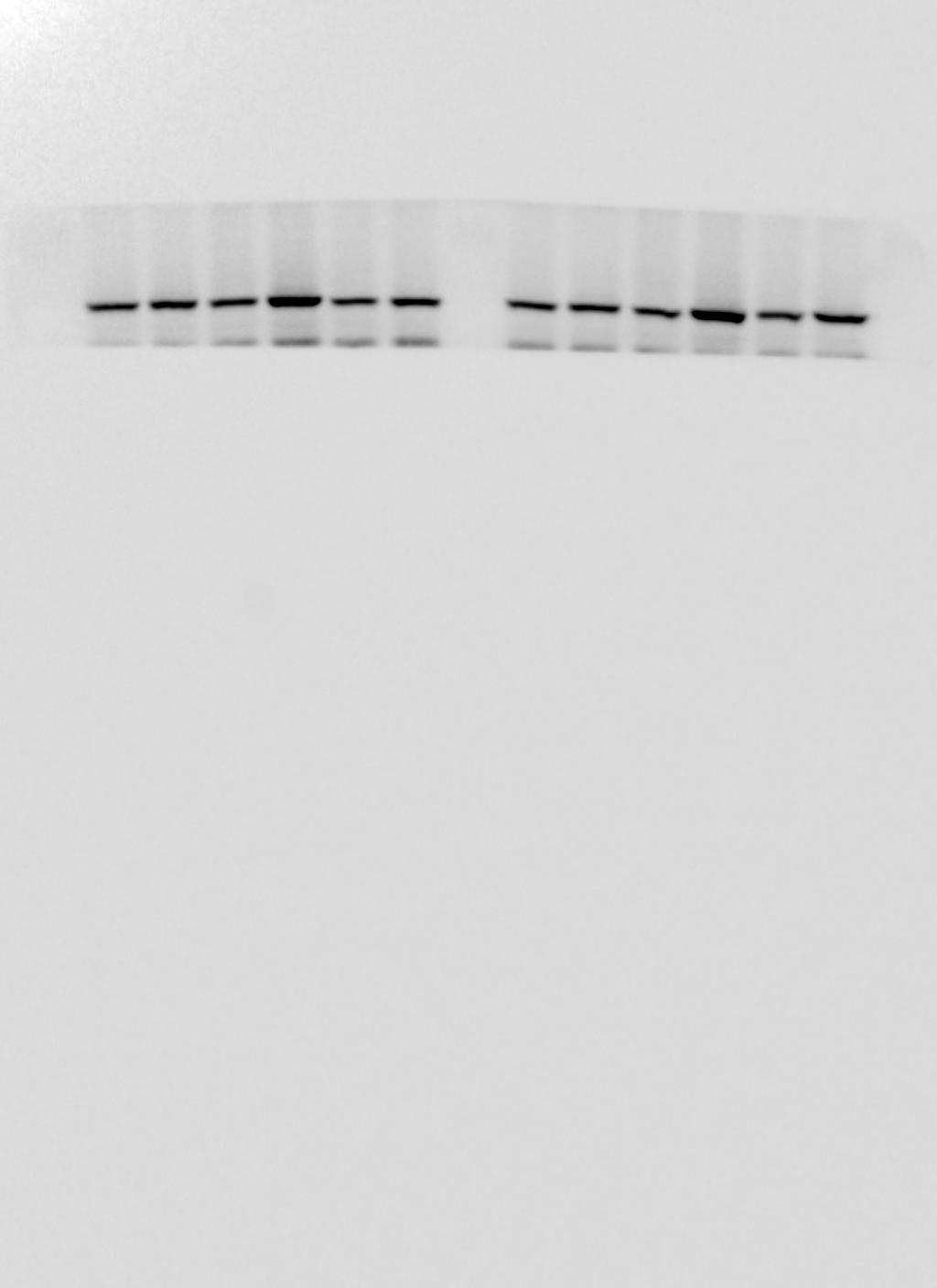

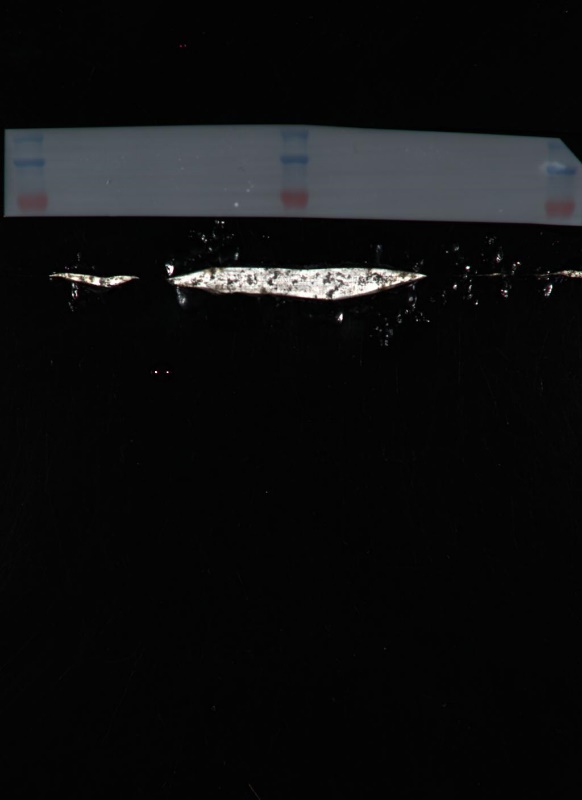


GAPDH
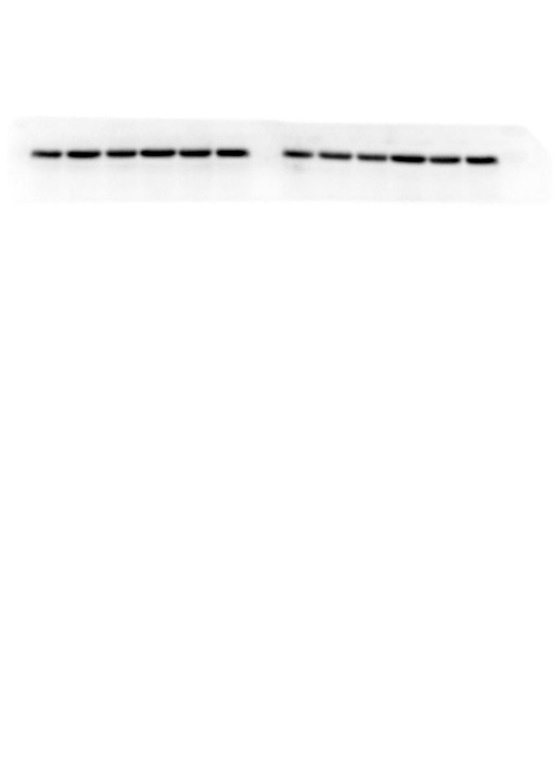

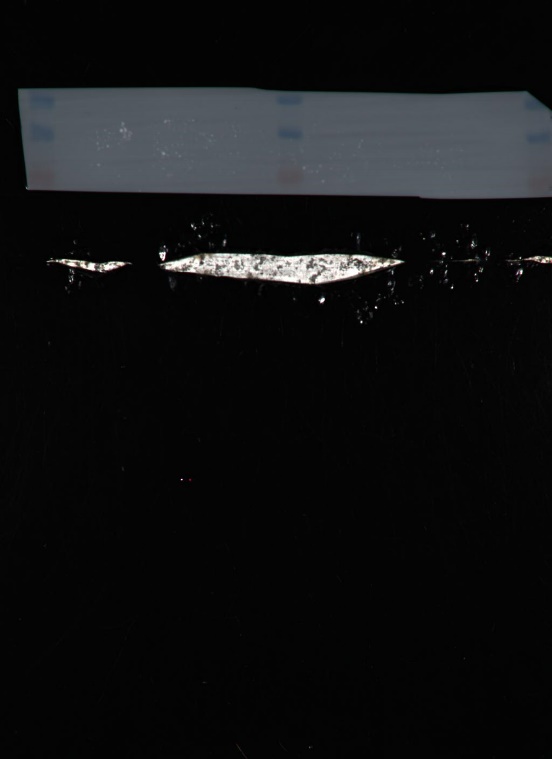


**Figure S4**


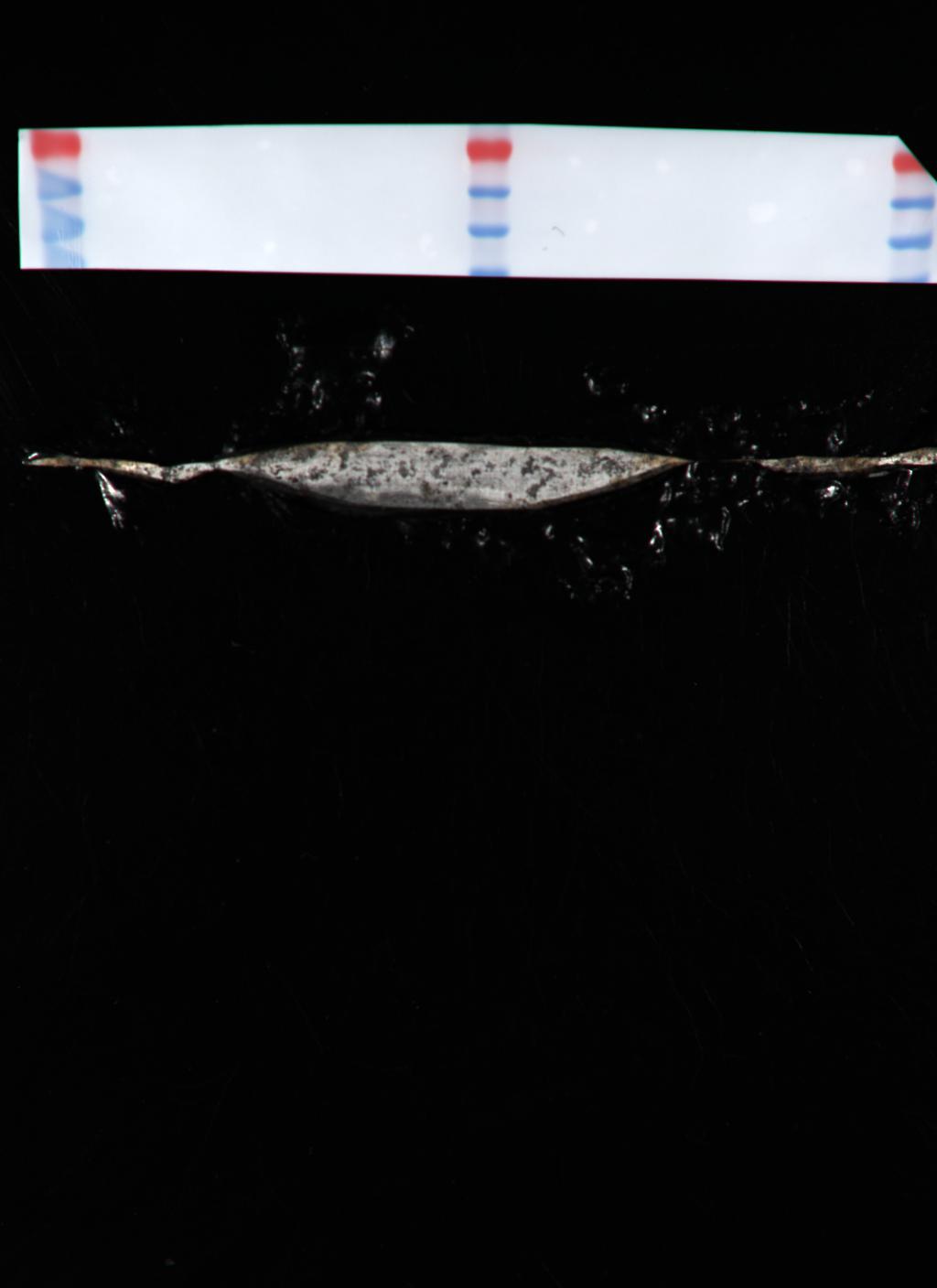

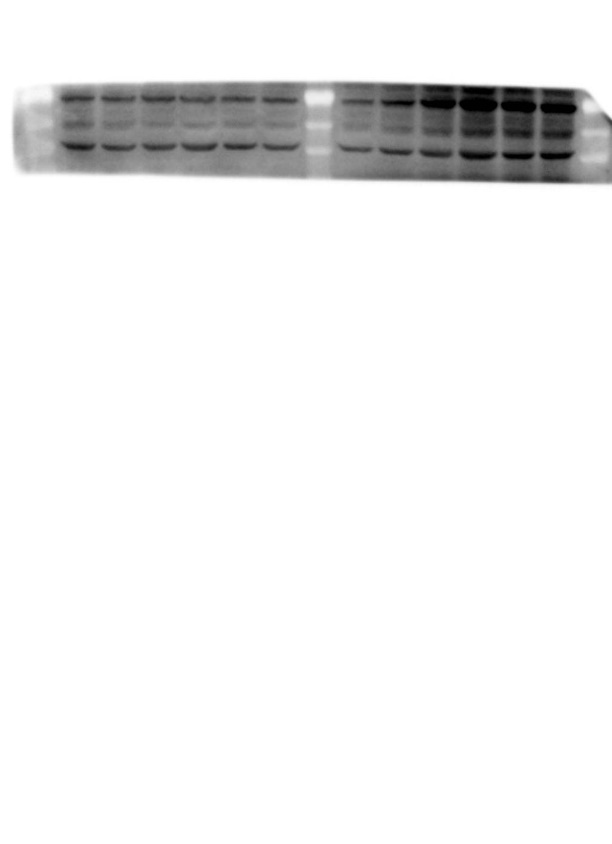
A Caspase1

β-actin


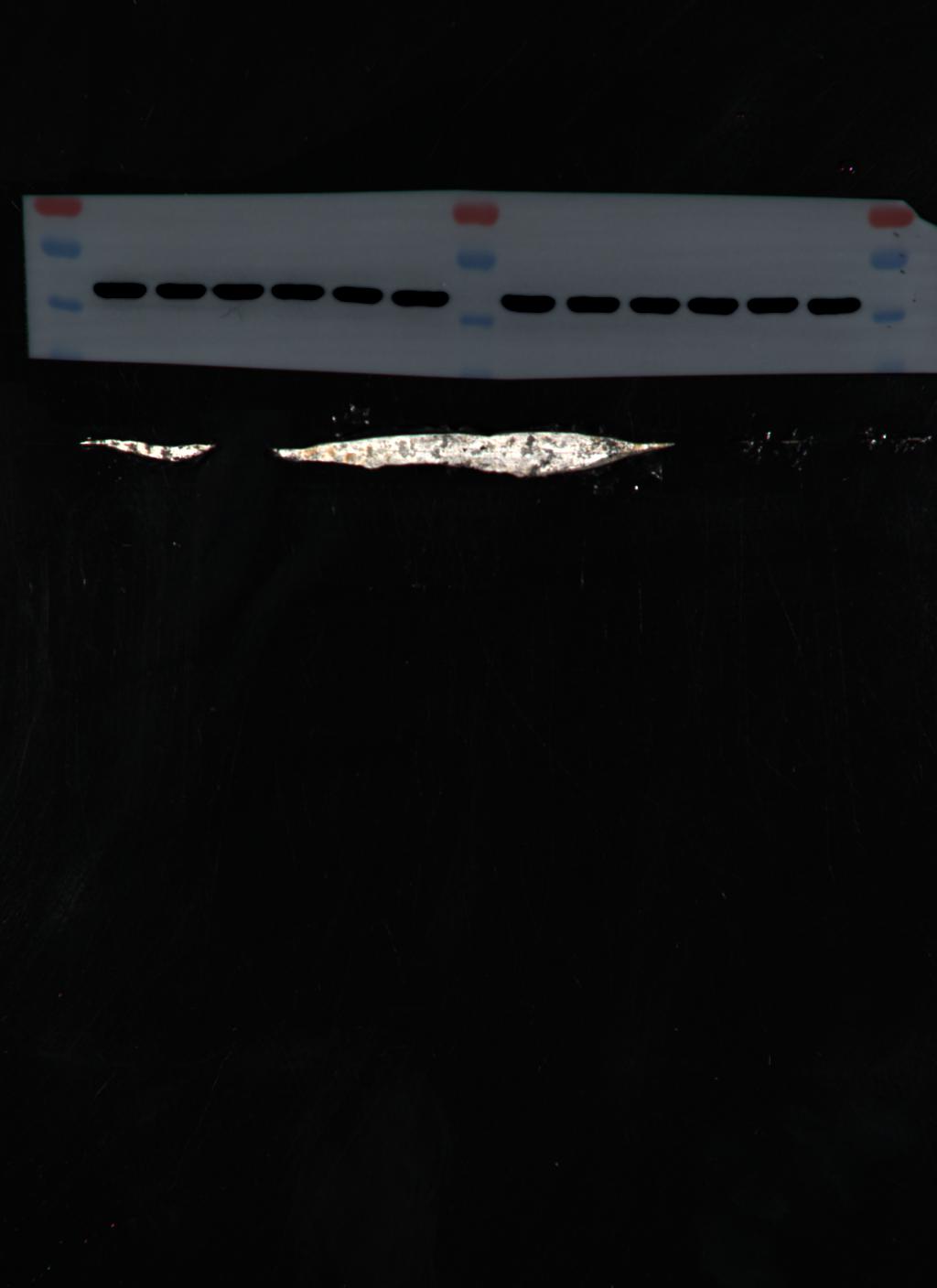


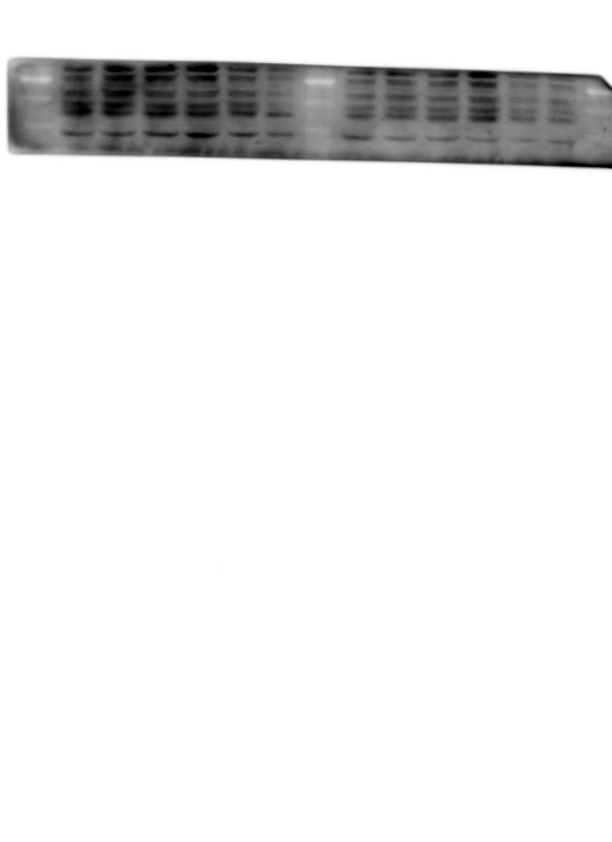

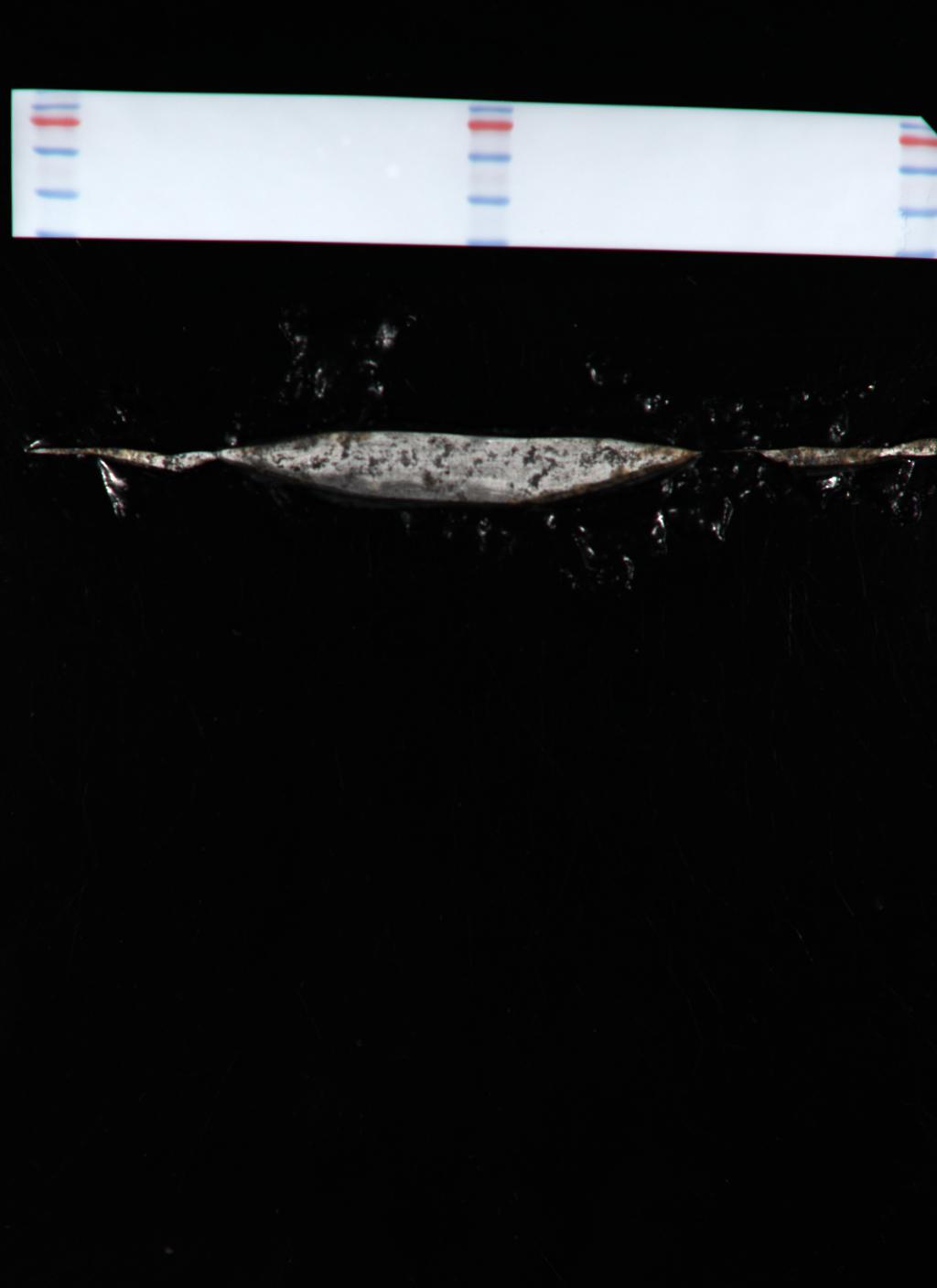
B Caspase1

β-actin


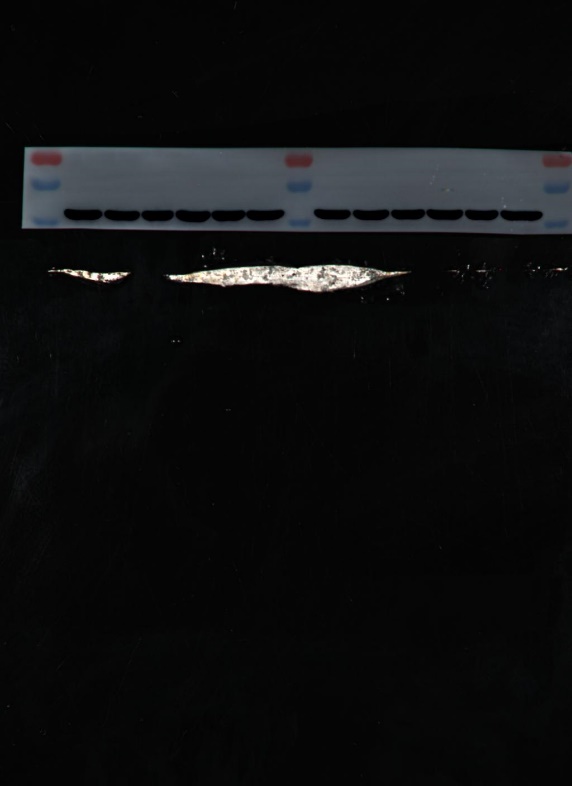


Caspase3 Mdivi-1 BGP-15


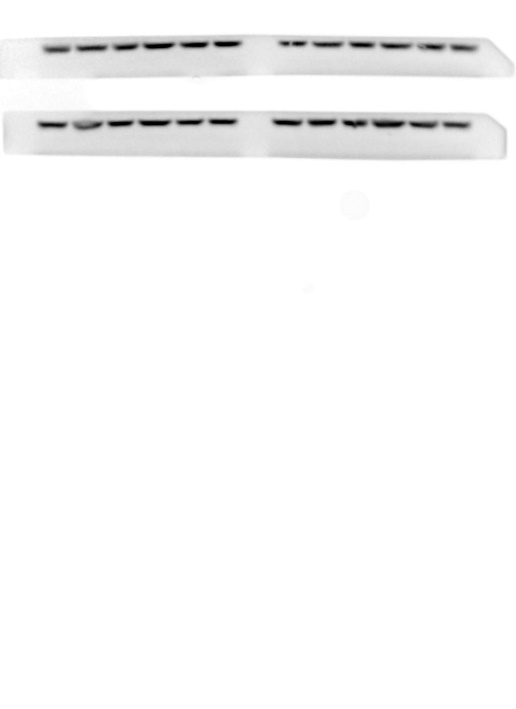

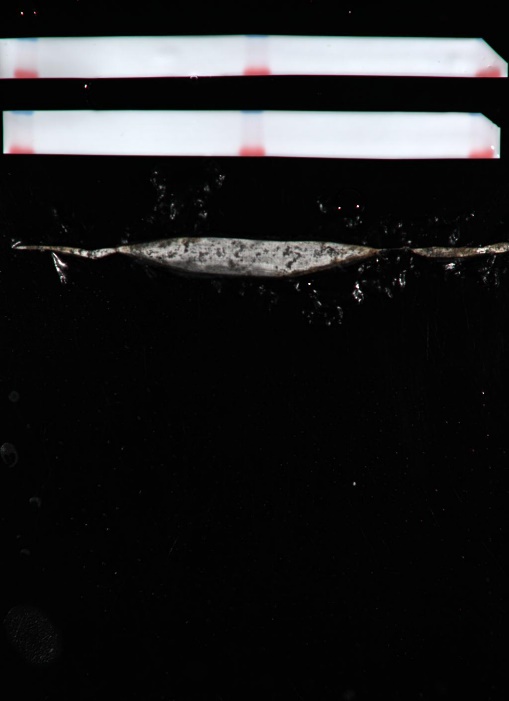


β-actin


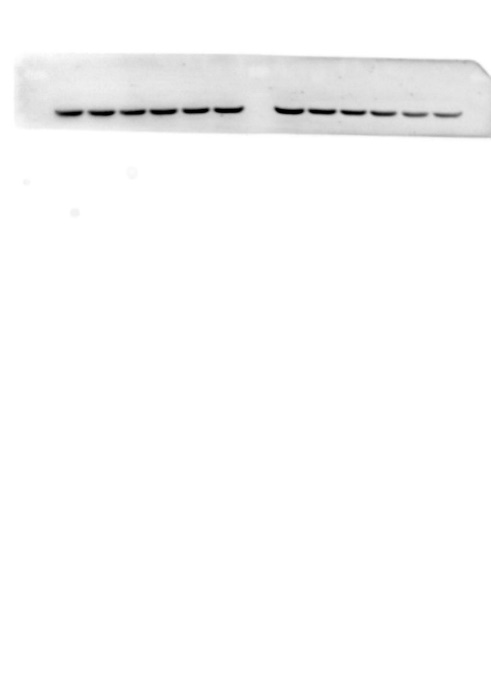

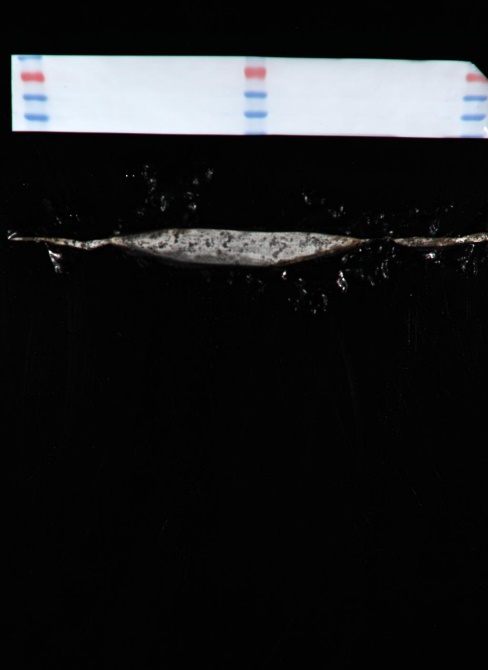


Caspase3 DRP1-KD OPA1-OE


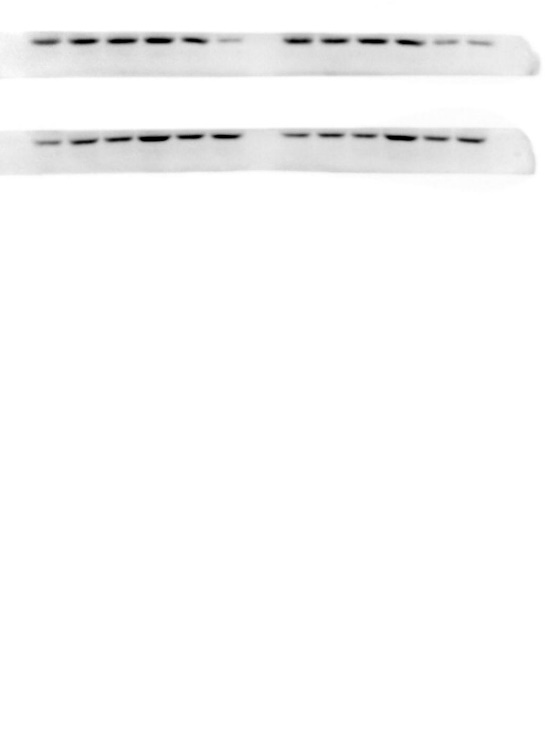

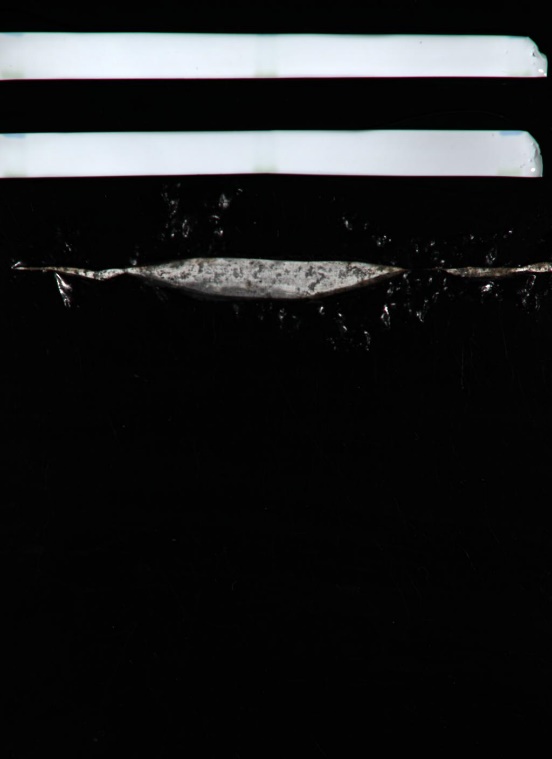


β-actin


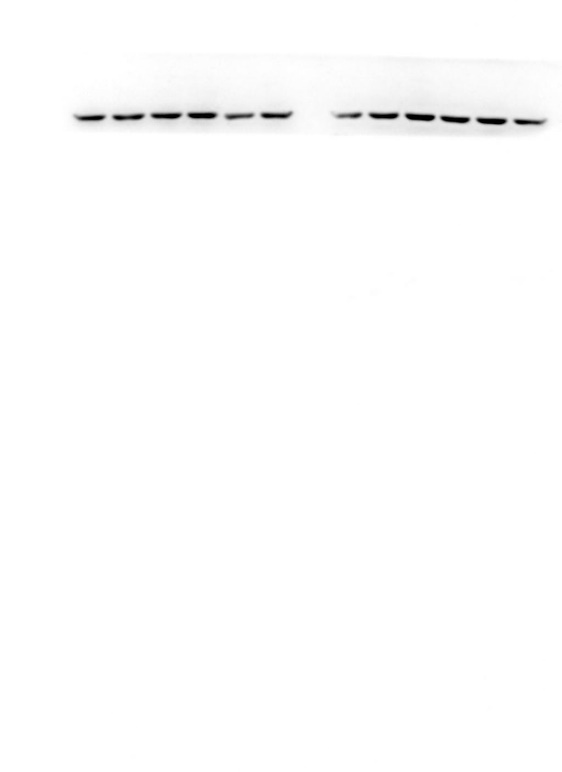

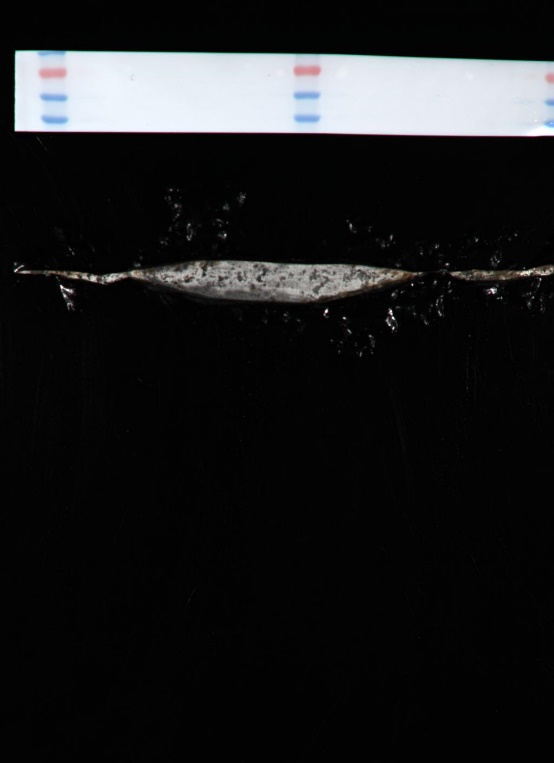


Cleaved Caspase3 Mdivi-1 BGP-15


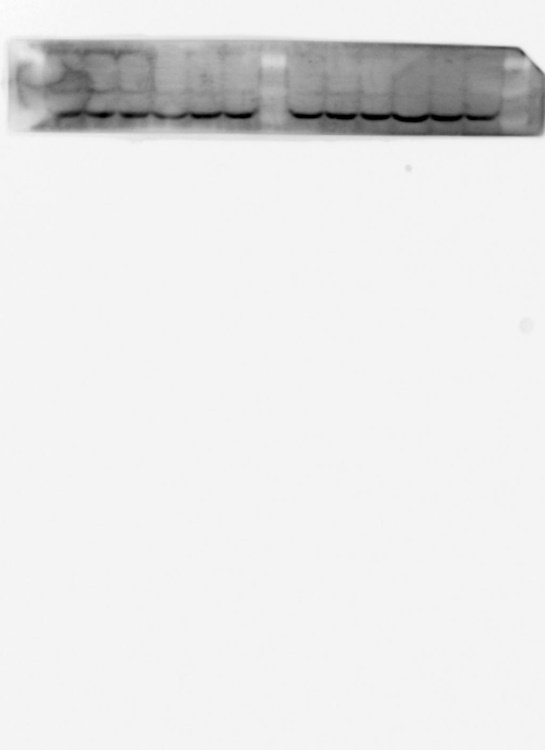

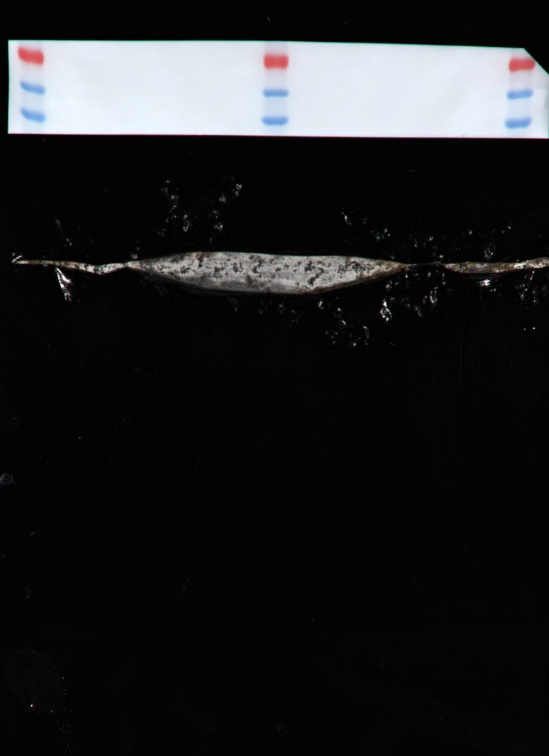


β-actin

Cleaved Caspase3 DRP1-KD OPA1-OE

β-actin
