# Supplementary material for: Attenuation of PM2.5-induced alveolar epithelial cells and lung injury through regulation of mitochondrial fission and fusion
Source: Part Fibre Toxicol. 2023 Jul 18;20:28. doi: 10.1186/s12989-023-00534-w (PMC10353144; doi:10.1186/s12989-023-00534-w)
Supplement: Supplementary file 2 — Supplementary Material 2 [file 12989_2023_534_MOESM2_ESM.docx]

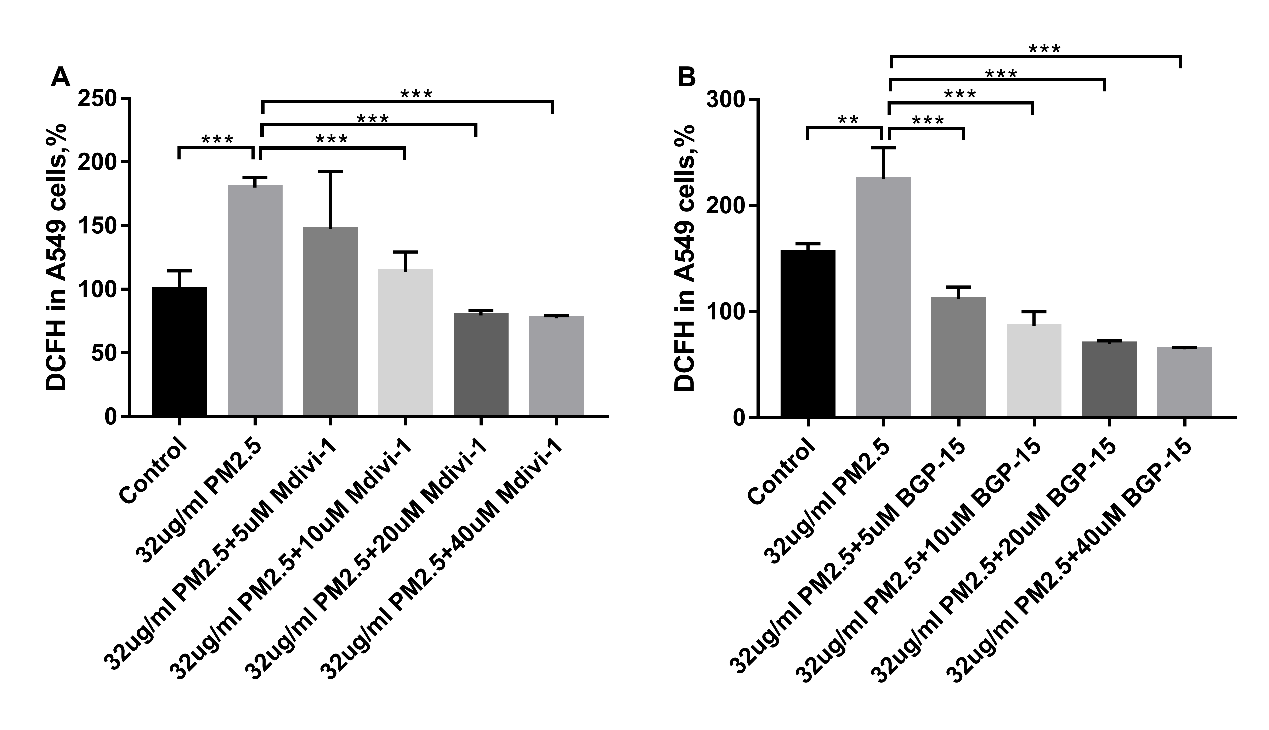


**Figure S1.** Effect of Mdivi-1 (**A**) and BGP-15 (**B**) on PM_2.5_-induced intracellular ROS in A549 cells. Mean ± SD values of data from 4 individual experiments are shown. One-way ANOVA with Bonferroni’s post hoc test (for equal variance) or Dunnett’s T3 post hoc test (for unequal variance) was performed for comparisons among multiple groups. *P<0.05, **P<0.01, ***P<0.001.

**
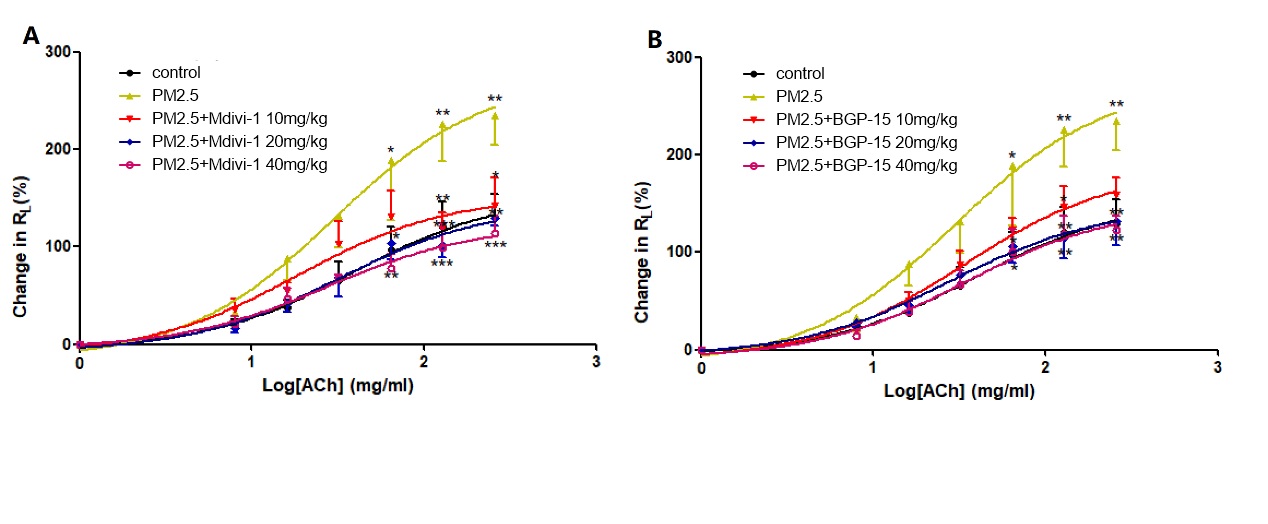
**

**Figure S2.** Mean percentage increase in lung resistance (RL) to increasing concentrations of acetylcholine (ACh) in mice following treatment with PM_2.5_. The effect of increasing doses (mg/kg) of (**A**) Mdivi-1 and of (**B**) BGP-15 are shown. Two-way ANOVA was performed for comparisons of %change in lung resistance between individual groups. One-way ANOVA with Bonferroni's post hoc test (for equal variance) or Dunnett's T3 post hoc test (for unequal variance) was performed for comparisons among multiple groups. Results are presented as the means±SD from experiments conducted in 4 mice. *P<0.05, **P <0.01, ***P <0.001.


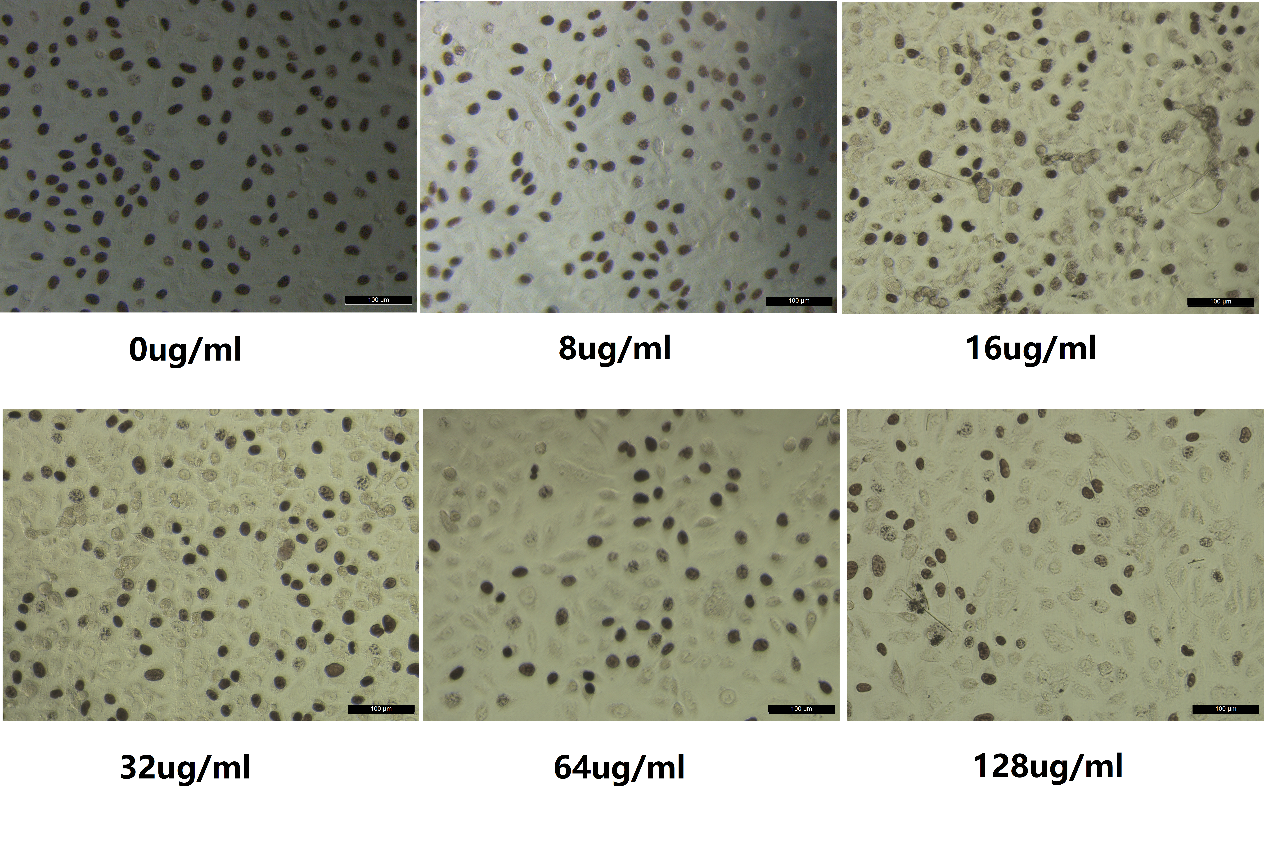


**Figure S3.** Brightfield microscopy images of A549 cells under 0-128ug/ml PM2.5 exposure, with EdU incorporated into the nucleus showing brown precipitation(original magnification, x20).

*
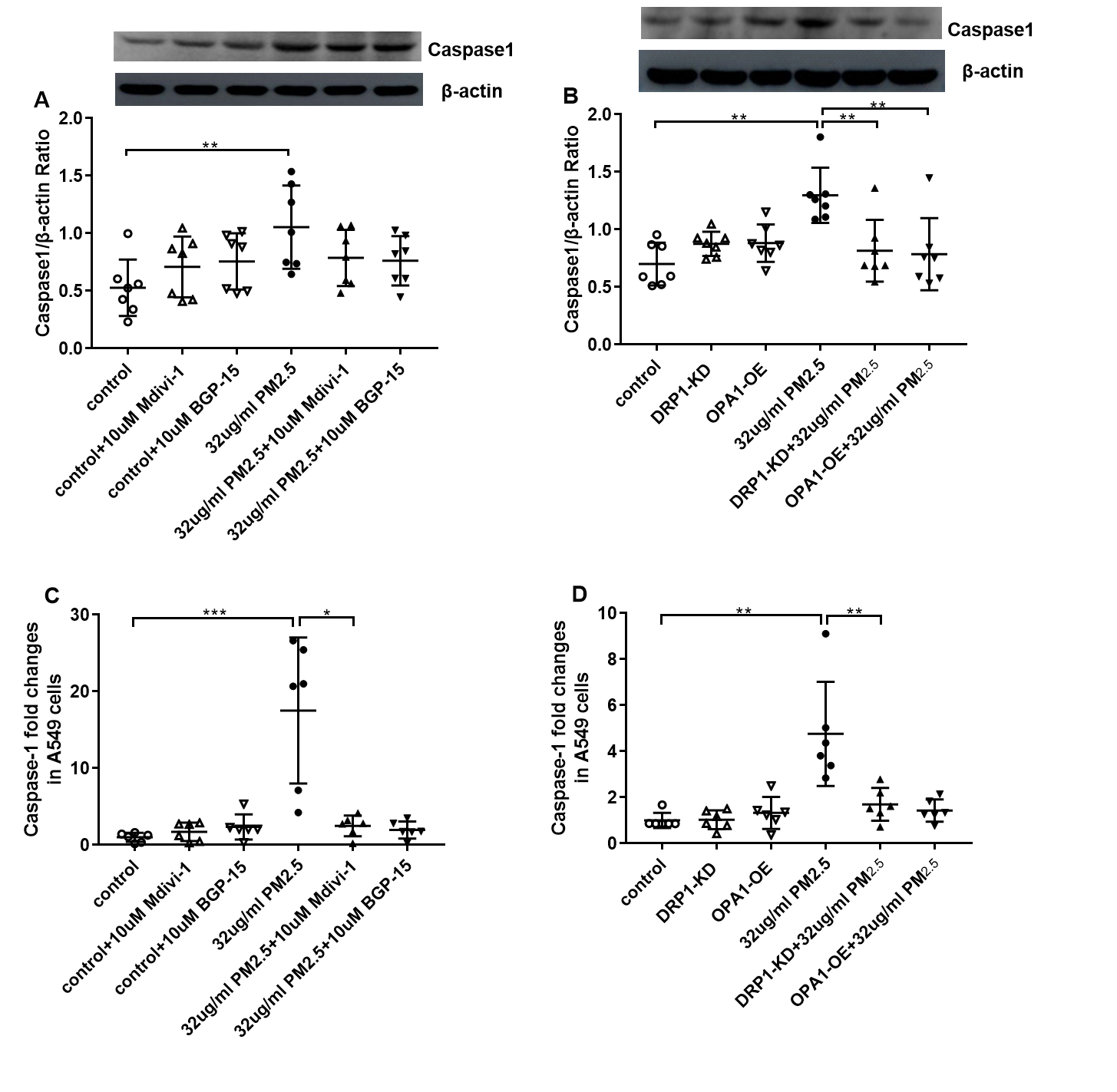
*

**Figure S4.** (A) Western blot analysis of Caspase-1 after exposure to PM_2.5_ or pretreatment with Mdivi-1 or BGP-15 in A549 cells. (B)Western blot analysis of the Caspase-1 after exposure to PM_2.5_ in DRP1-KD and OPA1-OE cells. One-way ANOVA with Bonferroni’s post hoc test (for equal variance) or Dunnett’s T3 post hoc test (for unequal variance) was performed for comparisons among multiple groups. *P<0.05, **P<0.01, ***P<0.001.


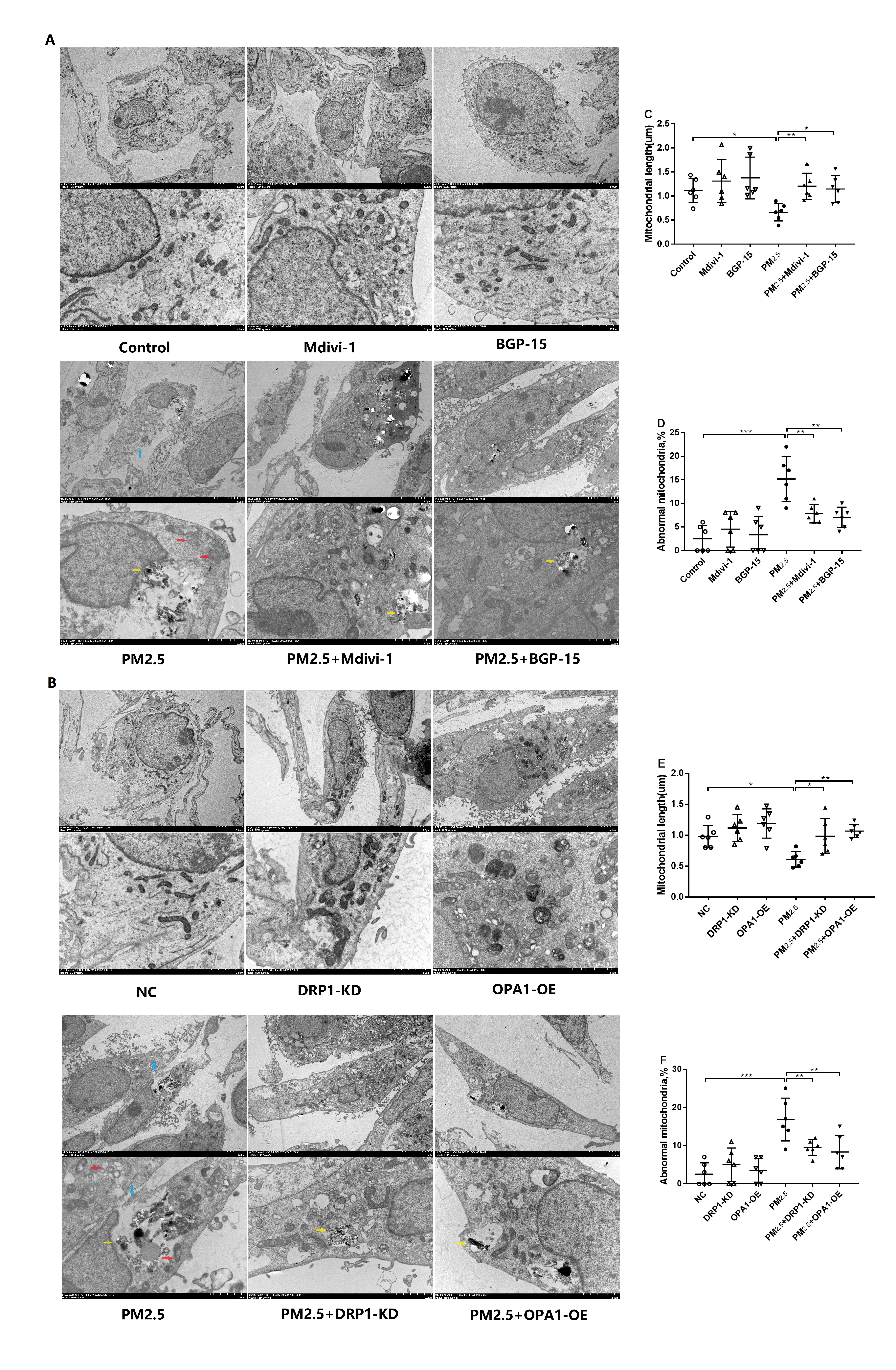


**Figure S5.** **Effect of PM2.5, Mdivi-1, BGP-15, DRP1-KD and OPA1-OE on Transmission electron microscopy (TEM) analysis in cells.** Representative ultrastructure observations of mitochondrial morphology in PM2.5-exposed cells in the presence or absence of Mdivi-1 or BGP-15 in A549 cells **(A, x4000/ x12000/ x15000)**. Representative ultrastructure observations of mitochondrial morphology in PM2.5-exposed cells in the presence or absence of DRP1- KD or OPA1-OE**(B, x4000/ x12000/ x15000)**. Yellow arrows indicate endocytosed PM2.5, red arrows indicate abnormal mitochondria, and blue arrows indicate broken cell membranes. Quantitative analysis of the mitochondrial length**(C,E)** of each group and the percentage of abnormal mitochondria to the total number of mitochondria**(D,F)**. One-way ANOVA with Bonferroni’s post hoc test (for equal variance) or Dunnett’s T3 post hoc test (for unequal variance) was performed for comparisons among multiple groups. *P<0.05, **P<0.01, ***P<0.001.
